# Ir(iii)/Ag(i)-catalyzed directly C–H amidation of arenes with OH-free hydroxyamides as amidating agents†

**DOI:** 10.1039/d4ra00517a

**Published:** 2024-02-15

**Authors:** Youpeng Zuo, Meijun Liu, Jun Du, Tianren Zhang, Xiaoqing Wang, Cong Wang

**Affiliations:** a School of Chemistry and Chemical Engineering, Suzhou University Suzhou 234000 P. R. China 812901107@qq.com

## Abstract

A versatile Ir(iii)-catalyzed C–H amidation of arenes by employing readily available and stable OH-free hydroxyamides as a novel amidation source. The reaction occurred with high efficiency and tolerance of a range of functional groups. A wide scope of aryl OH-free hydroxyzamides, including conjugated and challenging non-conjugated OH-free hydroxyzamides, were capable of this transformation and no addition of an external oxidant is required. This protocol provided a simple, straightforward and economic method to a variety *N*-(2-(1*H*-pyrazol-1-yl)alkyl)amide derivates with good to excellent yield. Mechanistic study demonstrated that reversible C–H bond functionalisation might be involved in this reaction.

## Abstract 2

A versatile Ir(iii)-catalyzed C–H amidation of arenes by employing readily available and stable OH-free hydroxyamides as a novel amidation source.

## Graphical abstract

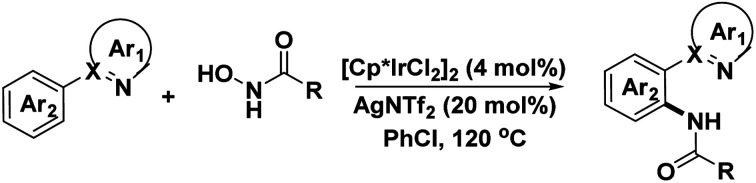

## Introduction

Pyrazole and its derivatives play crucial roles in organic and pharmaceutical fields, because of their unique five-membered nitrogen-containing heterocyclic structures.^1^ In synthetic chemistry, pyrazole and its derivatives are not only efficiently and conveniently synthesized, but also further transformed into more complex artificial molecules.^2^ In the continuous development of coordination chemistry, various complexes containing pyrazole ligands have also been synthesized, characterized, and further applied.^3^ In the field of biomedicine, pyrazole and its derivatives have also been proven to have good antibacterial, anti-inflammatory, and anti-tumor activities.^4^ Because of the multi-reactivity and practicality profile, there is a continued strong demand for efficient and selective synthesis of pyrazole and its derivatives for theoretical and practical research. The most common synthesis methods mainly focus on the following parts: (1) Knorr pyrazole synthesis reaction and further expanded to α,β-unsaturated carbonyl compounds;^5^ (2) pyrazole and its derivatives can also be obtained from the reaction of hydrazones and ketones, alkynes, and isonitriles catalyzed by metal-free conditions;^6^ (3) direct functionalization reactions of pyrazole and its derivatives.^7^ Based on previous work, there has been good progress in the research of transition metal catalyzed pyrazole synthesis methodologies.^8^ Actually, cost-effective, and feasibility degree synthesis methods are still needful, in view of the broad range of applications of pyrazole in biology and chemistry.

**Scheme 1:**
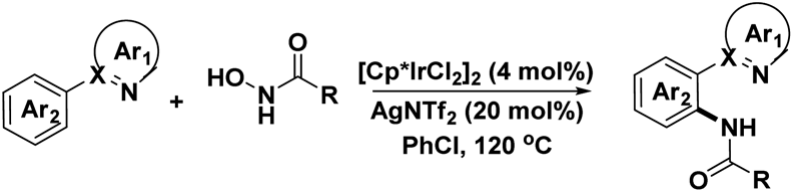
Ir(iii)/Ag(i)-catalyzed C–H directly amination with *N*-hydroxy amide.

Transition-metal-catalyzed C–H functionalization has become powerful tools for synthesis of non-cyclic or cyclic artificial molecule, which complementary to traditional synthesis methods.^9^ C–H functionalization reaction has unparalleled advantages in the construction of chemical bonds and compounds.^10^ Particularly, significant progress has been made in C–H bond amidation reactions in recent years.^11^ Heterocyclic compounds represented by pyrazole are excellent directing groups in C–H bond activation reactions, which catalyzed by Cp*Rh(iii), Cp*Ir(iii), Ru(ii) and other metal.^12^ At the same time, a series of amidation reagents, such as *N*-substituted hydroxylamines,^13^*N*-methoxyamide,^14^ dioxazolones,^15^ organic azide,^16^ chloramines and other substrates,^17^ have been widely used as C–N coupling partners for construction of structurally complex scaffolds. However, the application of OH-free hydroxyamide as a novel amidation reagent in C–H bond activation are relatively limited. Therefore, the development of an efficient one pot method to give *ortho*-functionalized pyrazole derivatives *via* Ir(iii)/Ag(i)-catalyst C–H bond amination reactions of *N*-arylpyrazoles and OH-free hydroxyamides. In this methodology, OH-free hydroxyamides were innovatively used as the amidation source, which has excellent selectivity, stability and reactivity compared to organic azides *via* comparative experiment (Scheme 1).

## Results and discussion

We started our investigation by submitting the model substrate *N*-phenyl-pyrazole (1a) and *N*-hydroxybenzamide (2a). The reaction carried out with [Cp*IrCl_2_]_2_ (4 mol%) as catalyst, AgNTf_2_ as an (20 mol%) additive, without any external base and oxidant, in 1,2-DCE at 90 °C for 8 h under air. As result, the desired amidated product (3aa) was obtained in 57% isolated yield without any bisamidated product was detected. After constructing this feasible concept, the confirmed results encourage us to further conduct optimization studies with an initial focus on the catalyst, among the various catalyst investigated, the [Cp*IrCl_2_]_2_ was found most efficient, other metal catalyst such as[Cp*RhCl_2_]_2_, Pd(OAc)_2_ and Pd(PPh_3_)_4_ could not promoted this transformation (Table 1, entry 1–4). The control experiment further confirmed no amidating product could be obtained without [Cp*IrCl_2_]_2_ or AgNTf_2_ (Table 1, entry 5–6). In order to improve the yield of the reaction, several solvents were taken into consideration, such as toluene, PhCl, MeCN, DMSO and 1,4-dioxane were screened (Table 1, entry 7–11), wherein PhCl was the best choice with 67% isolated yield. Subsequently, a variety of additive were used, unfortunately, none of them worked (Table 1, entry 12–17). The effect of temperate variation was also investigated and increasing it to 120 °C could assisted the transformation. However, further increase of temperature the yield with slightly decrease (Table 1, entry 18–21). In addition, reducing or increasing catalyst, additive and time were detrimental to the yield (Table 1, entry 22–28).

**Table 1:** Optimization of the reaction conditions^a^

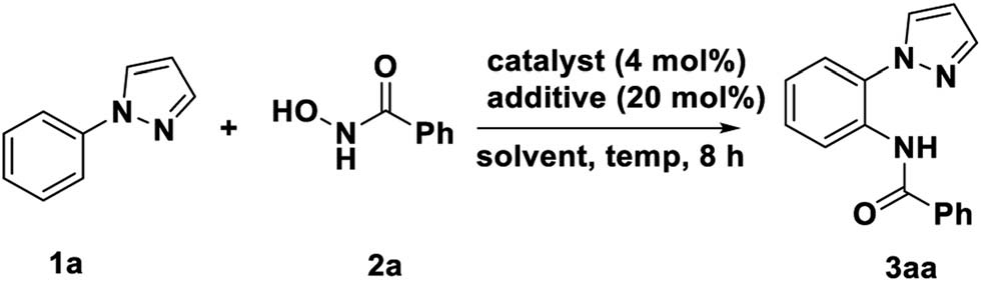					
Entry	Catalyst	Additive	Solvent	Temp (^o^C)	Yield^b^ (%)
1	[Cp*IrCl_2_]_2_	AgNTf_2_	1,2-DCE	90	57
2	[Cp*RhCl_2_]_2_	AgNTf_2_	1,2-DCE	90	Trace
3	Pd(OAc)_2_	AgNTf_2_	1,2-DCE	90	Trace
4	Pd(PPh_3_)_4_	AgNTf_2_	1,2-DCE	90	Trace
5	—	AgNTf_2_	1,2-DCE	90	Trace
6	[Cp*IrCl_2_]_2_	—	1,2-DCE	90	Trace
7	[Cp*IrCl_2_]_2_	AgNTf_2_	Toluene	90	46
8	[Cp*IrCl_2_]_2_	AgNTf_2_	PhCl	90	67
9	[Cp*IrCl_2_]_2_	AgNTf_2_	MeCN	90	61
10	[Cp*IrCl_2_]_2_	AgNTf_2_	DMSO	90	Trace
11	[Cp*IrCl_2_]_2_	AgNTf_2_	1,4-Dioxane	90	23
12	[Cp*IrCl_2_]_2_	AgSbF_6_	PhCl	90	Trace
13	[Cp*IrCl_2_]_2_	AgOAc	PhCl	90	Trace
14	[Cp*IrCl_2_]_2_	Ag_2_CO_3_	PhCl	90	Trace
15	[Cp*IrCl_2_]_2_	Ag_2_O	PhCl	90	Trace
16	[Cp*IrCl_2_]_2_	CsOPiv	PhCl	90	Trace
17	[Cp*IrCl_2_]_2_	HOAc	PhCl	90	Trace
18	[Cp*IrCl_2_]_2_	AgNTf_2_	PhCl	50	28
19	[Cp*IrCl_2_]_2_	AgNTf_2_	PhCl	70	54
**20**	[Cp*IrCl_**2**_ **]**_**2**_	**AgNTf**_**2**_	**PhCl**	**120**	**86**
21	[Cp*IrCl_2_]_2_	AgNTf_2_	PhCl	140	84
22^c^	[Cp*IrCl_2_]_2_	AgNTf_2_	PhCl	120	58
23^d^	[Cp*IrCl_2_]_2_	AgNTf_2_	PhCl	120	83
24^e^	[Cp*IrCl_2_]_2_	AgNTf_2_	PhCl	120	42
25^f^	[Cp*IrCl_2_]_2_	AgNTf_2_	PhCl	120	67
26^gg^	[Cp*IrCl_2_]_2_	AgNTf_2_	PhCl	120	62
27^h^	[Cp*IrCl_2_]_2_	AgNTf_2_	PhCl	120	56
28^i^	[Cp*IrCl_2_]_2_	AgNTf_2_	PhCl	120	86

a Reaction conditions: *N*-phenylpyrazole 1a (0.25 mmol), *N*-hydroxybenzamide 2a (0.25 mmol), solvent (1.5 mL), and catalyst (4.0 mol%) for 8 h.

b Isolated yields.

c 2 mol% of catalyst was used.

d 8 mol% of catalyst was used.

e 10 mol% of additive was used.

f 40 mol% of additive was used.

g 60 mol% of additive was used.

h reaction time for 4 h.

i reaction time for 12 h.

With the optimized reaction conditions in hand, we next examined the tolerance of this methodology, and the corresponding results are summarized in the Table 2. We first explored the scope of *N*-aryl pyrazole with 2a as coupling partner. It was found that a series of *para*-position substituted underwent smoothly amidation with 2a deliver the corresponding products in good to excellent yields (83–94%) with high tolerance of functional groups. Even for sensitive iodine-substituted substrates also could provide the corresponding product 3la in 87% yield. Especially, the substrates containing trifluoromethyl, Cyano group with 2a deliver the corresponding products 3ma and 3na in 84% and 83% yield, respectively. For the *meta*-substituted substrates, the yields show slightly decreases 3ba, 3ga and 3ha compared with *para*-substituents 3ca, 3ja and 3ka. In the end, we also investigated the reaction activity of naphthalene ring, the product 3oa in 94% yield was obtained and confirmed the structure by single crystal diffraction. A gram-level experiment was conducted, and the corresponding product can still be obtained in 88% yield (1.37 g).

**Table 2:** Scope of *N*-aryl pyrazoles in C–H bond amidation^a^^,^^b^

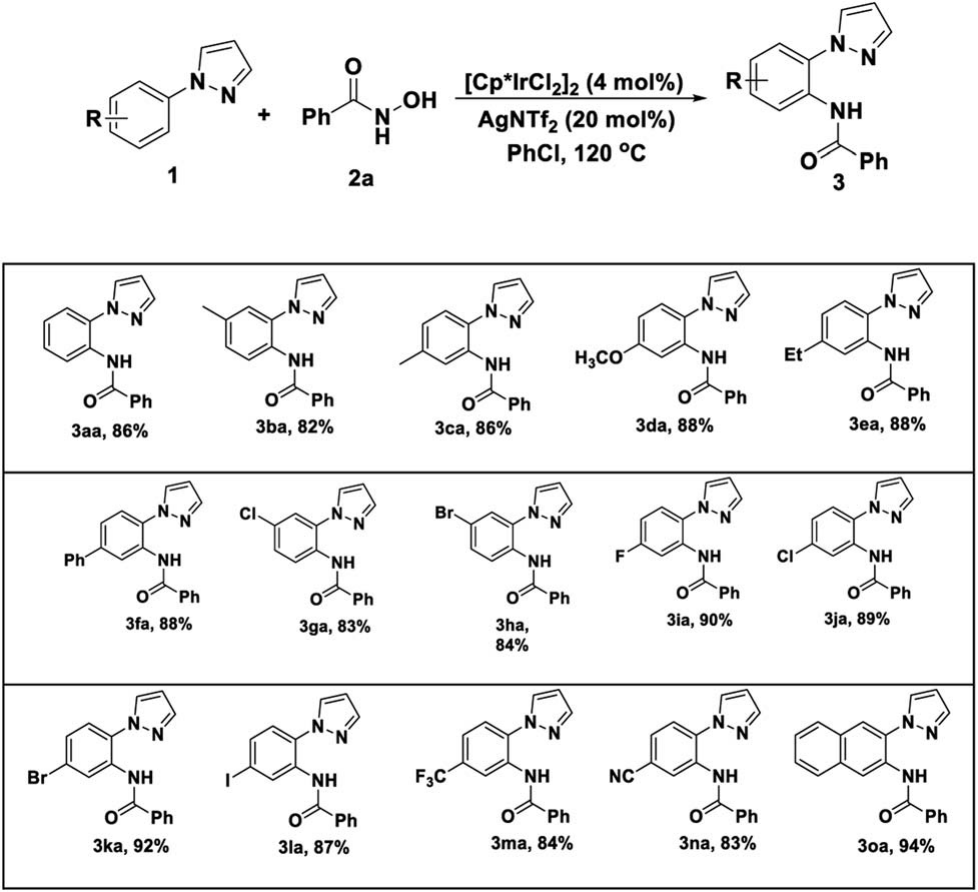

a Reaction conditions: *N*-aryl pyrazoles 1 (0.25 mmol), *N*-hydroxybenzamide 2a (0.25 mmol), [Cp*IrCl_2_]_2_ (4 mol%), and AgNTf_2_ (40 mol%) in PhCl (3 mL) were stirred at 120 °C under air for 8 h.

b Isolated yields.

Encouraged by these positive outcomes, we further examined the selective of the Ir(iii)/Ag(i)-catalyzed C–H bond amidation reaction with *N*-phenyl-pyrazole (1a), a variety of substituted aryl *N*-hydroxyzamide 2 were examined, as shown in Table 3. *N*-hydroxyzamide 2 bearing both electron-donating as well as electron-withdrawing aryl substituents were reacted well with 1a to give the desired amidated products (3ab–3ao) in good to excellent yields. To our delight, bisubstituted aryl *N*-hydroxyzamide could be successfully converted to the corresponding products 3ai and 3aj with more than 80% yield, respectively. What we need to mention that *ortho*-position substituted substrates also provided the corresponding product 3ab and 3ag in 91 and 95% yield, respectively. In addition, when we employed *N*-hydroxycarbamate as amidating agent, there are no amidated products generated under Ir(iii)/Ag(i)-catalyst system.

**Table 3:** Substrates scope of *N*-hydroxyamide^a^^,^^b^

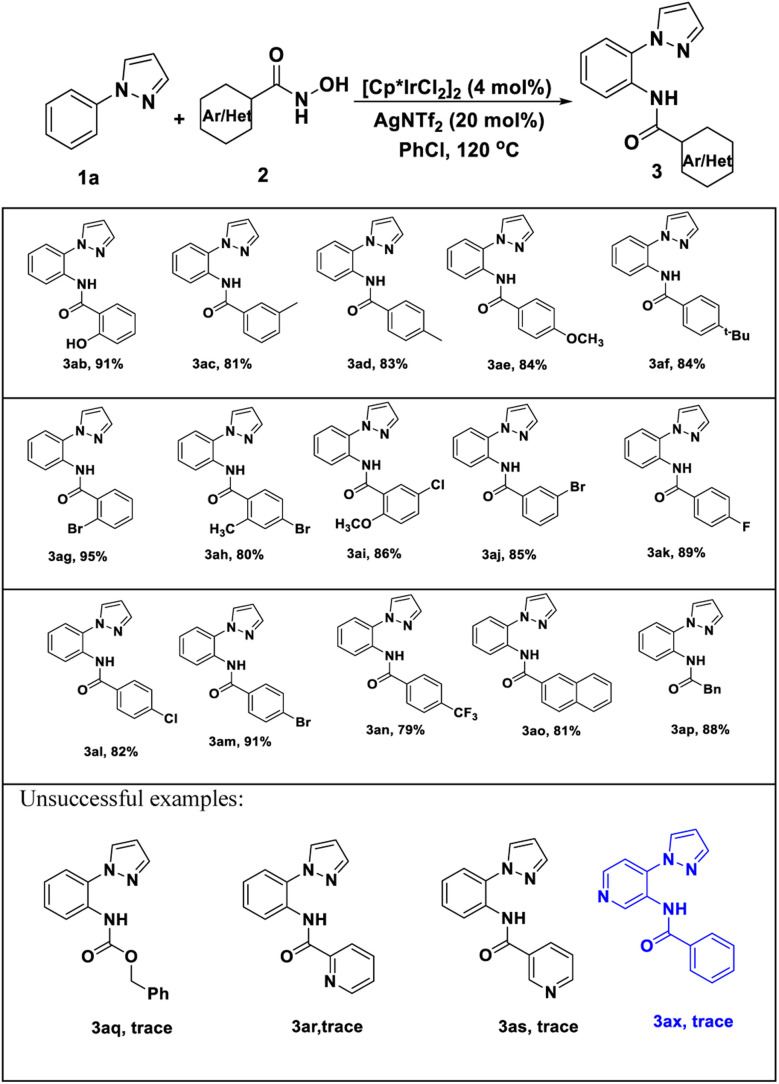

a Reaction conditions: *N*-phenyl-pyrazole 1 (0.25 mmol), *N*-hydroxyamide 2 (0.25 mmol), [Cp*IrCl_2_]_2_ (4 mol%), and AgNTf_2_ (40 mol%) in PhCl (3 mL) were stirred at 120 °C under air for 8 h.

b Isolated yields.

**Scheme 2:**
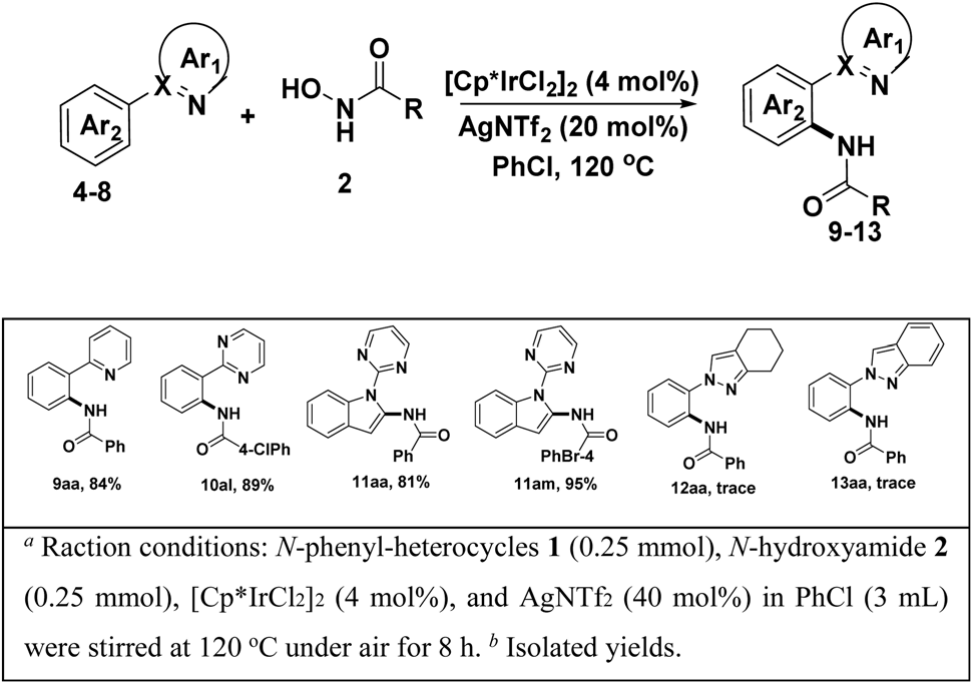
Scope of other *N*-heterocycles-DGs substrates.^*a*,*b*^

To access the general applicability of the protocol, other *N*-heterocycles-DGs substrates were also took into consideration, such as 2-phenylpyridine, 2-phenylpyrimidine and 1-(pyrimidin-2-yl)-1*H*-indole, they could be smoothly transformed into desired amidated products in excellent yield (81–95%) (Scheme 2. 9aa–11am). Unfortunately, other DGs derived from pyrazoles did not work well in the transformation (Scheme 2, 12aa, 13aa).

**Scheme 3:**
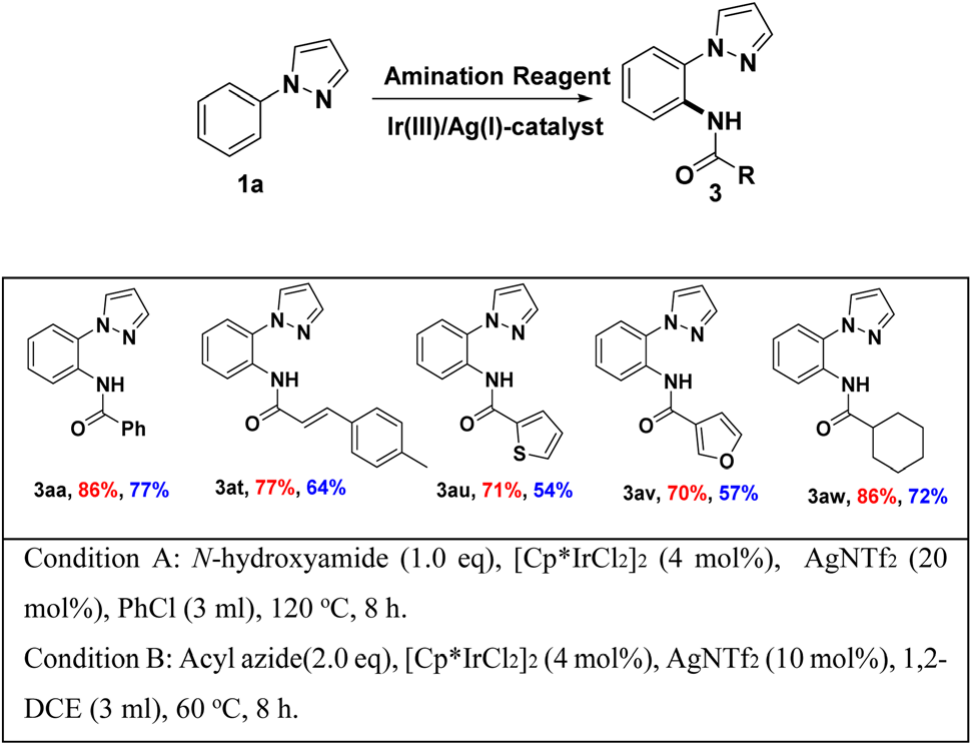
Comparison between acyl azides and *N*-hydroxylamine as amidating agents.^*a*,*b*^

To illustrate the stability and reactivity of *N*-hydroxyamide, we also applied acyl azides under Ir(iii)/Ag(i)-catalyst system. To our delight, desired amidated products were obtained under suitable condition in accepted yield, without any Curtius rearrangement product,^18^ and the results shown in Scheme 5. In the selected example, the yield of the acyl azide as the amidation reagent is lower than that of the *N*-hydroxylamine as the amidation reagent (Scheme 3).

**Scheme 4:**
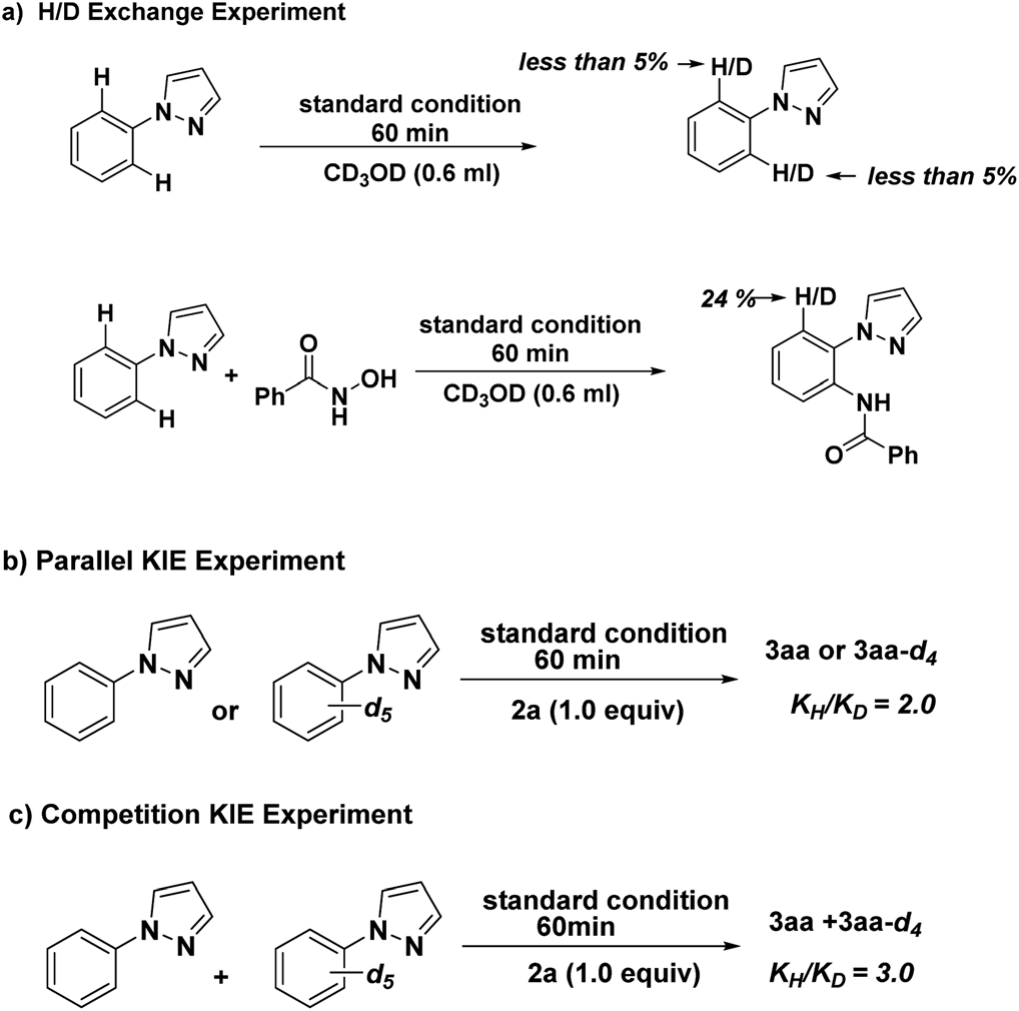
Control experiments.

To investigate the mechanism of this reaction, we carried out a series of control experiments. First, substrate 1a was treated with CD_3_OD under standard reaction conditions. As a result, no D-exchange was detected, suggesting that C–H bond metalation is irreversible (Scheme 4, eqn (a)). Coupling of 1a and 2a under the standard conditions in the presence of CD_3_OD afforded 3aa with 24% H/D exchange was detected at the *ortho*-position of the product 3aa, indicative of the irreversibility of the C–H activation process under the catalytic conditions (Scheme 4, eqn (a)). By employing deuterium-labeled compound [D_5_]-1a as substrate, we further determined the kinetic isotope effect (KIE) value of the parallel and intermolecular experiments to be 2.0 and 3.0 respectively, indicating that the cleavage of C–H bond at the *ortho*-position of *N*-phenyl-pyrazole might be involved in the rate-determining step (Scheme 4, eqn (b and c)).

**Scheme 5:**
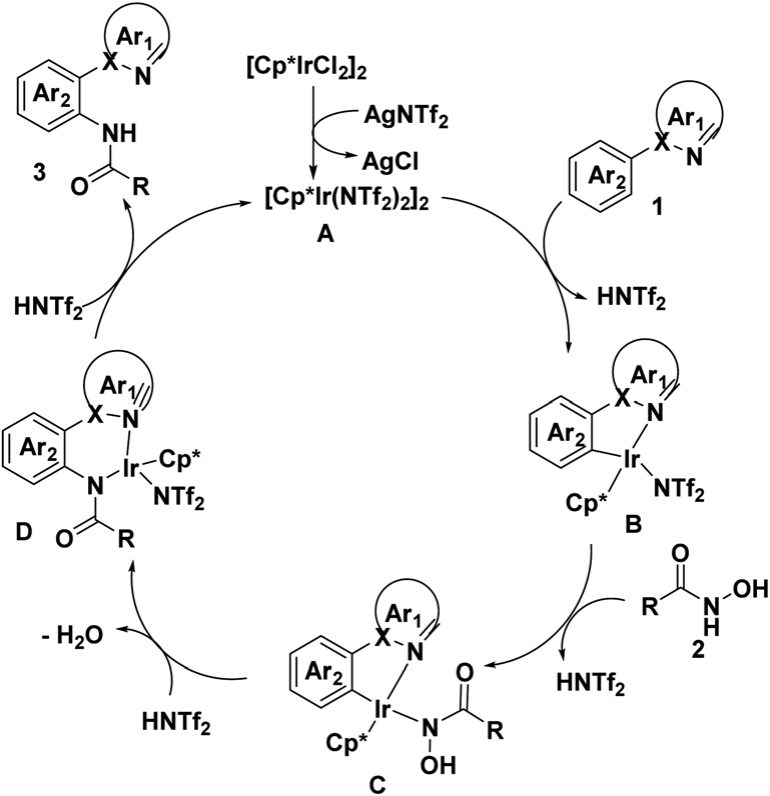
Plausible mechanism.

Based on the above results and literature precedence,^19^ a plausible mechanism is presented in Scheme 5. First, the treatment of [Cp*RhCl_2_]_2_ with AgNTf_2_ gives rise to the cationic Rh(iii) species A, which undergo C–H bond activation with 1 to generated HNTf_2_ and five-membered rhodacycle B, then coordinated to *N*-hydroxylamine 2 to afford intermediate C followed by migratory insertion, resulting in the formation of complex D and the release of H_2_O. Finally, proto-demetalation step lead to final product 3 and regenerated the Rh(iii)-catalyst.

## Conclusions

In summary, the combination of iridium catalyst and AgNtf_2_ additive deliver a highly efficient catalytic system for the C–H activation of *N*-aryl pyrazole with *N*-hydroxyamide, which allows the high efficient synthesis 2-amide substituted *N*-aryl pyrazole derivatives without any bisamidated products. This C–H bond amidation protocol is applicable to the coupling of a wide range of OH-free hydroxylamine, the reaction proceeds under oxidant-free or base-free conditions, enabling facile access to amidated products in good to high yields with a broad functional group tolerance. We believe that this strategy has potential applications in organic synthesis, as well as medicinal chemistry.

## Conflicts of interest

There are no conflicts to declare.

## Supplementary Material

RA-014-D4RA00517A-s001

RA-014-D4RA00517A-s002
