# Supplementary material for: Ir(iii)/Ag(i)-catalyzed directly C–H amidation of arenes with OH-free hydroxyamides as amidating agents
Source: RSC Adv. 2024 Feb 15;14(9):5975–80. doi: 10.1039/d4ra00517a (PMC10867557; doi:10.1039/d4ra00517a)

Electronic Supplementary Material (ESI) for RSC Advances.
This journal is © The Royal Society of Chemistry 2024

# Supporting Information

**Ir(III)/Ag(I)-Catalyzed Directly C-H Amidation of Arene with *OH*- Free Hydroxyamide as Amidating Agents**

Youpeng Zuo*, Meijun Liu, Jun Du, Tianren Zhang, Xiaoqing Wang, Cong Wang

*School of Chemistry and Chemical Engineering, Suzhou University, Suzhou 234000, P. R. China;*

Email: 812901107@qq.com

## Table of content

1. General Information------------------------------------------------------------------------------------S1
2. General Procedure for Synthesis of products 3-----------------------------------------------------S2
3. General Procedure for Synthesis of products 9aa-11am -----------------------------------------S3
4. General Procedure for Synthesis of products 3 from acyl azides-----------------------------S3-S4
5. Gram-Scale Synthesis of compound 3oa-------------------------------------------------------------S5
6. Mechanism study-----------------------------------------------------------------------------------S5-S9
7. References-----------------------------------------------------------------------------------------------S9
8. X-Ray Crystallography structures of Compound 3oa-------------------------------------------S10
9. Characterization Data--------------------------------------------------------------------------S12-S20
10. Copies of $^{1}$H and $^{13}$C NMR Spectra for all Compounds ----------------------------------S21-S58

## 1. General Information

Unless otherwise specified, all reagents and starting materials were purchased from commercial sources and used as received without purification. Starting materials *N*-aryl pyrazoles (**1**) [1], *N*-hydroxyamides (**2**) [2], 2-phenyl-4,5,6,7-tetrahydro-2H-indazole (**7**) [3], 2-phenyl-2H-indazole (**8**) [4] were synthesized using literature procedures. The solvents were purified and dried using standard procedures. All cascade reactions were performed in a resealable screw-capped Schleck flask in the presence of a Teflon-coated magnetic stirrer bar. The solvents were used directly without purification, unless stated. Thin-layer chromatography (TLC) was performed on percolated silica gel 60 F254 plates. Silica gel (200-300 mesh) was used for column chromatography. The $^{1}$H and $^{13}$C NMR spectra were recorded on a 500 MHz and 125 MHz NMR spectrometers, unless otherwise specified. Chemical shifts ($\delta$) in parts per million were reported relative to the residual signals of chloroform (7.26 ppm for $^{1}$H and 77.0 ppm for $^{13}$C), and all $^{13}$C NMR were recorded with proton broadband decoupling and indicated as $^{13}C\{^{1}H\}$ NMR. Multiplicities are described as s (singlet), d (doublet), t (triplet), q (quartet), or m (multiplet), and the coupling constants (*J*) are reported in Hertz (Hz). HRMS analysis with a quad- rupole time-of-flight mass spectrometer yielded ion mass/charge (m/z) ratios in atomic mass units.

## 2. General Procedure for Synthesis of products 3.

A mixture of *N*-aryl pyrazoles 1 (0.25 mmol), *N*-hydroxyamides (2) (0.25 mmol), $[Cp^*IrCl_2]_2$ (4 mol%), $AgNtf_2$ (20 mol%) were added under air atmosphere to a resealable screw-capped Schleck tube (25 mL). PhCl (3 mL) was then added. The tube was sealed with a Teflon-coated cap, and the resulting mixture was stirred in an oil bath preheated to 120 °C for 8 h (monitored by TLC). Upon completion of the reaction, the reaction mixture was cooled to room temperature, extracted with EtOAc (3 × 10 mL), and washed with brine. The organic layers were combined, dried over $Na_2SO_4$, filtered, and then evaporated under a vacuum. The residue was purified using flash column chromatography with silica gel (200-300 mesh), using ethyl acetate and petroleum ether as the elution solvent to give desired products 3.

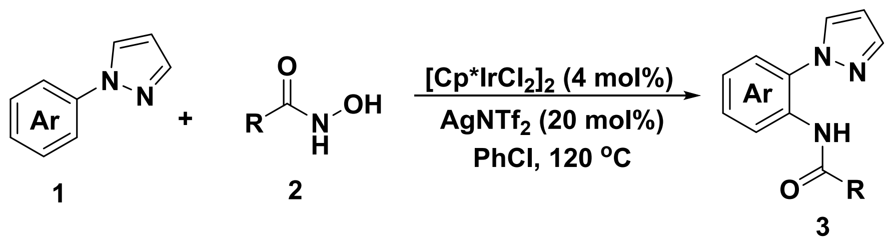

## 3. General Procedure for Synthesis of products 9aa-11am

A mixture of 2-phenylpyridine (**4a**)(0.25 mmol), *N*-hydroxybenzamide (**2a**) (0.25 mmol), $[Cp^{*}IrCl_2]_2$ (4 mol%), $AgNtf_2$ (20 mol%) were added under air atmosphere to a resealable screw-capped Schleck tube (25 mL). PhCl (3 mL) was then added. The tube was sealed with a Teflon-coated cap, and the resulting mixture was stirred in an oil bath preheated to 120 °C for 8 h (monitored by TLC). Upon completion of the reaction, the reaction mixture was cooled to room temperature, extracted with EtOAc (3 × 10 mL), and washed with brine. The organic layers were combined, dried over $Na_2SO_4$, filtered, and then evaporated under a vacuum. The residue was purified using flash column chromatography with silica gel (200-300 mesh), using ethyl acetate and petroleum ether as the elution solvent to give desired products **9aa**.

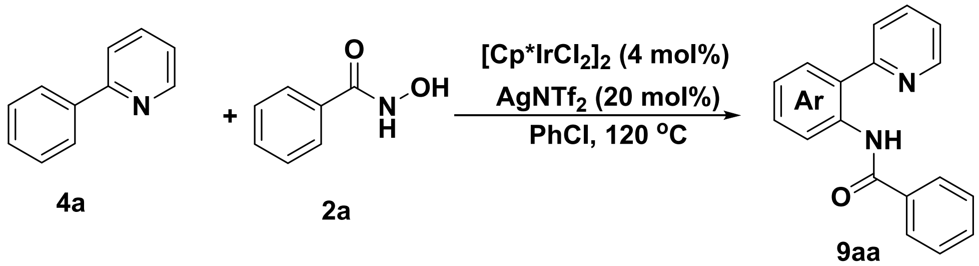

A mixture of 2-phenylpyrimidine (**5a**) (0.25 mmol), *N*-hydroxybenzamide (**2a**) (0.25 mmol), $[Cp^{*}IrCl_2]_2$ (4 mol%), $AgNtf_2$ (20 mol%) were added under air atmosphere to a resealable screw-capped Schleck tube (25 mL). PhCl (3 mL) was then added. The tube was sealed with a Teflon-coated cap, and the resulting mixture was stirred in an oil bath preheated to 120 °C for 8 h (monitored by TLC). Upon completion of the reaction, the reaction mixture was cooled to room temperature, extracted with EtOAc (3 × 10 mL), and washed with brine. The organic layers were combined, dried over $Na_2SO_4$, filtered, and then evaporated under a vacuum. The residue was purified using flash column chromatography with silica gel (200-300 mesh), using ethyl acetate and petroleum ether as the elution solvent to give desired products **10aa**.

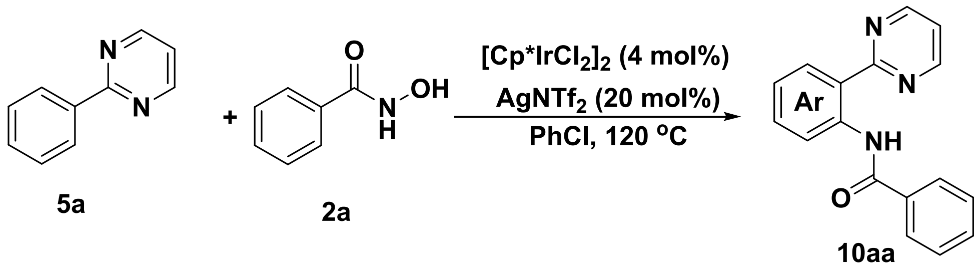


A mixture of 1-(pyrimidin-2-yl)-1*H*-indole (**6a**) (0.25 mmol), *N*-hydroxybenzamide (**2a**) or *4-bromo-N-hydroxybenzamide* (**2m**) (0.25 mmol), $[Cp^*IrCl_2]_2$ (4 mol%), $AgNtf_2$ (20 mol%) were added under air atmosphere to a resealable screw-capped Schleck tube (25 mL). PhCl (3 mL) was then added. The tube was sealed with a Teflon-coated cap, and the resulting mixture was stirred in an oil bath preheated to 120 °C for 8 h (monitored by TLC). Upon completion of the reaction, the reaction mixture was cooled to room temperature, extracted with EtOAc (3 × 10 mL), and washed with brine. The organic layers were combined, dried over $Na_2SO_4$, filtered, and then evaporated under a vacuum. The residue was purified using flash column chromatography with silica gel (200-300 mesh), using ethyl acetate and petroleum ether as the elution solvent to give desired products **11aa** and **11am**.

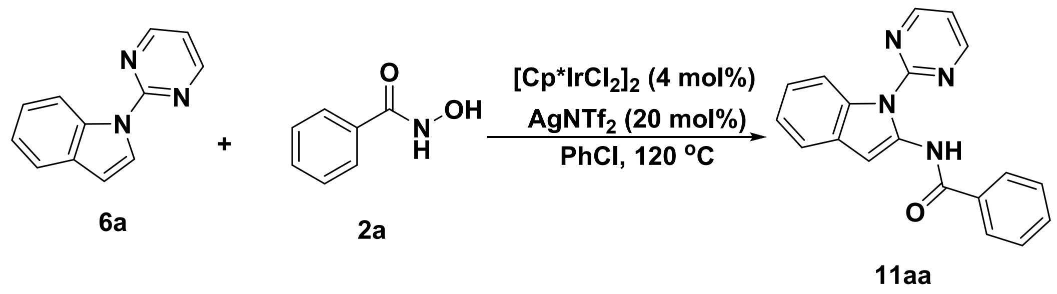


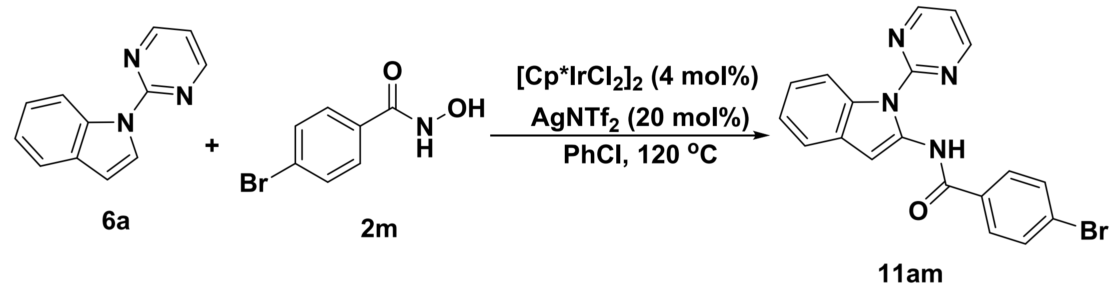


## 4. General Procedure for Synthesis of products 3 from acyl azides

A mixture of 1-aryl-1*H*-pyrazole 1 (0.25 mmol), acyl azides (14) (0.25 mmol), $[Cp^*IrCl_2]_2$ (4 mol%), $AgNtf_2$ (20 mol%) were added under air atmosphere to a resealable screw-capped Schleck tube. 1,2-DCE (3 mL) was then added. The tube was sealed with a Teflon-coated cap (25 mL), and the resulting mixture was stirred in an oil bath preheated to 60 °C for 8 h (monitored by TLC).

Upon completion of the reaction, the reaction mixture was cooled to room temperature, extracted with EtOAc (3 × 10 mL), and washed with brine. The organic layers were combined, dried over $Na_2SO_4$, filtered, and then evaporated under a vacuum. The residue was purified using flash column chromatography with silica gel (200-300 mesh), using ethyl acetate and petroleum ether as the elution solvent to give desired products 3.

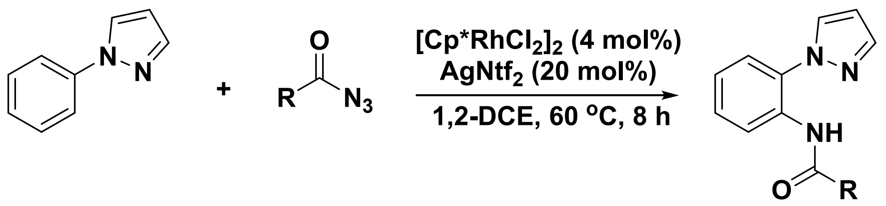


**5. Gram-Scale Synthesis of compound 3oa.**

Following the general procedures, the reaction of (1-(naphthalen-2-yl)-1*H*-pyrazole) (**1o**) (5 mmol) with *N*-hydroxybenzamide (**2a**) (5 mmol) under optimal condition afforded product **3oa** as a white solid in 88% yield (1.37 g).

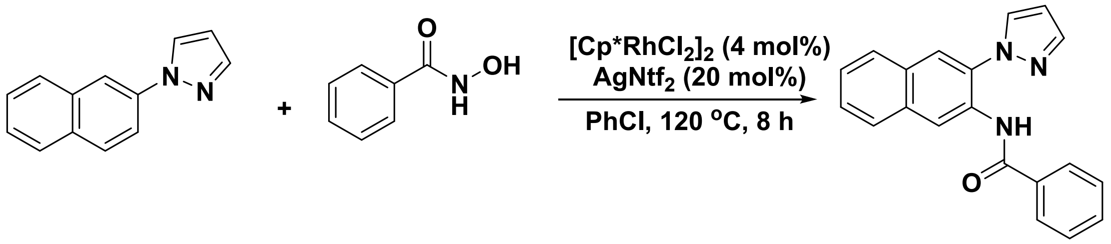


**6. Mechanism study**

a) H/D exchange studies

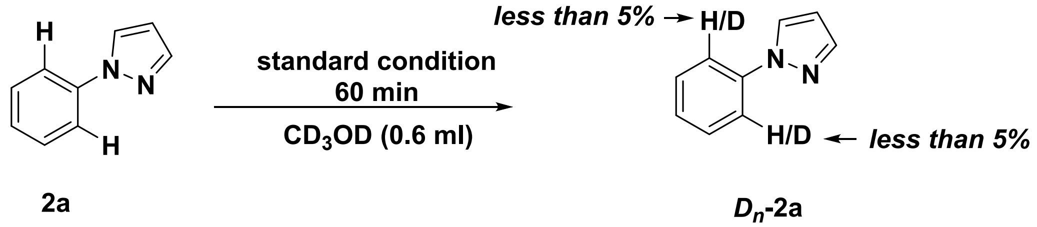


The substrate of **2a** (0.25 mmol) under standard condition and other $CD_3OD$ (0.6 mL) for 60 min. After that, the solvent was removed under reduced pressure and the residue was purified by silica gel chromatography using PE/EA to afford white solid, which was characterized by $^1H$ NMR spectroscopy.

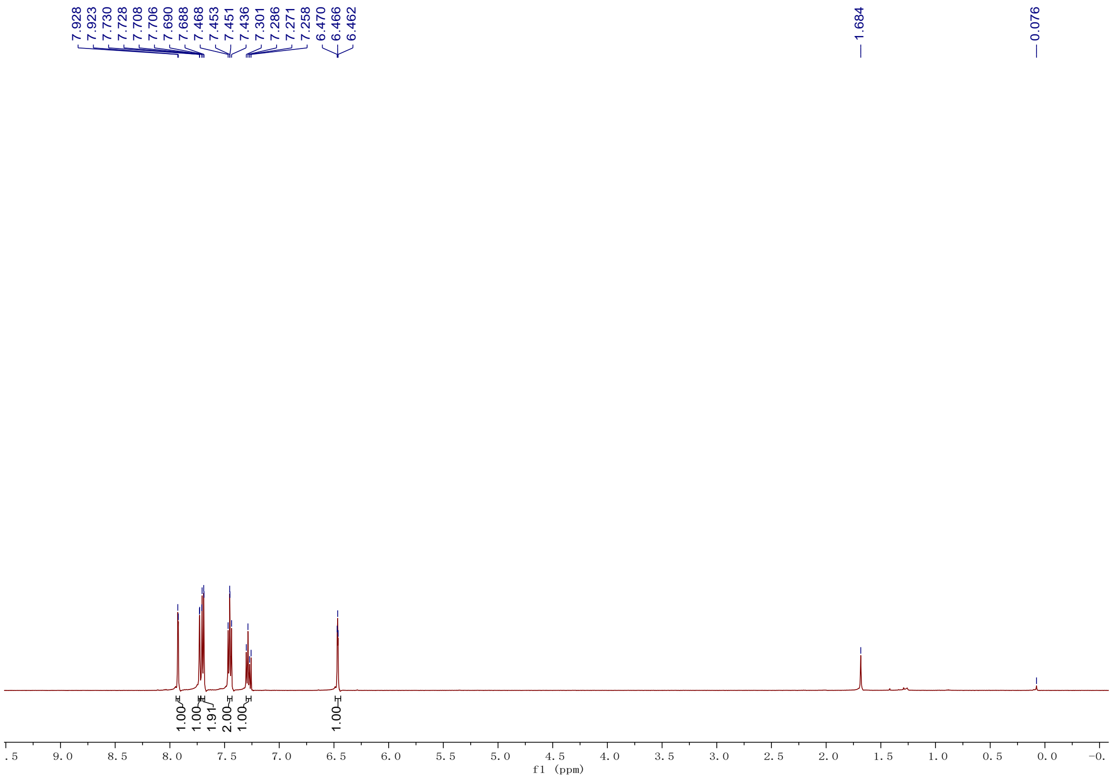


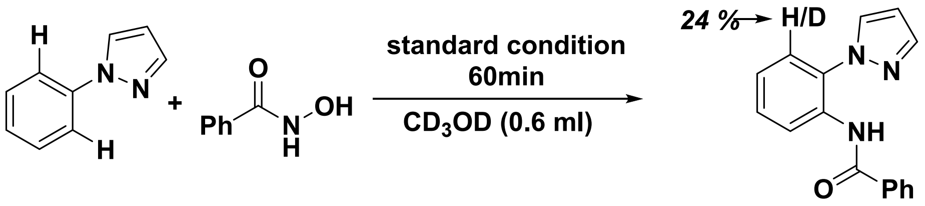


**1a** **2a** ***D*$_n$-3aa**

The mixture of 1a (0.25 mmol), 2a (0.25 mmol), and $CD_3OD$ (0.6 mL) under standard conditions for 60 min. After that, the solvent was removed under reduced pressure and the residue was purified by silica gel chromatography using PE/EA to afford white solid, which was characterized by $^1H$ NMR spectroscopy. $^1H$ NMR analysis of the coupled product 3aa revealed 24% deuteration at the *ortho*-position.

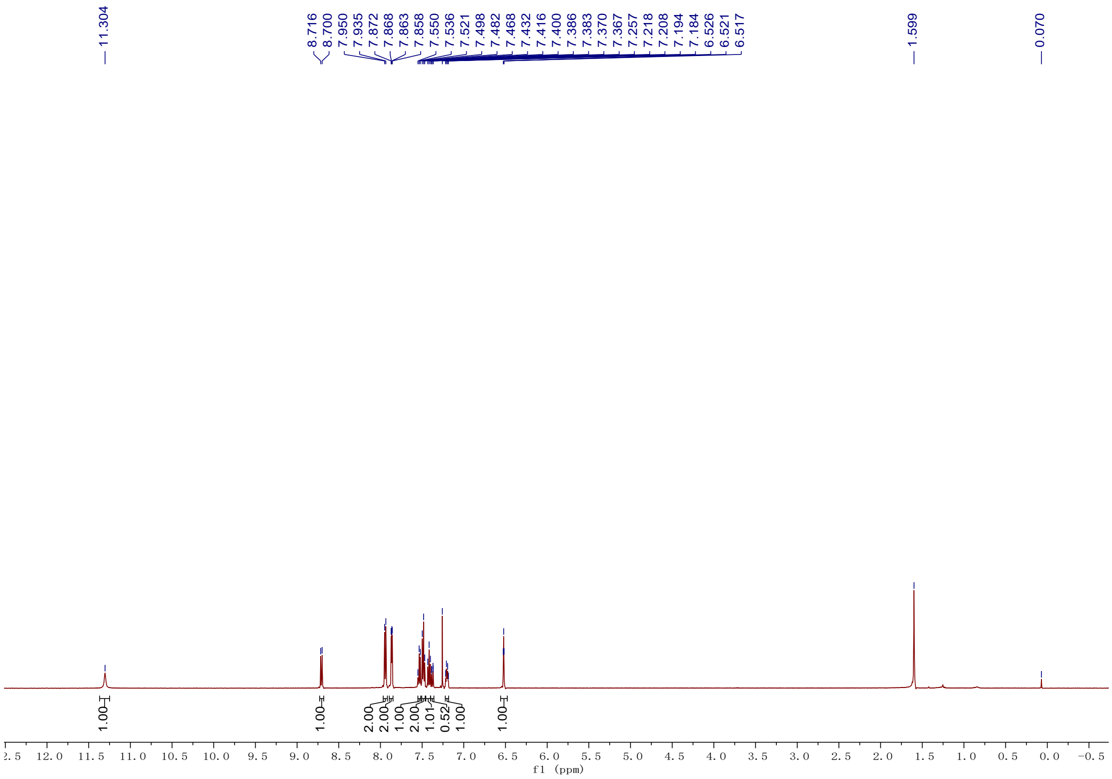


b) Parallel experiments

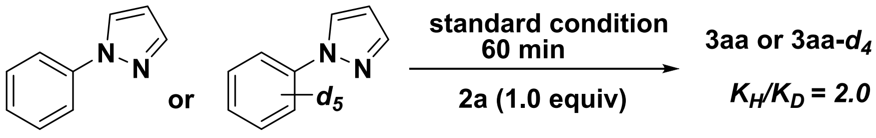


**1a** **1a-*d5***

The mixture of **1a** (0.25 mmol) or **1a-*d5*** (0.25 mmol), **2a** (0.25 mmol) were subjected to standard conditions for 60 min. After cooling to room temperature, both of reaction mixture were combined, the solvent was removed under reduced pressure and residue was purified by column chromatography (PE/EA) to afford the mixture of **3aa** and **3aa-*d4***. From the $^1$H NMR analysis the KIE was measured to be 2.0.

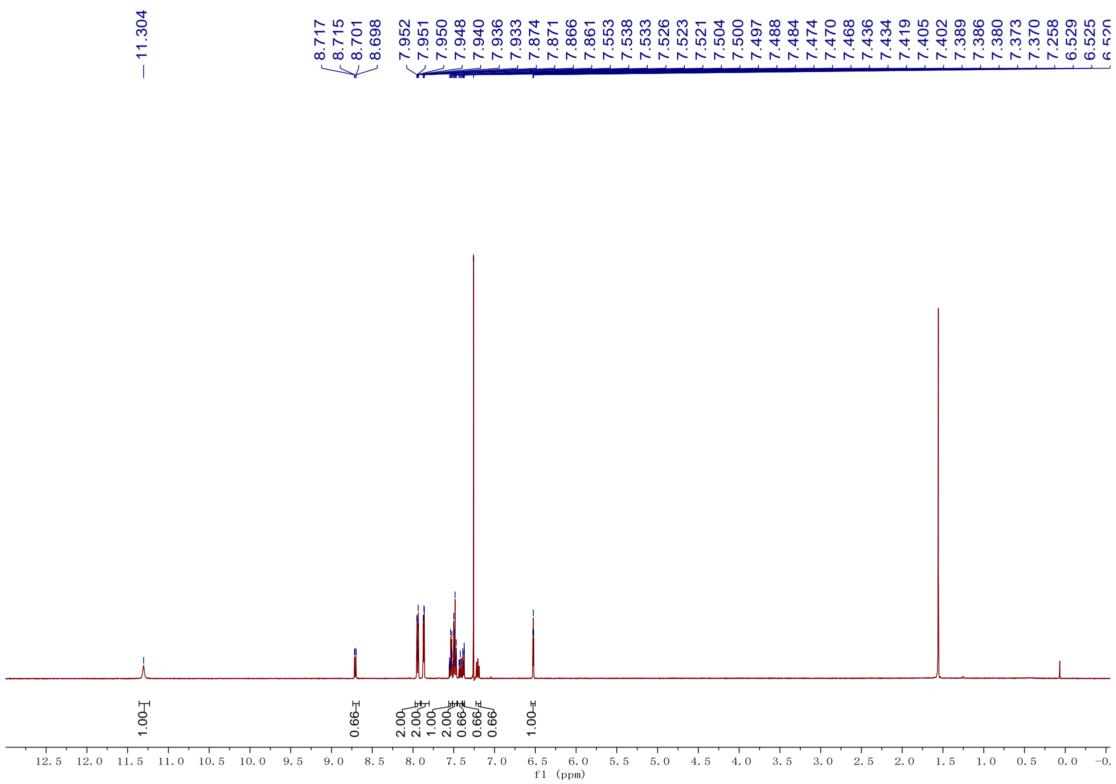

c) Competitive experiments

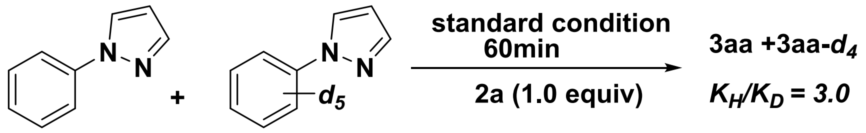


The mixture of **1a** (0.25 mmol) and **1a-*d5*** (0.25 mmol), **2a** (0.25 mmol) were subjected to standard conditions for 60 min. After cooling to room temperature, the solvent was removed under reduced pressure and residue was purified by column chromatography (PE/EA) to afford the mixture of **3aa** and **3aa-*d4***. From the $^{1}H$ NMR analysis the KIE was measured to be 3.0.

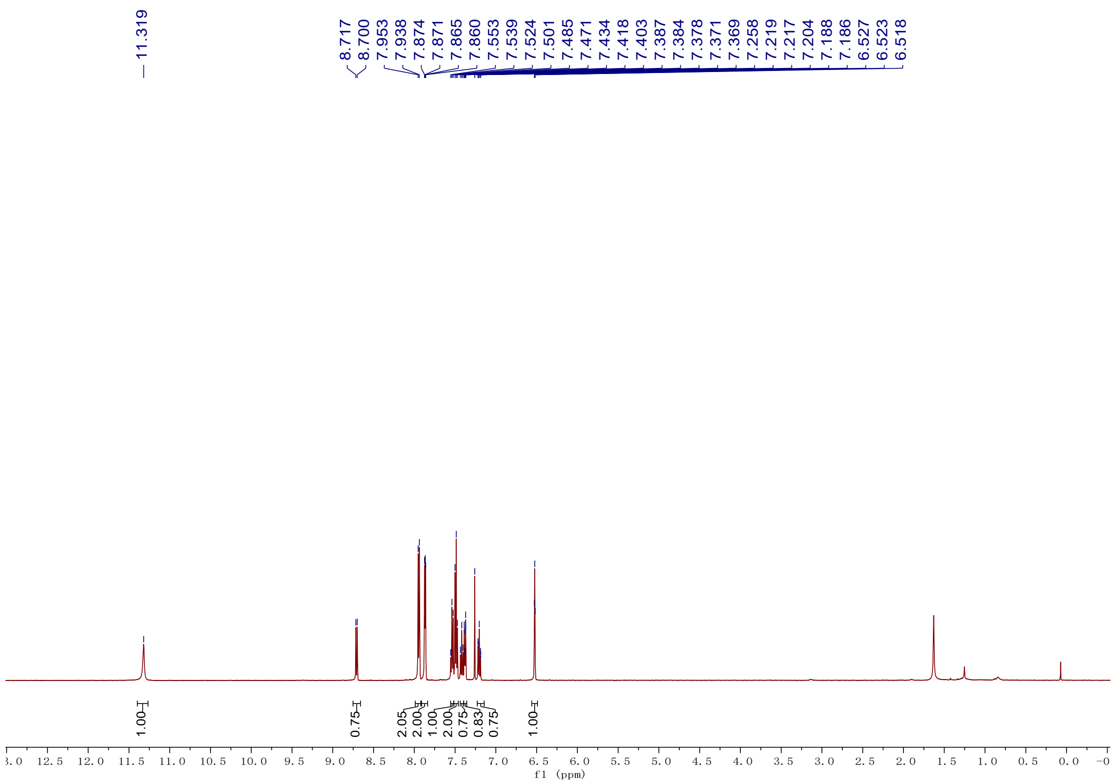

## 7. References


(1). (a) T. Nguyen, T.; V. Le, L.; H. Pham, H.; H. Nguyen, D.; T. S. Phan, N.; V. Leab, H.; N. Q. Phan, A. Cobalt-Catalyzed, Directed Arylation of C-H Bonds in *N*-aryl Pyrazoles. *RSC Adv.*, **2021**,*11*, 9349-9352. (b) B. Arockiam, P.; Fischmeister, C.; Bruneau, C.; H. Dixneuf, P. Ruthenium Diacetate-Catalysed Oxidative Alkenylation of C-H Bonds in Air: Synthesis of Alkenyl *N*-arylpyrazoles. *Green Chem.*, **2011**, *13*, 3075-3078. (c) Li, X.; Zhao, M. Rhodium(III)-Catalyzed Oxidative Coupling of 5-Aryl-1*H*-pyrazoles with Alkynes and Acrylates. *J. Org. Chem.* **2011**, *76*, 8530-8536.

(2). (a) Zuo, Y.; He, X.; Tang, Q.; Hu, W.; Zhou, T.; Shang, Y. Palladium-Catalyzed Cascade Decarboxylative Amination/6-endo-dig Benzannulation of *o*-Alkynylarylketones with N-Hydroxyamides To Access Diverse 1-Naphthylamine Derivatives. *Org. Lett.* **2020**, *22*, 3890-3894. (b) Zuo, Y.; He, X.; Tang, Q.; Hu, W.; Zhou, T.; Hu, W.; Shang, Y. Palladium-Catalyzed *5-exo-dig* Cyclization Cascade, Sequential Amination/Etherification for Stereoselective Construction of 3-Methyleneindolinones. *Advanced Synthesis & Catalysis*. **2021**, *363*, 2117-2123.

(3). (a) Modak, A.; J. Nett, A.; C. Swift, E.; C. Haibach, M.; S. Chan, V.; S. Franczyk, T.; Shekhar, S.; P. Cook, S. Cu-Catalyzed C-N Coupling with Sterically Hindered Partners. *ACS Catal.* **2020**, *10*, 10495-10499. (b) Liu, Y.; Wan, J. Tandem Reactions Initiated by Copper-Catalyzed Cross-

Coupling: A New Srategy Towards Heterocycle Synthesis. *Org. Biomol. Chem*., **2011**, *9*, 6873-6894.

(4). (a) Li, J.; Shi, L.; Zhang, S.; Wang, X.; Zhu, X.; Hao, X.; Song, M. Rh(III)-Catalyzed C-H Cyanation of 2*H*-Indazole with *N*-Cyano-*N*-phenyl-p-toluenesulfonamide. *J. Org. Chem*. **2020**, *85*, 10835-10845. (b) Dey, A.; Hajra, A. Iodine-Catalyzed Selenylation of 2*H*-Indazole. *J. Org. Chem*. **2019,** *84*, 14904-14910. (c) Murugan, A.; Babu, V. N.; Polu, A.; Sabarinathan, N.; Bakthadoss, M.; S. Sharada, D. Regioselective C3-H Trifluoromethylation of 2*H*-Indazole under Transition-Metal-Free Photoredox Catalysis. *J. Org. Chem*. **2019**, *84*, 7796-7803.

## 7. X-ray Crystallographic Data of Product 3oa

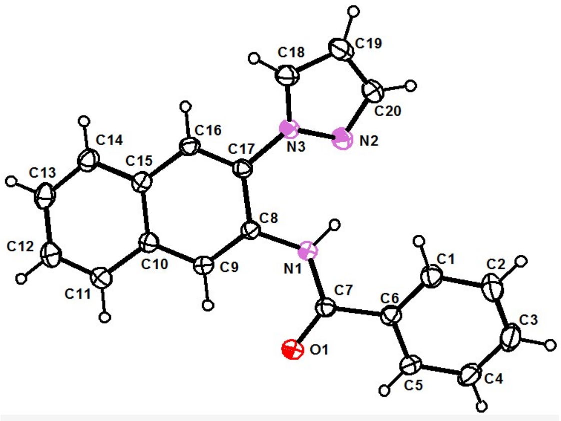


**Compound 3oa** was dissolved in the mix solvent of dichloromethane and hexane, a colorless bulk crystal was obtained after slowly evaporated. The X-ray crystalstructure determinations were obtained on a Bruker Smart CCDC APEX-2 diffractometer (graphite- monochromated Mo *Kα* radiation, λ=0.71073 nm) at 300 K.

**Figure S1**. ORTEP drawing of compound **3oa**. All hydrogen atoms have been omitted for clarity (CCDC 2315214) (30% probability for the thermal ellipsoid).

**Table S1.** Crystal data and structure refinement for **3oa.**

| | |
|---|---|
| Identification code | 3oa |
| Empirical formula | C20H15N3O |
| Formula weight | 313.35 |
| Temperature | 273(2) K |
| Wavelength | 0.71073 Å |
| Crystal system | Monoclinic |
| Space group | $P2_1/n$ |
| Unit cell dimensions | a = 8.0992(4) Å |
| α= 90°. | |
| b = 20.6903(11) Å | β= 111.728(2)°. |
| c = 9.9276(6) Å | γ = 90°. |
| Volume | 1545.42(15) $Å^3$ |
| Z | 4 |
| Density (calculated) | 1.347 $Mg/m^3$ |
| Absorption coefficient | 0.086 $mm^{-1}$ |
| F(000) | 656 |
| Crystal size | 8.099 x20.690 x 9.927 $mm^3$ |
| Theta range for data collection | 2.958 to 27.625°. |
| Index ranges | -10<=h<=10, -26<=k<=26, -12<=l<=12 |
| Reflections collected | 61845 |
| Independent reflections | 3540 [R(int) = 0.0228] |
| Completeness to theta = 25.242° | 98.6 % |
| Refinement method | Full-matrix least-squares on $F^2$ |
| Data / restraints / parameters | 3540/0/217 |
| Goodness-of-fit on $F^2$ | 1.078 |
| Final R indices [I>2sigma(I)] | R1 = 0.0505, wR2 = 0.1169 |
| R indices (all data) | R1 = 0.0565, wR2 = 0.1225 |
| Extinction coefficient | n/a |
| Largest diff. peak and hole | 0.250 and -0.221 e.$Å^{-3}$ |

***N*-(2-(1*H*-pyrazol-1-yl)phenyl)benzamide (3aa)**. This compound was purified by column chromatography (ethyl acetate/petroleum ether = 1:10) to afford a white solid in 86% yield (57 mg, 0.22 mmol); $^{1}$H NMR (500 MHz, $CDCl_3$) $\delta$ 11.23 (s,1H), 8.65 (d, $J$ = 8.5 Hz, 1H), 7.95 (d, $J$ = 7.0 Hz, 2H),7.88-7.86 (m, 2H), 7.54 (t, $J$ = 7.0 Hz, 1H), 7.48 (t, $J$ = 7.0 Hz, 2H), 7.43 (t, $J$ = 8.0 Hz, 1H), 7.39-7.37 (m, 1H), 7.23-7.20 (m, 1H), 6.53 (t, $J$ = 2.0 Hz, 1H); $^{13}$C NMR (125 MHz, $CDCl_3$) $\delta$ 165.7, 141.5, 135.2, 132.2, 130.7, 129.5, 129.0, 128.5, 127.6, 124.4, 123.3, 122.6, 107.7; HRMS (ESI-TOF) m/z: $[M + H]^+$ Calcd for $C_{16}H_{13}N_3O$ 264.1131; Found 264.1134.

***N*-(4-methyl-2-(1*H*-pyrazol-1-yl)phenyl)benzamide (3ba).** This compound was purified by column chromatography (ethyl acetate/petroleum ether = 1:10) to afford a white solid in 82% yield (57 mg, 0.21 mmol); $^{1}$H NMR (500 MHz, $CDCl_3$) $\delta$ 11.10 (s, 1H), 8.51 (d, $J$ = 8.5 Hz, 1H), 7.93 (d, $J$ = 7.0 Hz, 2H), 7.86-7.85 (m, 2H), 7.52 (t, $J$ = 7.0 Hz, 1H ), 7.48-7.46 (m, 2H), 6.51 (t, $J$ = 2.0 Hz, 1H), 2.40 (s, 3H); $^{13}$C NMR (125 MHz, $CDCl_3$) $\delta$ 165.5, 141.4, 135.2, 134.4, 132.1, 130.6, 129.6, 129.4, 129.0, 127.6, 123.3, 123.1, 107.5, 21.2; HRMS (ESI-TOF) m/z: $[M + H]^+$ Calcd for $C_{17}H_{15}N_3O$ 278.1288; Found 278.1287.

***N*-(5-methyl-2-(1*H*-pyrazol-1-yl)phenyl)benzamide (3ca).** This compound was purified by column chromatography (ethyl acetate/petroleum ether = 1:10) to afford a white solid in 86% yield (60 mg, 0.22 mmol); $^{1}$H NMR (500 MHz, $CDCl_3$) $\delta$ 11.15 (s, 1H), 8.49 (s, 1H), 7.93 (d, $J$ = 8.5 Hz, 2H), 7.86-7.82 (m, 2H), 7.53 (t, $J$ = 7.0 Hz, 1H), 7.48 (t, $J$ = 7.5 Hz, 2H), 7.26-7.24 (m, 1H), 7.01 (d, $J$ = 8.0 Hz, 1H), 6.50 (s, 1H), 2.44 (s, 1H); $^{13}$C NMR (125 MHz, $CDCl_3$) $\delta$ 165.6, 141.3, 138.7, 135.2, 132.1, 130.6, 129.0, 127.6, 127.3, 125.1, 123.6, 122.4, 107.4, 21.8; HRMS (ESI-TOF) m/z: $[M + H]^+$ Calcd for $C_{17}H_{15}N_3O$ 278.1288; Found 278.1291.

***N*-(5-methoxy-2-(1*H*-pyrazol-1-yl)phenyl)benzamide (3da).** This compound was purified by column chromatography (ethyl acetate/petroleum ether = 1:10) to afford a white solid in 88% yield (64 mg, 0.22 mmol); $^{1}$H NMR (500 MHz, $CDCl_3$) $\delta$ 11.09 (s, 1H), 7.94-7.92 (m, 2H), 7.87-7.84 (m, 1H), 7.80-7.77 (m, 1H), 7.53 (t, $J$ = 7.0 Hz, 1H), 7.48 (t, $J$ = 7.0 Hz, 2H), 7.27 (d, $J$ = 9.0 Hz, 1H), 6.76-6.73 (m, 1H), 6.50 (t, $J$ = 2.0 Hz, 1H), 3.90 (s, 3H); $^{13}$C NMR (125 MHz, $CDCl_3$) $\delta$ 165.7, 159.5, 141.2, 135.1, 133.5, 132.2, 130.6, 129.1, 127.6, 123.6, 123.0, 110.8, 107.4, 107.2, 56.1; HRMS (ESI-TOF) m/z: $[M + H]^+$ Calcd for $C_{17}H_{15}N_3O_2$ 294.1237; Found 294.1239.

***N*-(5-ethyl-2-(1*H*-pyrazol-1-yl)phenyl)benzamide (3ea).** This compound was purified by column chromatography (ethyl acetate/petroleum ether = 1:10) to afford a white solid in 88% yield (64 mg, 0.22 mmol); $^{1}$H NMR (500 MHz, $CDCl_3$) $\delta$ 11.22 (s, 1H), 8.57 (s, 1H), 7.94-7.92 (m,

2H), 7.85-7.82 (m, 2H), 7.53 (t, $J$ = 7.5 Hz, 1H), 7.48 (t, $J$ = 7.5 Hz, 2H), 7.28 (d, $J$ = 8.0 Hz, 1H), 7.03 (d, $J$ = 8.0 Hz, 1H), 6.50 (t, $J$ = 2.0 Hz, 1H), 2.74 (q, $J$ = 7.5 Hz, 2H), 1.30 (t, $J$ = 7.5 Hz, 3H); $^{13}$C NMR (125 MHz, $CDCl_3$) $\delta$ 165.6, 145.0, 141.3, 135.2, 132.1, 132.0, 129.0, 127.6, 127.4, 123.8, 122.5, 122.5, 107.4, 29.1, 15.8; HRMS (ESI-TOF) m/z: $[M + H]^+$ Calcd for $C_{18}H_{17}N_3O$ 292.1444; Found 292.1439.

***N*-(4-(1*H*-pyrazol-1-yl)-[1,1'-biphenyl]-3-yl)benzamide (3fa).** This compound was purified by column chromatography (ethyl acetate/petroleum ether = 1:10) to afford a white solid in 88% yield (64 mg, 0.22 mmol); $^1$H NMR (500 MHz, $CDCl_3$) $\delta$ 11.44 (s, 1H), 9.04 (s, 1H), 7.98-7.96 (m, 2H), 7.91-7.86 (m, 2H), 7.72-7.70 (m, 2H), 7.55 (t, $J$ = 7.5 Hz, 1H), 7.51-7.44 (m, 6H), 7.37 (t, $J$ = 7.5 Hz, 1H), 6.54 (t, $J$ = 2.0 Hz, 1H); $^{13}$C NMR (125 MHz, $CDCl_3$) $\delta$ 165.8, 141.5, 141.3, 140.2, 135.2, 132.4, 132.2, 130.5, 129.2, 129.1, 128.5, 128.1, 127.7, 127.6, 122.8, 122.7, 121.8, 107.7; HRMS (ESI-TOF) m/z: $[M + H]^+$ Calcd for $C_{22}H_{17}N_3O$ 340.1444; Found 340.1440.

***N*-(4-chloro-2-(1*H*-pyrazol-1-yl)phenyl)benzamide (3ga).** This compound was purified by column chromatography (ethyl acetate/petroleum ether = 1:10) to afford a white solid in 83% yield (62 mg, 0.21 mmol); $^1$H NMR (500 MHz, $CDCl_3$) $\delta$ 11.35 (s, 1H), 8.69 (d, $J$ = 9.5 Hz, 1H), 7.93 (d, $J$ = 7.5 Hz, 2H), 7.78 (d, $J$ = 6.0 Hz, 2H), 7.54 (t, $J$ = 7.5 Hz, 1H), 7.48 (t, $J$ = 7.5 Hz, 2H), 7.38-7.36 (m, 2H), 6.54 (t, $J$ = 2.0 Hz, 1H); $^{13}$C NMR (125 MHz, $CDCl_3$) $\delta$ 165.7, 141.9, 134.9, 132.3, 130.8, 130.6, 130.0, 129.1, 128.2, 127.6, 124.4, 122.4, 108.1; HRMS (ESI-TOF) m/z: $[M + H]^+$ Calcd for $C_{16}H_{12}ClN_3O$ 298.0742; Found 298.0744.

***N*-(4-bromo-2-(1*H*-pyrazol-1-yl)phenyl)benzamide (3ha).** This compound was purified by column chromatography (ethyl acetate/petroleum ether = 1:10) to afford a white solid in 84% yield (72 mg, 0.21 mmol); $^1$H NMR (500 MHz, $CDCl_3$) $\delta$ 11.35 (s, 1H), 8.60 (d, $J$ = 9.5 Hz, 1H), 7.93 (d, $J$ = 7.5 Hz, 1H), 7.88-7.86 (m, 2H), 7.56-7.47 (m, 5H), 6.54 (t, $J$ = 2.0 Hz, 1H); $^{13}$C NMR (125 MHz, $CDCl_3$) $\delta$ 165.7, 141.9, 134.9, 132.3,131.3, 131.1, 130.6, 130.2, 129.1, 127.6, 125.2, 124.6, 116.3, 108.1; HRMS (ESI-TOF) m/z: $[M + H]^+$ Calcd for $C_{16}H_{12}BrN_3O$ 342.0237; Found 342.0236.

***N*-(5-fluoro-2-(1*H*-pyrazol-1-yl)phenyl)benzamide (3ia).** This compound was purified by column chromatography (ethyl acetate/petroleum ether = 1:10) to afford a white solid in 90% yield (63 mg, 0.23 mmol); $^1$H NMR (500 MHz, $CDCl_3$) $\delta$ 11.31 (s, 1H), 8.57-8.54 (m, 1H), 7.93-7.91 (m, 2H), 7.88-7.87 (m, 1H), 7.82-7.81 (m, 1H), 7.55 (t, $J$ = 7.5 Hz, 1H), 7.49 (t, $J$ = 7.5 Hz, 2H), 7.34-7.31 (m, 1H), 6.91-6.88 (m, 1H), 6.53 (t, $J$ = 2.0 Hz, 1H); $^{13}$C NMR (125 MHz, $CDCl_3$)

$\delta$ 165.7, 160.0 (d, $J_{C\text{-}F}$ = 244.3 Hz), 141.6, 133.8 (d, $J_{C\text{-}F}$ = 12.0 Hz), 132.4, 130.8, 129.1, 127.6, 125.6 (d, $J_{C\text{-}F}$ = 2.7 Hz), 123.7 (d, $J_{C\text{-}F}$ = 9.7 Hz), 110.9 (d, $J_{C\text{-}F}$ = 23.3 Hz), 110.2 (d, $J_{C\text{-}F}$ = 28.6 Hz), 107.8; HRMS (ESI-TOF) m/z: $[M + H]^+$ Calcd for $C_{16}H_{12}FN_3O$ 282.1037; Found 282.1038.

***N*-(5-chloro-2-(1*H*-pyrazol-1-yl)phenyl)benzamide (3ja)**. This compound was purified by column chromatography (ethyl acetate/petroleum ether = 1:10) to afford a white solid in 92% yield (66 mg, 0.22 mmol); $^1$H NMR (500 MHz, $CDCl_3$) $\delta$ 11.41 (s, 1H), 8.83 (s, 1H), 7.93 (d, $J$ = 7.0 Hz, 2H), 7.89-7.87 (m, 2H), 7.85-7.83 (m, 2H), 7.55 (t, $J$ = 7.5 Hz, 1H), 7.49 (t, $J$ = 7.5 Hz, 2H), 7.30 (d, $J$ = 8.5 Hz, 1H), 7.17-7.15 (m, 1H), 6.54 (t, $J$ = 2.0 Hz, 1H); $^{13}$C NMR (125 MHz, $CDCl_3$) $\delta$ 165.7, 141.7, 134.8, 134.0, 133.1, 132.4, 130.6, 129.1, 127.7, 127.6, 124.4, 123.2, 122.9, 107.9; HRMS (ESI-TOF) m/z: $[M + H]^+$ Calcd for $C_{16}H_{12}ClN_3O$ 298.0742; Found 298.0741.

***N*-(5-bromo-2-(1*H*-pyrazol-1-yl)phenyl)benzamide (3ka).** This compound was purified by column chromatography (ethyl acetate/petroleum ether = 1:10) to afford a white solid in 92% yield (78 mg, 0.23 mmol); $^1$H NMR (500 MHz, $CDCl_3$) $\delta$ 11.44 (s, 1H), 8.97 (s, 1H), 7.93 (d, $J$ = 7.0 Hz, 2H), 7.88-7.87 (m, 1H), 7.85-7.83 (m, 1H), 7.55 (t, $J$ = 7.5 Hz, 1H), 7.50 (t, $J$ = 7.5 Hz, 2H), 7.32-7.30 (m, 1H), 7.23 (t, $J$ = 7.5 Hz, 1H), 6.53 (t, $J$ = 2.0 Hz, 1H); $^{13}$C NMR (125 MHz, $CDCl_3$) $\delta$ 165.7, 141.7, 134.8, 133.2, 132.4, 130.5, 129.1, 128.1, 127.6, 125.8, 123.4, 121.8, 108.0; HRMS (ESI-TOF) m/z: $[M + H]^+$ Calcd for $C_{16}H_{12}BrN_3O$ 342.0237; Found 342.0237.

***N*-(5-iodo-2-(1*H*-pyrazol-1-yl)phenyl)benzamide (3la).** This compound was purified by column chromatography (ethyl acetate/petroleum ether = 1:10) to afford a white solid in 87% yield (84 mg, 0.22 mmol); $^1$H NMR (500 MHz, $CDCl_3$) $\delta$ 11.39 (s,1H), 9.12 (s, 1H), 7.93 (d, $J$ = 7.5 Hz, 2H) 7.88-7.87 (m, 1H), 7.85-7.83 (m, 1H), 7.56-7.47 (m, 4H), 7.09 (d, $J$ = 8.0 Hz, 1H), 6.53 (t, $J$ = 2.0 Hz, 1H); $^{13}$C NMR (125 MHz, $CDCl_3$) $\delta$ 165.6, 141.7, 134.8, 133.3, 133.1, 132.4, 131.7, 130.5, 129.1, 128.9, 127.6, 123.6, 108.0, 92.8; HRMS (ESI-TOF) m/z: $[M + H]^+$ Calcd for $C_{16}H_{12}IN_3O$ 390.0098; Found 390.0097.

***N*-(2-(1*H*-pyrazol-1-yl)-5-(trifluoromethyl)phenyl)benzamide (3ma).** This compound was purified by column chromatography (ethyl acetate/petroleum ether = 1:10) to afford a white solid in 84% yield (69 mg, 0.21 mmol); $^1$H NMR (500 MHz, $CDCl_3$) $\delta$ 11.71 (m, 1H), 9.14 (m, 1H), 7.96 (d, $J$ = 7.5 Hz, 2H), 7.93-7.91 (m, 2H), 7.56 (t, $J$ = 7.5 Hz, 1H), 7.52-7.48 (m, 3H), 7.44 (d, $J$ = 8.5 Hz, 1H), 6.57 (t, $J$ = 2.0 Hz, 1H); $^{13}$C NMR (125 MHz, $CDCl_3$) $\delta$ 165.9, 142.1, 134.7, 132.5, 132.5, 131.2, 130.6, 130.3 (q, $J_{C\text{-}F}$ = 32.8 Hz), 129.1, 127.7, 124.0 (q, $J_{C\text{-}F}$ = 270.8 Hz), 121.0 (q, $J_{C\text{-}F}$ = 3.8 Hz), 120.4 (q, $J_{C\text{-}F}$ = 3.8 Hz), 108.3; HRMS (ESI-TOF) m/z: $[M + H]^+$ Calcd for

$C_{17}H_{12}F_3N_3O$ 332.1005; Found 332.1002.

***N*-(5-cyano-2-(1*H*-pyrazol-1-yl)phenyl)benzamide (3na).** This compound was purified by column chromatography (ethyl acetate/petroleum ether = 1:10) to afford a white solid in 83% yield (60 mg, 0.21 mmol); $^1$H NMR (500 MHz, $CDCl_3$) $\delta$ 11.83 (s, 1H), 9.20 (s, 1H), 7.97-7.93 (m, 4H), 7.57 (t, $J$ = 7.5 Hz, 1H), 7.51 (t, $J$ = 7.5 Hz, 1H), 7.48 (s, 2H), 6.60 (t, $J$ = 2.0 Hz, 1H); $^{13}$C NMR (125 MHz, $CDCl_3$) $\delta$ 165.9, 142.4, 134.5, 132.7, 132.6, 131.6, 130.6, 129.2, 127.7, 126.8, 122.4, 118.4, 111.9, 108.6; HRMS (ESI-TOF) m/z: $[M + H]^+$ Calcd for $C_{17}H_{12}N_4O$ 289.1084; Found 289.1081.

***N*-(3-(1*H*-pyrazol-1-yl)naphthalen-2-yl)benzamide (3oa).** This compound was purified by column chromatography (ethyl acetate/petroleum ether = 1:10) to afford a white solid in 94% yield (74 mg, 0.23 mmol); $^1$H NMR (500 MHz, $CDCl_3$) $\delta$ 11.29 (s, 1H), 9.20 (s, 1H), 7.99-7.96 (m, 3H), 7.94-7.92 (m, 2H), 7.82 (s, 1H), 7.80 (d, $J$ = 7.5 Hz, 1H), 7.56-7.45 (m, 5H), 6.58 (t, $J$ = 2.0 Hz, 1H); $^{13}$C NMR (125 MHz, $CDCl_3$) $\delta$ 165.8, 140.2, 135.2, 133.2, 132.2, 131.4, 130.2, 129.9, 129.6, 129.1, 128.3, 127.6, 127.6, 127.4, 126.4, 121.2, 120.4, 107.9; HRMS (ESI-TOF) m/z: $[M + H]^+$ Calcd for $C_{20}H_{15}N_3O$ 314.1288; Found 314.1286.

***N*-(2-(1*H*-pyrazol-1-yl)phenyl)-2-hydroxybenzamide (3ab).** This compound was purified by column chromatography (ethyl acetate/petroleum ether = 1:10) to afford a white solid in 94% yield (63 mg, 0.23 mmol); $^1$H NMR (500 MHz, $CDCl_3$) $\delta$ 12.22 (s, 1H), 11.62 (s, 1H), 8.54 (d, $J$ = 8.0 Hz, 1H), 7.91-7.88 (m, 2H), 7.65 (d, $J$ = 8.5 Hz, 1H), 7.44-7.39 (m, 3H), 7.00 (d, $J$ = 8.5 Hz, 1H), 6.93 (t, $J$ = 7.5 Hz, 1H), 6.54 (t, $J$ = 2.0 Hz, 1H); $^{13}$C NMR (125 MHz, $CDCl_3$) $\delta$ 169.1, 162.5, 141.6, 134.8, 131.0, 130.5, 129.8, 128.2, 126.4, 125.1, 123.9, 122.4, 119.3, 119.0, 115.3, 107.8; HRMS (ESI-TOF) m/z: $[M + H]^+$ Calcd for $C_{16}H_{13}N_3O_2$ 280.1081; Found 280.1083.

***N*-(2-(1*H*-pyrazol-1-yl)phenyl)-3-methylbenzamide (3ac).** This compound was purified by column chromatography (ethyl acetate/petroleum ether = 1:10) to afford a white solid in 81% yield (56 mg, 0.20 mmol); $^1$H NMR (500 MHz, $CDCl_3$) $\delta$ 11.20 (s, 1H), 8.68 (d, $J$ = 8.5 Hz, 1H), 7.87-7.85 (m, 2H), 7.76 (s, 1H), 7.77 (d, $J$ = 7.0Hz, 1H), 7.42 (t, $J$ = 8.5 Hz, 1H), 7.38-7.33 (m, 3H), 7.20 (t, $J$ = 8.0 Hz, 1H), 6.53 (t, $J$ = 2.0 Hz, 1H), 2.43 (s, 3H); $^{13}$C NMR (125 MHz, $CDCl_3$) $\delta$ 165.9, 141.4, 138.8, 135.2, 132.9, 132.3, 130.6, 129.5, 128.9, 128.5, 124.6, 124.3, 123.2, 122.6, 107.6, 21.8; HRMS (ESI-TOF) m/z: $[M + H]^+$ Calcd for $C_{17}H_{15}N_3O$ 278.1288; Found 278.1283.

***N*-(2-(1*H*-pyrazol-1-yl)phenyl)-4-methylbenzamide (3ad).** This compound was purified by column chromatography (ethyl acetate/petroleum ether = 1:10) to afford a white solid in 83%

yield (57 mg, 0.21 mmol); $^1$H NMR (500 MHz, $CDCl_3$) $\delta$ 11.21 (s, 1H), 8.70 (d, $J$ = 8.5 Hz, 1H), 7.86-7.82 (m, 4H), 7.41 (t, $J$ = 7.5 Hz, 1H), 7.36 (d, $J$ = 8.0 Hz, 1H), 7.28 (d, $J$ = 8.0 Hz, 2H), 7.18 (t, $J$ = 8.0 Hz, 1H), 6.51 (t, $J$ = 2.0 Hz, 1H), 2.41 (s, 3H); $^{13}$C NMR (125 MHz, $CDCl_3$) $\delta$ 165.7, 142.6, 141.4, 132.4, 132.3, 130.6, 129.7, 129.4, 128.4, 127.7, 124.2, 123.3, 122.6, 107.6, 21.8; HRMS (ESI-TOF) m/z: $[M + H]^+$ Calcd for $C_{17}H_{15}N_3O$ 278.1288; Found 278.1286.

***N*-(2-(1*H*-pyrazol-1-yl)phenyl)-4-methoxybenzamide (3ae).** This compound was purified by column chromatography (ethyl acetate/petroleum ether = 1:10) to afford a white solid in 84% yield (61 mg, 0.21 mmol); $^1$H NMR (500 MHz, $CDCl_3$) $\delta$ 11.13 (s, 1H), 8.66 (d, $J$ = 9.0 Hz, 1H), 7.91 (d, $J$ = 8.5 Hz, 1H), 7.88-7.85 (m, 2H), 7.41 (t, $J$ = 8.0 Hz, 1H), 7.37 (d, $J$ = 8.0 Hz, 1H), 7.20-7.17 (m, 1H), 6.97 (d, $J$ = 8.5 Hz, 1H), 6.52 (t, $J$ = 2.0 Hz, 1H), 3.87 (s, 3H); $^{13}$C NMR (125 MHz, $CDCl_3$) $\delta$ 165.3, 162.8, 141.4, 132.4, 130.7, 129.5, 129.4, 128.4, 127.5, 124.1, 123.2, 122.6, 114.2, 107.6, 55.8; HRMS (ESI-TOF) m/z: $[M + H]^+$ Calcd for $C_{17}H_{15}N_3O_2$ 294.1237; Found 294.1234.

***N*-(2-(1*H*-pyrazol-1-yl)phenyl)-4-(tert-butyl)benzamide (3af).** This compound was purified by column chromatography (ethyl acetate/petroleum ether = 1:10) to afford a white solid in 84% yield (67 mg, 0.21 mmol); $^1$H NMR (500 MHz, $CDCl_3$) $\delta$ 11.23 (s, 1H), 8.72 (d, $J$ = 8.0 Hz, 1H), 7.89-7.84 (m, 4H), 7.50 (d, $J$ = 8.0 Hz, 2H), 7.40 (t, $J$ = 7.5 Hz, 1H), 7.36 (d, $J$ = 8.0 Hz, 1H), 7.18 (t, $J$ = 7.5 Hz, 1H), 6.51 (t, $J$ = 2.0 Hz, 1H); 1.35 (s, 9H); $^{13}$C NMR (125 MHz, $CDCl_3$) $\delta$ 165.6, 155.7, 141.5, 132.4, 132.3, 130.6, 129.5, 128.4, 127.5, 126.0, 124.2, 123.3, 122.6, 107.6, 35.3, 31.5; HRMS (ESI-TOF) m/z: $[M + H]^+$ Calcd for $C_{20}H_{21}N_3O$ 320.0540; Found 320.0539.

***N*-(2-(1*H*-pyrazol-1-yl)phenyl)-2-bromobenzamide (3ag).** This compound was purified by column chromatography (ethyl acetate/petroleum ether = 1:10) to afford a white solid in 95% yield (81 mg, 0.24 mmol); $^1$H NMR (500 MHz, $CDCl_3$) $\delta$ 10.65 (s, 1H), 8.67 (d, $J$ = 8.0 Hz, 1H), 7.83-7.82 (m, 1H), 7.71-7.67 (m, 1H), 7.61 (d, $J$ = 8.0 Hz, 1H), 7.54 (d, $J$ = 8.0 Hz, 1H), 7.42 (t, $J$ = 7.5 Hz, 1H), 7.39-7.35 (m, 2H), 7.29 (t, $J$ = 7.5 Hz, 1H), 7.22 (t, $J$ = 8.0 Hz, 1H), 6.47 (t, $J$ = 2.0 Hz, 1H); $^{13}$C NMR (125 MHz, $CDCl_3$) $\delta$ 166.2, 141.7, 138.5, 134.0, 131.8, 131.7, 130.6, 129.8, 129.6, 128.5, 127.9, 124.9, 123.5, 122.9, 120.0, 107.6; HRMS (ESI-TOF) m/z: $[M + H]^+$ Calcd for $C_{16}H_{12}BrN_3O$ 342.0237; Found 342.0234.

***N*-(2-(1*H*-pyrazol-1-yl)phenyl)-4-bromo-2-methylbenzamide (3ah).** This compound was purified by column chromatography (ethyl acetate/petroleum ether = 1:10) to afford a white solid in 80 % yield (71 mg, 0.20 mmol); $^1$H NMR (500 MHz, $CDCl_3$) $\delta$ 10.75 (s, 1H), 8.65 (d, $J$ = 9.0

Hz, 1H), 7.85-7.83 (m, 1H), 7.73-7.71 (m, 1H), 7.43-7.34 (m, 5H), 7.21 (t, $J$ = 7.5 Hz, 1H ), 6.49 (t, $J$ = 2.0 Hz, 1H), 2.46 (s, 3H); $^{13}$C NMR (125 MHz, $CDCl_3$) $\delta$ 167.3, 141.5, 139.7, 135.5, 134.6, 131.9, 130.5, 129.5, 129.4, 128.9, 128.4, 124.9, 124.7, 123.1, 122.7, 107.6, 20.3; HRMS (ESI-TOF) m/z: $[M + H]^+$ Calcd for $C_{17}H_{14}BrN_3O$ 356.0393; Found 356.0393.

***N*-(2-(1*H*-pyrazol-1-yl)phenyl)-5-chloro-2-methoxybenzamide (3ai)**. This compound was purified by column chromatography (ethyl acetate/petroleum ether = 1:10) to afford a white solid in 86 % yield (70 mg, 0.22 mmol); $^1$H NMR (500 MHz, $CDCl_3$) $\delta$ 10.77 (s, 1H), 8.60 (d, $J$ = 8.5 Hz, 1H), 8.17-8.13 (m, 1H), 7.82-7.79 (m, 1H), 7.75-7.71 (m, 1H), 7.43 (d, $J$ = 8.0 Hz, 1H), 7.39-7.37 (m, 1H), 7.30-7.25 (m, 1H), 7.19 (d, $J$ = 8.0 Hz, 1H), 6.87 (d, $J$ = 9.0 Hz, 1H), 6.50 (t, $J$ = 2.0 Hz, 1H), 3.85 (s, 3H); $^{13}$C NMR (125 MHz, $CDCl_3$) $\delta$ 162.8, 156.2, 141.3, 133.2, 133.1, 132.4, 131.1, 130.9, 129.1, 126.9, 125.2, 124.7, 124.2, 123.8, 113.1, 107.4, 56.4; HRMS (ESI-TOF) m/z: $[M + H]^+$ Calcd for $C_{17}H_{14}ClN_3O_2$ 328.0847; Found 328.0847.

***N*-(2-(1*H*-pyrazol-1-yl)phenyl)-3-bromobenzamide (3aj).** This compound was purified by column chromatography (ethyl acetate/petroleum ether = 1:10) to afford a white solid in 85 % yield (73 mg, 0.21 mmol); $^1$H NMR (500 MHz, $CDCl_3$) $\delta$ 11.48 (s, 1H), 8.68 (d, $J$ = 8.0 Hz, 1H), 8.10 (s, 1H), 7.89-7.86 (m, 3H), 7.66 (d, $J$ = 7.5 Hz, 1H), 7.43-7.34 (m, 3H), 7.21 (t, $J$ = 7.5 Hz, 1H), 6.54 (t, $J$ = 2.0 Hz, 1H); $^{13}$C NMR (125 MHz, $CDCl_3$) $\delta$ 164.1, 141.5, 137.2, 135.1, 131.7, 131.0, 130.6, 129.3, 128.4, 126.1, 124.6, 123.3, 123.2, 122.2, 107.8; HRMS (ESI-TOF) m/z: $[M + H]^+$ Calcd for $C_{16}H_{12}BrN_3O$ 342.0237; Found 342.0233.

***N*-(2-(1*H*-pyrazol-1-yl)phenyl)-4-fluorobenzamide (3ak).** This compound was purified by column chromatography (ethyl acetate/petroleum ether = 1:10) to afford a white solid in 89 % yield (62 mg, 0.22 mmol); $^1$H NMR (500 MHz, $CDCl_3$) $\delta$ 11.33 (s, 1H), 8.68 (d, $J$ = 8.0 Hz, 1H), 7.97-7.94 (m, 2H), 7.88-7.85 (m, 2H), 7.42-7.37 (m, 2H), 7.20 (t, $J$ =7.5 Hz, 1H), 7.15 (t, $J$ =8.5 Hz, 1H), 6.53 (t, $J$ =2.0 Hz, 1H); $^{13}$C NMR (125 MHz, $CDCl_3$) $\delta$ 165.3 (d, $J_{C\text{-}F}$ = 250.8 Hz ), 164.6, 141.4, 132.0, 131.4 (d, $J_{C\text{-}F}$ = 2.7 Hz ), 130.6, 130.0 (d, $J_{C\text{-}F}$ = 9.0 Hz ), 129.4, 128.4, 124.5, 123.2, 122.4, 116.1 (d, $J_{C\text{-}F}$ = 21.7 Hz ), 107.7; HRMS (ESI-TOF) m/z: $[M + H]^+$ Calcd for $C_{16}H_{12}FN_3O$ 282.1037; Found 282.1034.

***N*-(2-(1*H*-pyrazol-1-yl)phenyl)-4-chlorobenzamide (3al).** This compound was purified by column chromatography (ethyl acetate/petroleum ether = 1:10) to afford a white solid in 82 % yield (61 mg, 0.21 mmol); $^1$H NMR (500 MHz, $CDCl_3$) $\delta$ 11.35 (s, 1H), 8.65 (d, $J$ = 9.0 Hz, 1H), 7.89-7.86 (m, 4H), 7.46 (d, $J$ = 8.5 Hz, 1H), 7.43-7.37 (m, 2H), 7.21 (t, $J$ = 7.5 Hz, 1H), 6.53 (t, $J$

= 2.0 Hz, 1H); $^{13}C$ NMR (125 MHz, $CDCl_3$) $\delta$ 164.6, 141.6, 138.5, 133.6, 131.9, 130.7, 129.4, 129.3, 129.1, 128.5, 124.6, 123.3, 122.4, 107.7; HRMS (ESI-TOF) m/z: $[M + H]^+$ Calcd for $C_{16}H_{12}ClN_3O$ 298.0742; Found 298.0743.

***N*-(2-(1*H*-pyrazol-1-yl)phenyl)-4-bromobenzamide (3am).** This compound was purified by column chromatography (ethyl acetate/petroleum ether = 1:10) to afford a white solid in 91 % yield (78 mg, 0.23 mmol); $^1H$ NMR (500 MHz, $CDCl_3$) $\delta$ 11.40 (s, 1H), 8.68 (d, $J$ = 8.0 Hz, 1H), 7.87-7.86 (m, 2H), 7.81 (d, $J$ = 9.0 Hz, 2H), 7.62 (d, $J$ = 8.5 Hz, 2H), 7.42-7.37 (m, 2H), 7.22-7.19 (m, 2H), 6.53 (d, $J$ = 2.0 Hz, 1H); $^{13}C$ NMR (125 MHz, $CDCl_3$) $\delta$ 164.7, 141.5, 134.1, 132.3, 131.8, 130.6, 129.3, 129.2, 128.4, 126.9, 124.6, 107.7; HRMS (ESI-TOF) m/z: $[M + H]^+$ Calcd for $C_{16}H_{12}BrN_3O$ 342.0237; Found 342.0234.

***N*-(2-(1*H*-pyrazol-1-yl)phenyl)-4-(trifluoromethyl)benzamide (3an).** This compound was purified by column chromatography (ethyl acetate/petroleum ether = 1:10) to afford a white solid in 79 % yield (65 mg, 0.20 mmol); $^1H$ NMR (500 MHz, $CDCl_3$) $\delta$ 11.56 (s, 1H), 8.70 (d, $J$ = 9.0 Hz, 1H), 8.06 (d, $J$ = 8.5 Hz, 2H), 7.89-7.87 (m, 2H), 7.76 (d, $J$ = 8.0 Hz, 2H), 7.44-7.39 (m, 2H), 7.23 (t, $J$ = 7.0 Hz, 1H), 6.54 (t, $J$ = 2.0 Hz, 1H); $^{13}C$ NMR (125 MHz, $CDCl_3$) $\delta$ 164.3, 141.5, 138.5, 133.8 (q, $J_{C\text{-}F}$ = 32.5 Hz), 131.7, 130.6, 129.4, 128.4, 128.1, 127.3, 126.1 (q, $J_{C\text{-}F}$ = 3.7 Hz), 124.8, 124.1 (q, $J_{C\text{-}F}$ = 270.8 Hz),123.2, 122.3, 107.8; HRMS (ESI-TOF) m/z: $[M + H]^+$ Calcd for $C_{17}H_{12}F_3N_3O$ 332.1005; Found 332.1001.

***N*-(2-(1*H*-pyrazol-1-yl)phenyl)-2-naphthamide (3ao).** This compound was purified by column chromatography (ethyl acetate/petroleum ether = 1:10) to afford a white solid in 81 % yield (67 mg, 0.20 mmol); $^1H$ NMR (500 MHz, $CDCl_3$) $\delta$ 11.48 (s, 1H), 8.76 (d, $J$ = 8.5 Hz, 1H), 8.46 (s, 1H), 8.01-7.88 (m, 6H), 7.60-7.54 (m, 2H), 7.46-7.39 (m, 2H), 7.24-7.20 (m, 1H); 6.54 (t, $J$ = 2.0 Hz, 1H); $^{13}C$ NMR (125 MHz, $CDCl_3$) $\delta$ 165.7, 141.5, 135.3, 133.1, 132.5, 132.3, 130.7, 129.5, 129.5, 128.9, 128.5, 128.5, 128.2, 128.1, 127.1, 124.4, 124.1, 123.3, 122.5, 107.7; HRMS (ESI-TOF) m/z: $[M + H]^+$ Calcd for $C_{20}H_{15}N_3O$ 314.1288; Found 314.1284.

***N*-(2-(1*H*-pyrazol-1-yl)phenyl)-2-phenylacetamide (3ap).** This compound was purified by column chromatography (ethyl acetate/petroleum ether = 1:10) to afford a white solid in 88 % yield (61 mg, 0.22 mmol); $^1H$ NMR (500 MHz, $CDCl_3$) $\delta$ 10.03 (s, 1H), 8.49 (d, $J$ = 8.0 Hz, 1H), 7.67-7.66 (m, 1H), 7.42-7.40 (m, 1H), 7.36-7.31 (m, 4H), 7.26-7.23 (m, 3H), 7.12 (t, $J$ = 8.0 Hz, 1H), 6.36 (t, $J$ = 2.0 Hz, 1H), 3.70 (s, 2H); $^{13}C$ NMR (125 MHz, $CDCl_3$) $\delta$ 170.1, 141.3, 134.5, 132.0, 130.1, 129.4, 129.3, 128.3, 127.7, 124.4, 123.2, 122.8, 107.1, 45.8; HRMS (ESI-TOF) m/z:

[M + H]$^+$ Calcd for $C_{17}H_{15}N_3O$ 278.1288; Found 278.1289.

**(*E*)-*N*-(2-(1*H*-pyrazol-1-yl)phenyl)-3-(p-tolyl)acrylamide (3at).** This compound was purified by column chromatography (ethyl acetate/petroleum ether = 1:10) to afford a white solid in 77 % yield (58 mg, 0.19 mmol); $^1$H NMR (500 MHz, $CDCl_3$) $\delta$ 10.56 (s, 1H), 8.65 (d, $J$ = 8.5 Hz, 1H), 7.87-7.83 (m, 2H), 7.67 (d, $J$ = 9.5 Hz, 1H), 7.44 (d, $J$ = 7.5 Hz, 2H), 7.38 (t, $J$ = 7.5 Hz, 1H), 7.33 (d, $J$ = 8.0 Hz, 1H), 7.20-7.15 (m, 3H); 6.52 (t, $J$ = 2.0 Hz, 1H), 6.45 (m, 1H); $^{13}$C NMR (125 MHz, $CDCl_3$) $\delta$ 164.6, 142.2, 141.5, 140.6, 132.3, 132.3, 130.6, 129.9, 129.2, 128.4, 128.3, 124.2, 123.3, 122.7, 121.1, 107.6, 21.8; HRMS (ESI-TOF) m/z: [M + H]$^+$ Calcd for $C_{19}H_{17}N_3O$ 304.1444; Found 304.1441.

***N*-(2-(1*H*-pyrazol-1-yl)phenyl)thiophene-2-carboxamide (3au).** This compound was purified by column chromatography (ethyl acetate/petroleum ether = 1:10) to afford a white solid in 71 % yield (50 mg, 0.18 mmol); $^1$H NMR (500 MHz, $CDCl_3$) $\delta$ 11.33 (s, 1H), 8.64 (d, $J$ = 9.0 Hz, 1H), 7.89-7.86 (m, 2H), 7.64-7.63 (m, 1H), 7.54-7.52 (m, 1H), 7.41-7.36 (m, 2H), 7.20-7.17 (m, 1H), 7.12 (t, $J$ = 9.0 Hz, 1H), 6.53 (t, $J$ = 2.0 Hz, 1H); $^{13}$C NMR (125 MHz, $CDCl_3$) $\delta$ 160.3, 141.4, 140.6, 131.9, 131.2, 130.6, 129.1, 128.7, 128.4, 128.1, 124.3, 123.1, 122.3, 107.7; HRMS (ESI-TOF) m/z: [M + H]$^+$ Calcd for $C_{14}H_{11}N_3OS$ 270.0696; Found 270.0693.

***N*-(2-(1*H*-pyrazol-1-yl)phenyl)furan-3-carboxamide (3av).** This compound was purified by column chromatography (ethyl acetate/petroleum ether = 1:10) to afford a white solid in 70 % yield (44 mg, 0.17 mmol); $^1$H NMR (500 MHz, $CDCl_3$) $\delta$ 11.03 (s, 1H), 8.64 (d, $J$ = 8.5 Hz, 1H), 8.03 (s, 1H), 7.87-7.86 (m, 2H), 7.46 (t, d, $J$ = 2.0 Hz, 1H), 7.40-7.35 (m, 2H), 7.18 (t, d, $J$ = 8.5 Hz, 1H), 6.76 (s, 1H), 6.53 (t, d, $J$ = 2.0 Hz, 1H); $^{13}$C NMR (125 MHz, $CDCl_3$) $\delta$ 160.9, 145.7, 144.2, 141.3, 131.9, 130.6, 129.0, 128.4, 124.3, 124.0, 123.0, 122.4, 108.7, 107.7; HRMS (ESI-TOF) m/z: [M + H]$^+$ Calcd for $C_{14}H_{11}N_3O_2$ 254.0926; Found 254.0924.

***N*-(2-(1*H*-pyrazol-1-yl)phenyl)cyclohexanecarboxamide (3aw).** This compound was purified by column chromatography (ethyl acetate/petroleum ether = 1:10) to afford a white solid in 86 % yield (58 mg, 0.21 mmol); $^1$H NMR (500 MHz, $CDCl_3$) $\delta$ 10.19 (s, 1H), 8.49 (d, $J$ = 8.5 Hz, 1H), 7.81-7.78 (m, 2H), 7.34 (t, $J$ = 8.5 Hz, 1H), 7.29-7.25 (m, 1H), 7.13 (t, $J$ = 7.0 Hz, 1H), 6.50 (t, $J$ = 2.0 Hz, 1H); 2.24-2.18 (m, 2H), 1.93-1.91 (m, 2H), 1.81-1.77 (m, 2H), 1.69-1.66 (m, 1H), 1.49-1.41 (m, 2H), 1.34-1.18 (m, 3H); $^{13}$C NMR (125 MHz, $CDCl_3$) $\delta$ 175.0, 141.4, 132.3, 130.6, 129.4, 128.4, 124.1, 123.4, 122.8, 107.4, 47.1, 29.8, 26.1, 26.0; HRMS (ESI-TOF) m/z: [M + H]$^+$ Calcd for $C_{16}H_{19}N_3O$ 270.1607; Found 270.1604.

***N*-(2-(pyridin-2-yl)phenyl)benzamide (9aa)**. This compound was purified by column chromatography (ethyl acetate/petroleum ether = 1:10) to afford a white solid in 84% yield (57 mg, 0.21 mmol); $^1$H NMR (500 MHz, CDCl$_3$) $\delta$ 13.11 (br, 1H), 8.72-8.67 (m, 2H), 8.06-8.02 (m, 2H), 7.90-7.87 (m, 1H), 7.82 (d, $J$ = 8.5 Hz, 1H), 7.73-7.71 (m, 1H), 7.55-7.47 (m, 4H), 7.32 (m, 1H), 7.22 (t, $J$ = 8.0 Hz, 1H); $^{13}$C NMR (125 MHz, CDCl$_3$) $\delta$ 165.9, 158.6, 147.5, 138.4, 138.4, 136.1, 131.9, 130.7, 129.1, 128.9, 127.7, 126.0, 124.0, 123.4, 122.5, 122.4; HRMS (ESI-TOF) m/z: [M + H]$^+$ Calcd for $C_{18}H_{14}N_2O$ 275.1179; Found 275.1176.

***N*-(2-(pyrimidin-2-yl)phenyl)benzamide (10aa).** This compound was purified by column chromatography (ethyl acetate/petroleum ether = 1:10) to afford a white solid in 89% yield (61 mg, 0.22 mmol); $^1$H NMR (500 MHz, CDCl$_3$) $\delta$ 13.53, 8.89-8.85 (m, 3s), 8.67 (dd, $J$ = 8.0, 1.5 Hz, 1H), 8.01 (d, $J$ = 8.5 Hz, 2H), 7.55 (t, $J$ = 7.0 Hz, 1H), 7.50 (d, $J$ = 9.0 Hz, 2H), 7.28-7.22 (m, 1H); $^{13}$C NMR (125 MHz, CDCl$_3$) $\delta$ 165.1, 164.8, 139.9, 137.8, 134.4, 132.4, 130.7, 128.9, 128.8, 123.4, 122.8, 121.0, 118.6. HRMS (ESI-TOF) m/z: [M + H]$^+$ Calcd for $C_{17}H_{13}ClN_3O$ 310.0742; Found 310.0740.

***N*-(1-(pyrimidin-2-yl)-1*H*-indol-2-yl)benzamide (11aa)**. This compound was purified by column chromatography (ethyl acetate/petroleum ether = 1:10) to afford a white solid in 81% yield (63 mg, 0.20 mmol); $^1$H NMR (500 MHz, CDCl$_3$) $\delta$ 13.06 (s, 1H), 8.82-8.80 (m, 2H), 8.74-8.71 (m, 1H), 8.02-8.00 (m, 2H), 7.59-7.51 (m, 4H), 7.47 (s, 1H), 7.25-7.24 (m, 1H), 7.18-7.16 (m, 1H); $^{13}$C NMR (125 MHz, CDCl$_3$) $\delta$ 164.1, 159.3, 158.0, 135.9, 135.1, 133.0, 132.2, 130.4, 129.2, 127.5, 123.6, 122.9, 120.3, 116.7, 116.4, 96.3; HRMS (ESI-TOF) m/z: [M + H]$^+$ Calcd for $C_{19}H_{14}N_4O$ 315.1240; Found 315.1239.

**4-bromo-*N*-(1-(pyrimidin-2-yl)-1*H*-inden-2-yl)benzamide (11am)**. This compound was purified by column chromatography (ethyl acetate/petroleum ether = 1:10) to afford a white solid in 95% yield (93 mg, 0.24 mmol); $^1$H NMR (500 MHz, CDCl$_3$) $\delta$ 13.08 (s, 1H), 8.76-8.72 (m, 2H), 8.71-8.68 (m, 1H), 7.84 (d, $J$ = 8.5 Hz, 2H), 7.65 (d, $J$ = 8.5 Hz, 2H), 7.55-7.53 (m, 1H), 7.41 (s, 1H), 7.24-7.21 (m, 2H), 7.14-7.12 (m, 1H); $^{13}$C NMR (125 MHz, CDCl$_3$) $\delta$ 163.1, 159.2, 158.0, 135.7, 133.9, 133.0, 132.4, 130.3, 129.0, 127.0, 123.7, 123.1, 120.4, 116.8, 116.5, 96.5; HRMS (ESI-TOF) m/z: [M + H]$^+$ Calcd for $C_{19}H_{13}BrN_4O$ 393.0346; Found 393.0344.

## 8. Copies of $^{1}H$ and $^{13}C$ NMR Spectra for all Compounds

## $^{1}H$ NMR and $^{13}C$ NMR Spectra of Compound 3aa

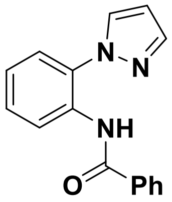


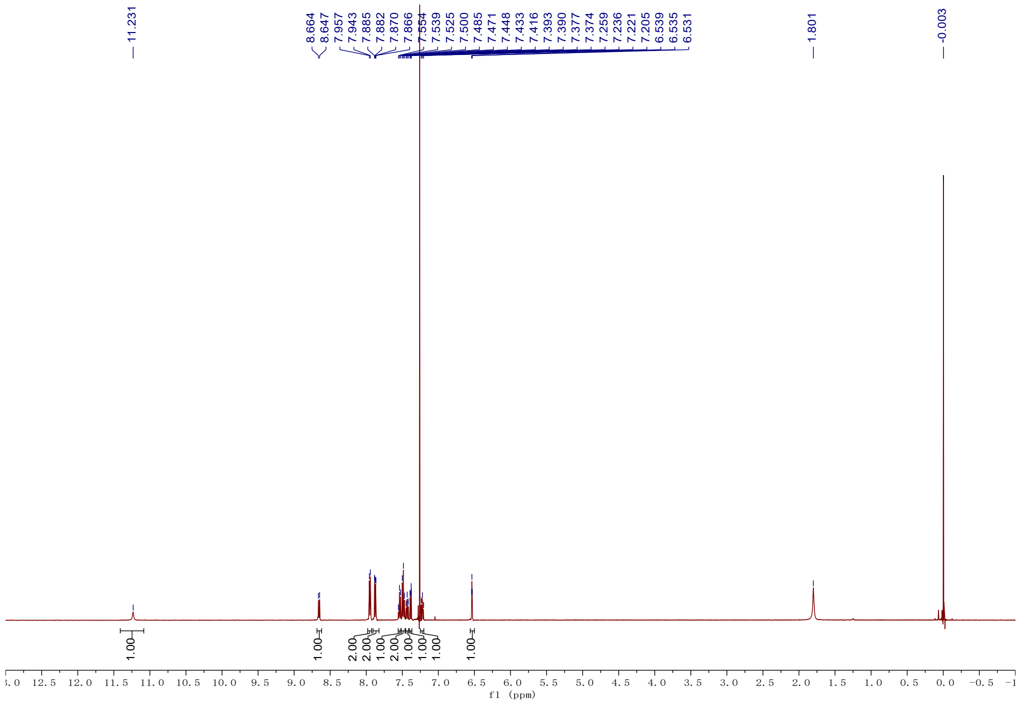


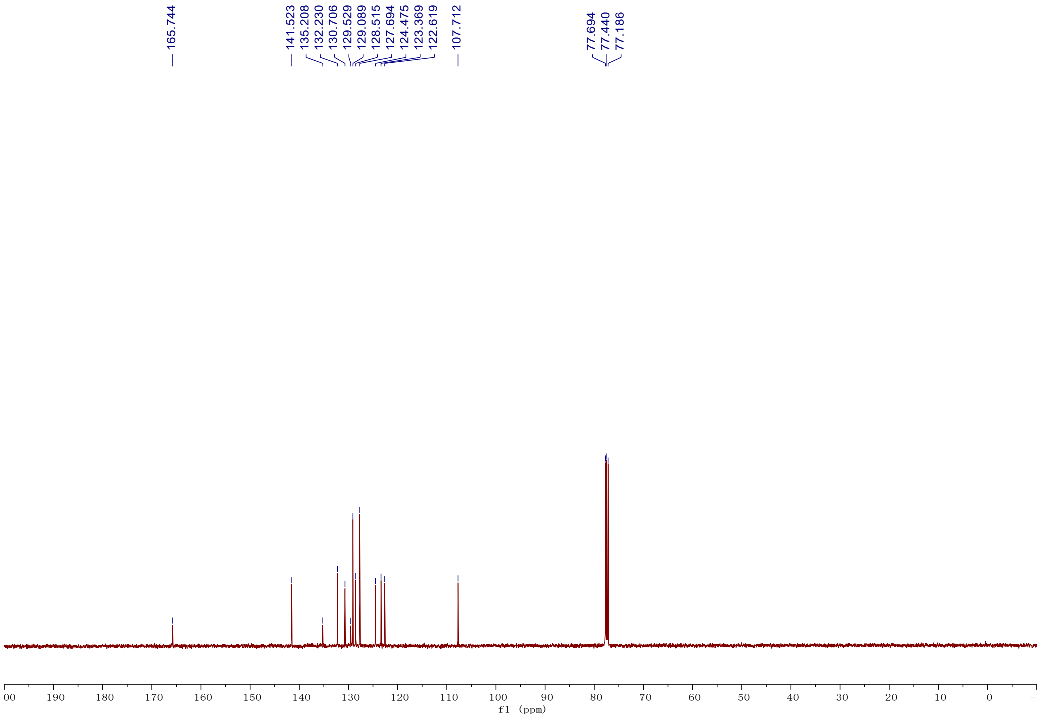

## $^{1}H$ NMR and $^{13}C$ NMR Spectra of Compound 3ba

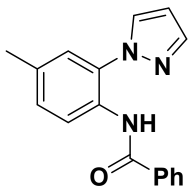


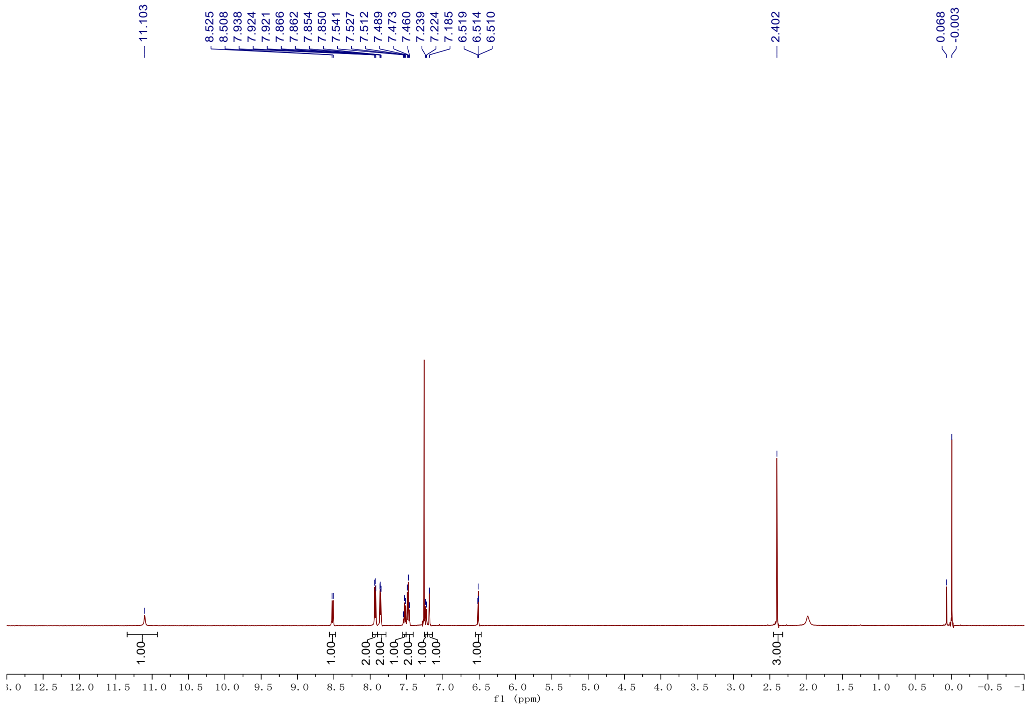


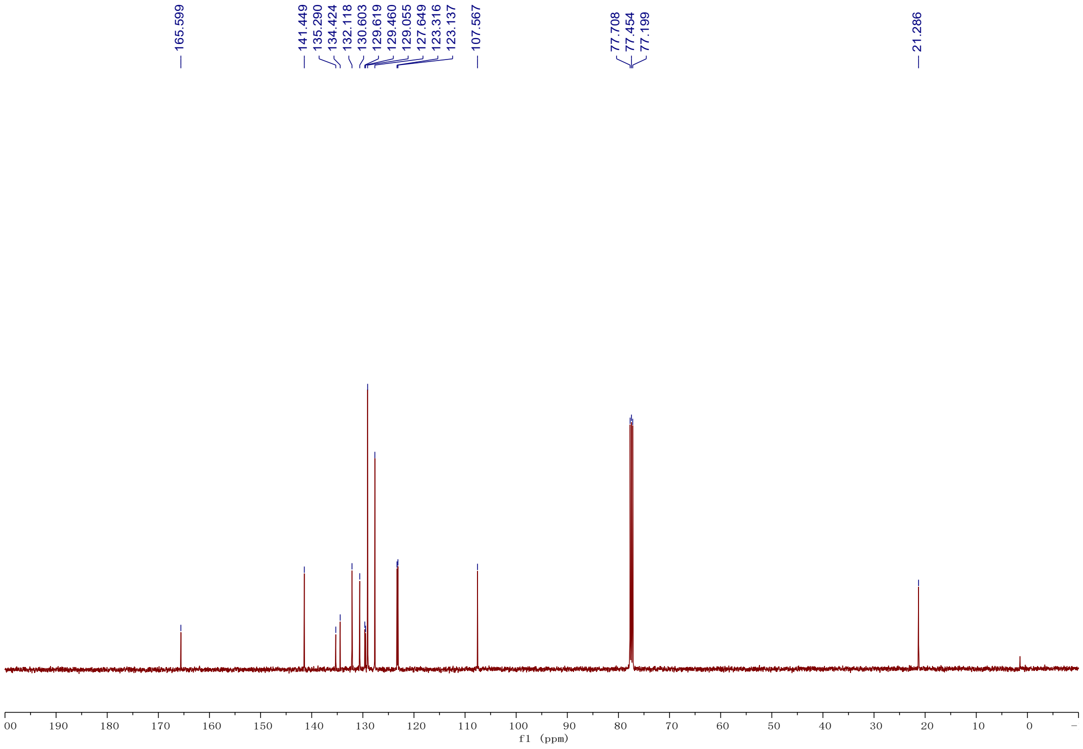

## $^{1}$H NMR and $^{13}$C NMR Spectra of Compound 3ca

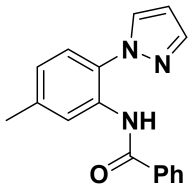


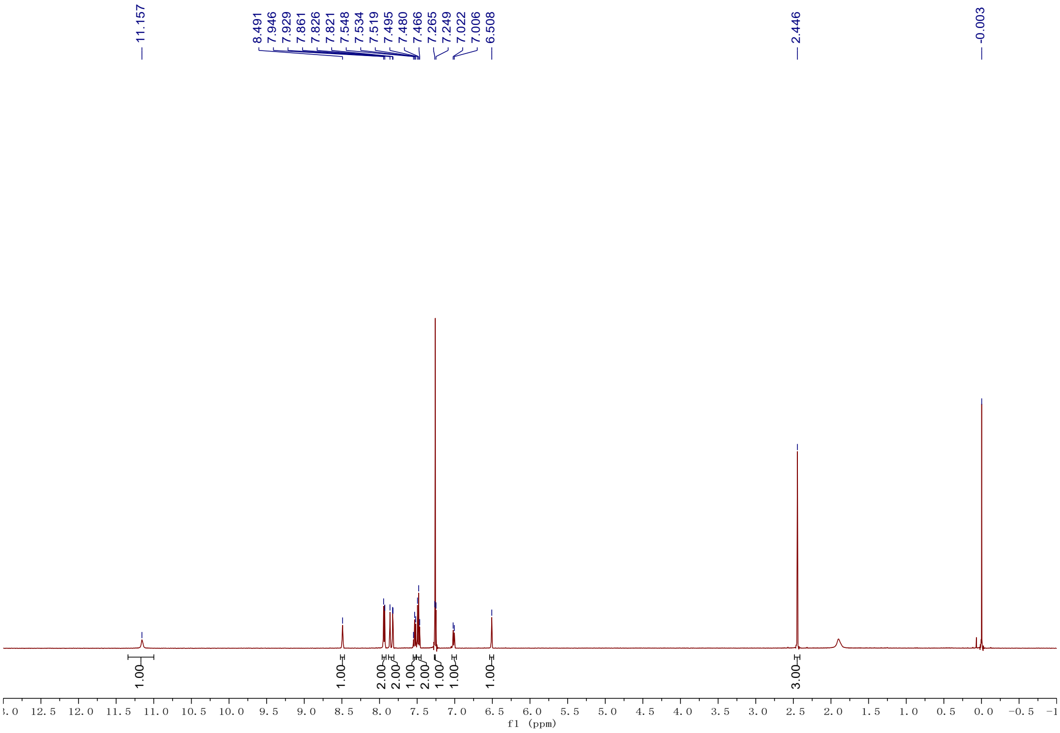


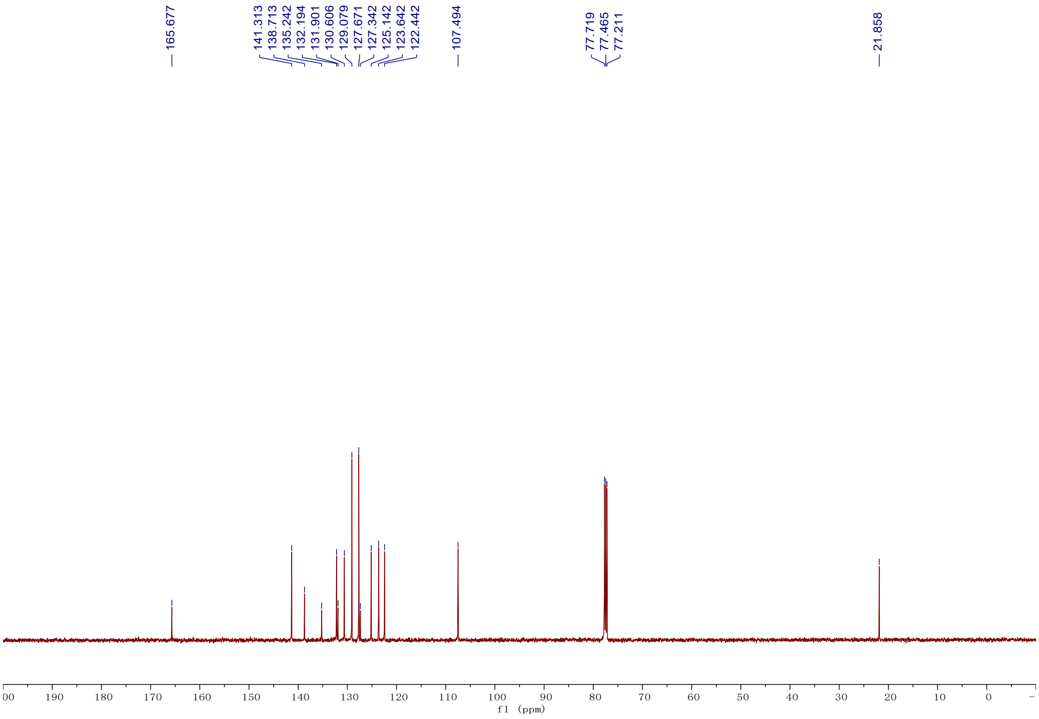

## $^{1}H$ NMR and $^{13}C$ NMR Spectra of Compound 3da

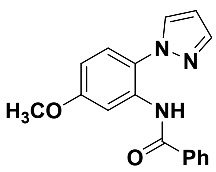


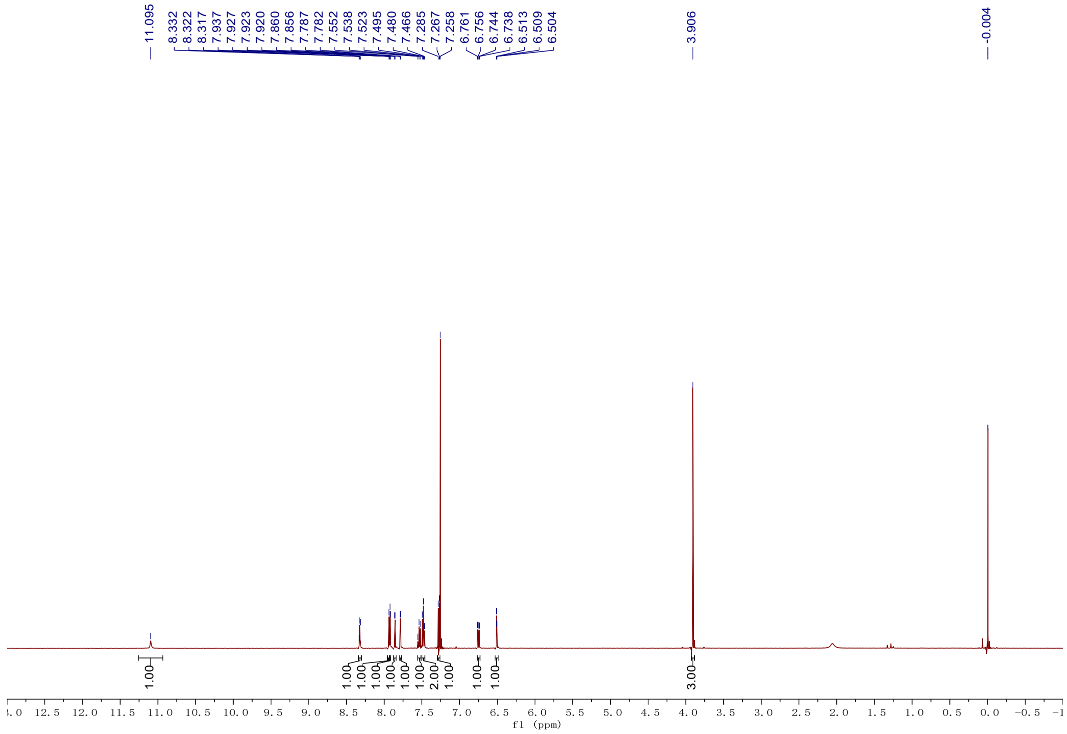


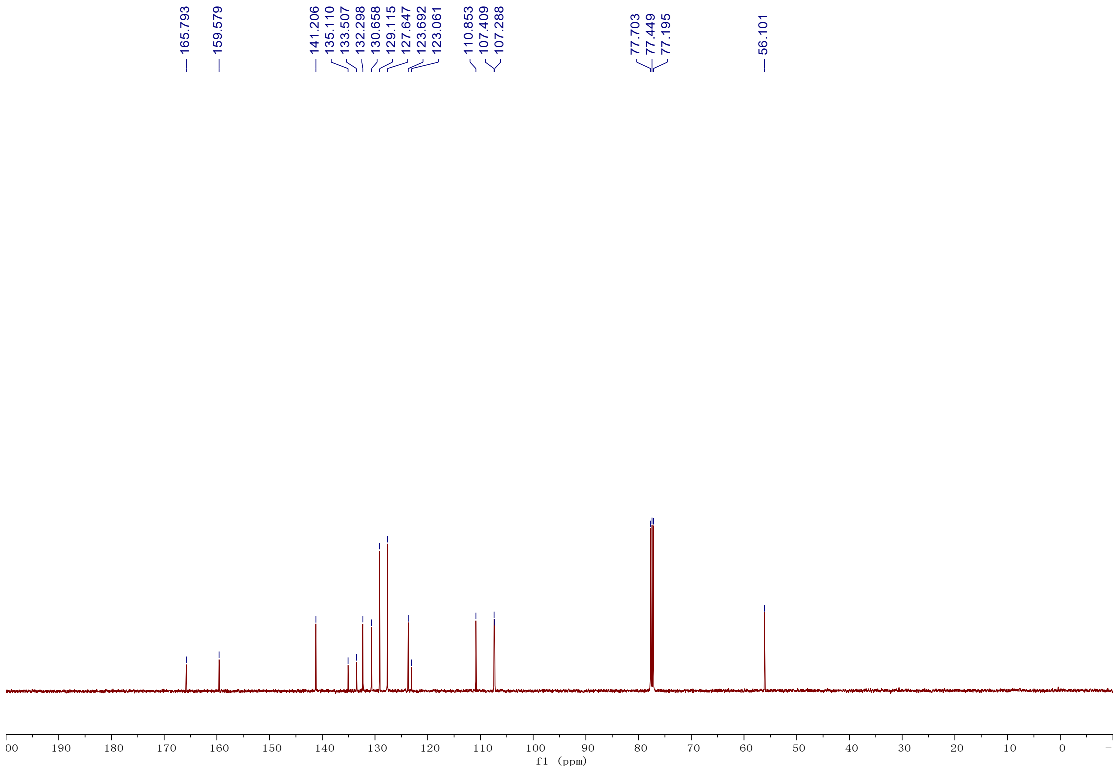

## $^1H$ NMR and $^{13}C$ NMR Spectra of Compound 3ea

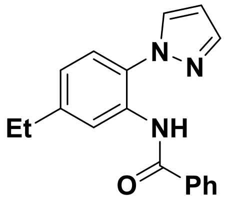


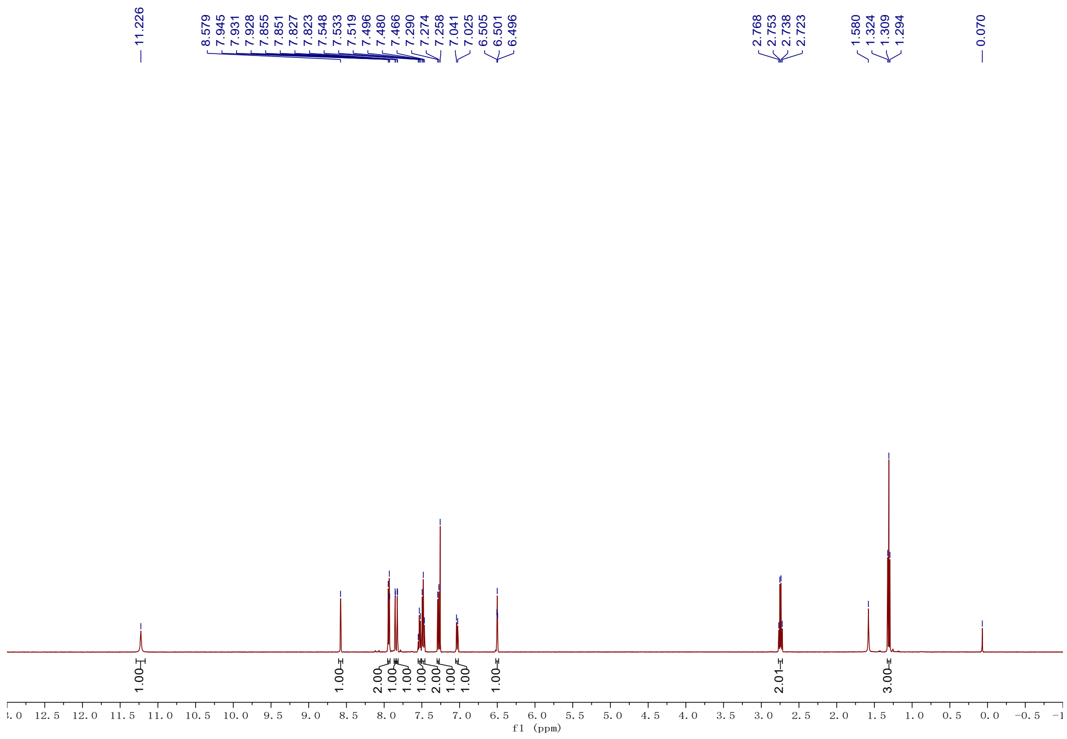


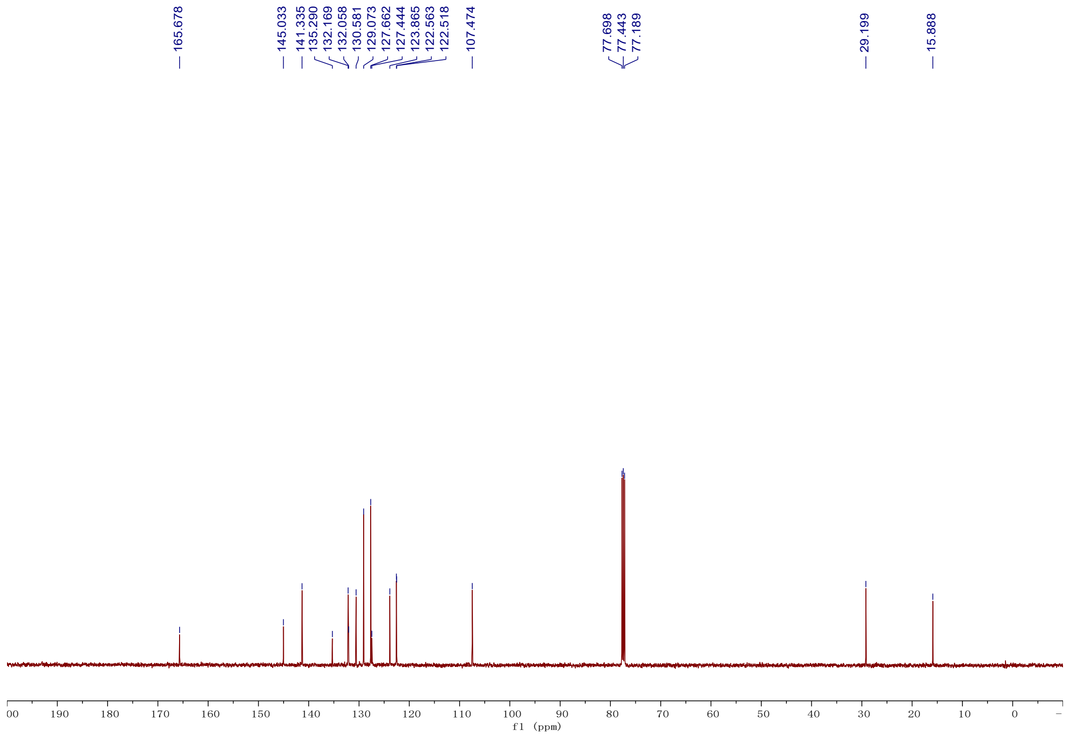

## $^{1}$H NMR and $^{13}$C NMR Spectra of Compound 3fa

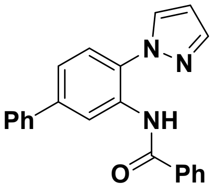


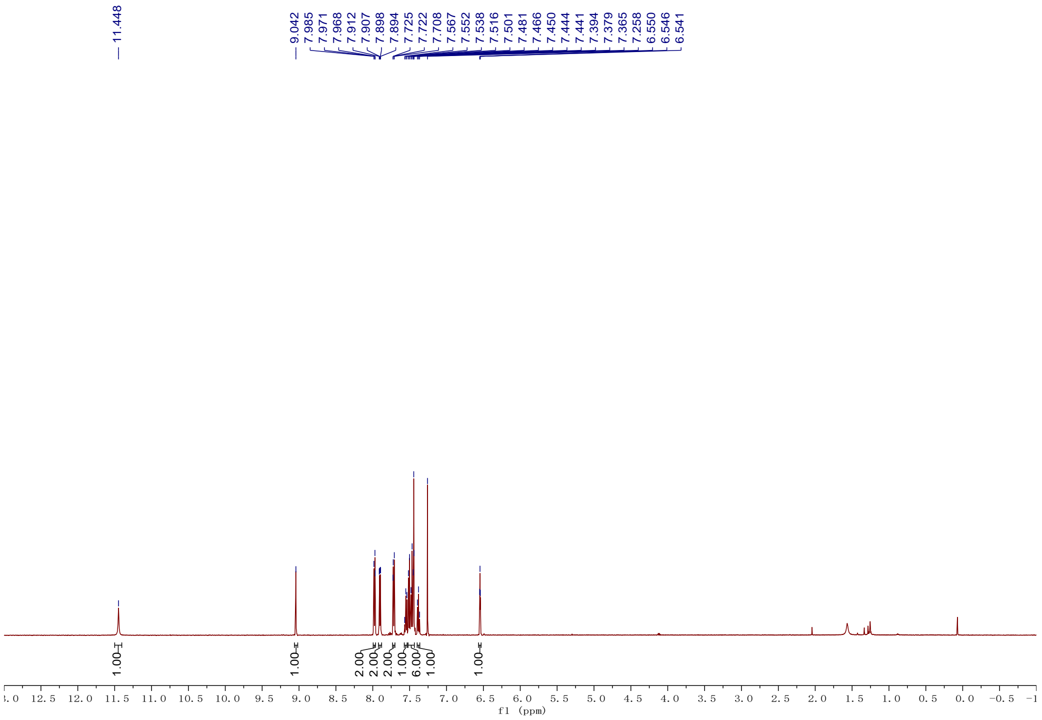


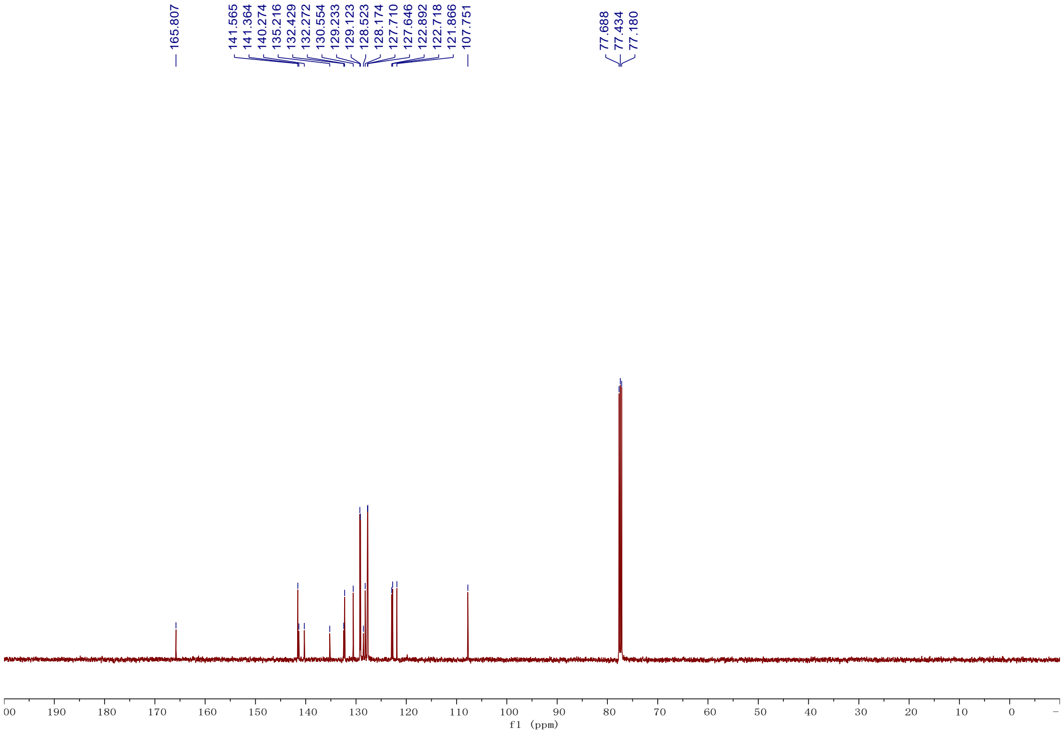

## $^{1}$H NMR and $^{13}$C NMR Spectra of Compound 3ga

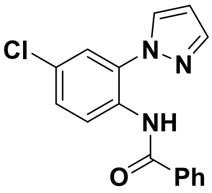

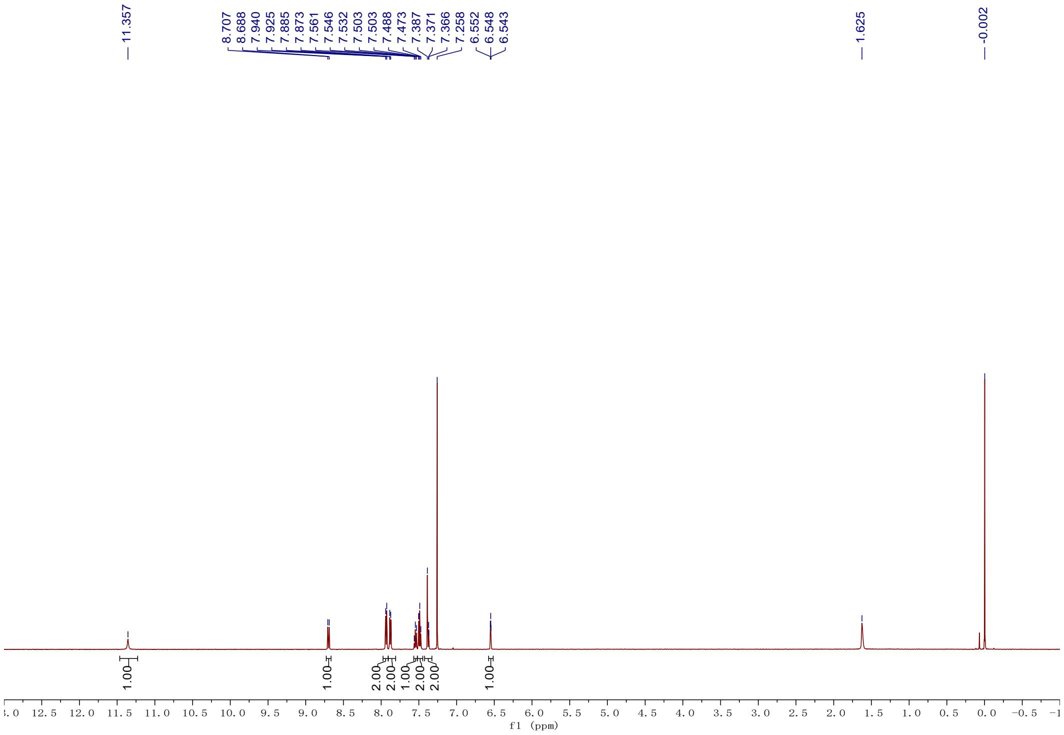


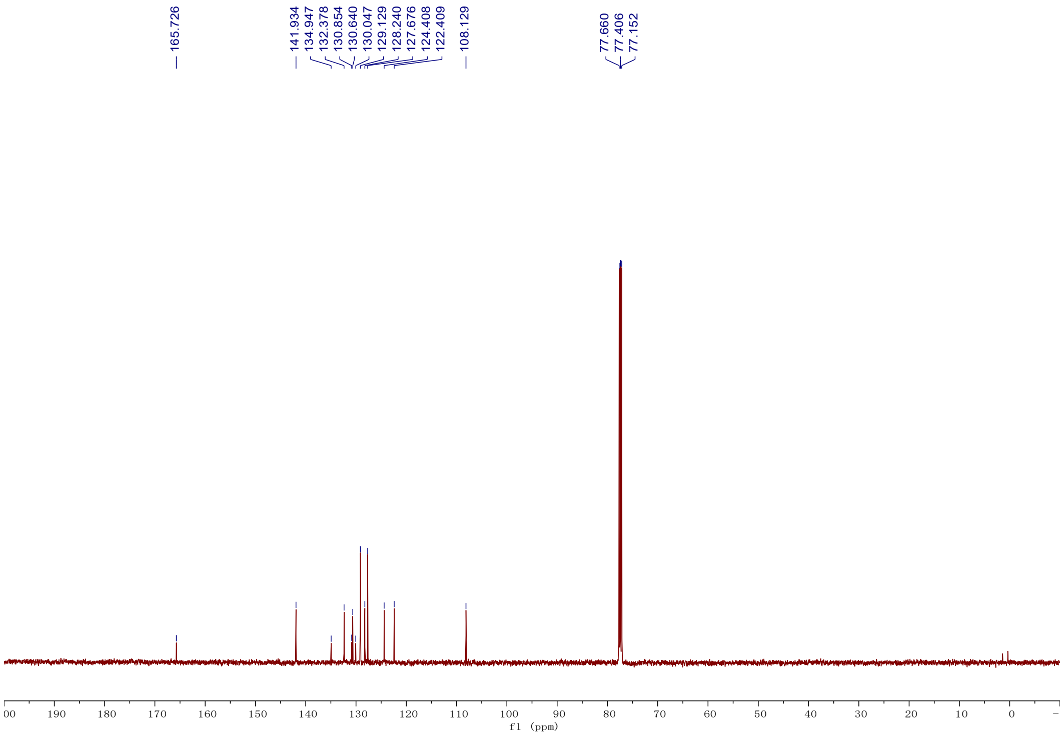

# $^{1}H$ NMR and $^{13}C$ NMR Spectra of Compound 3ha

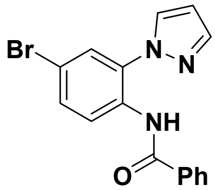


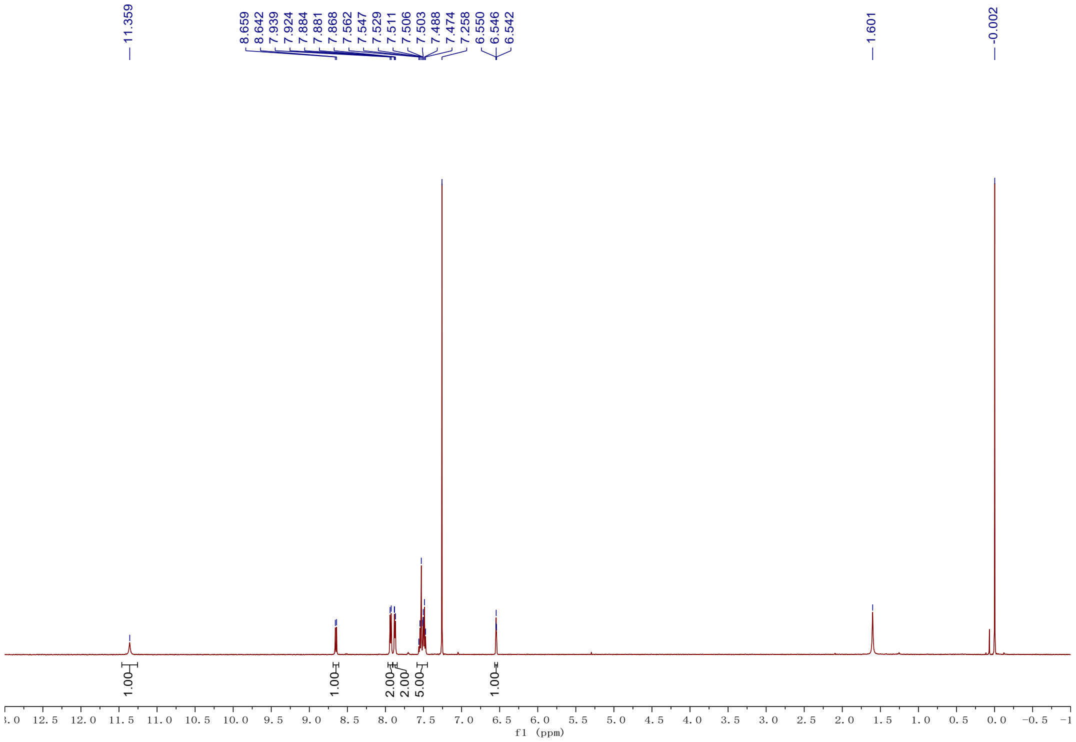


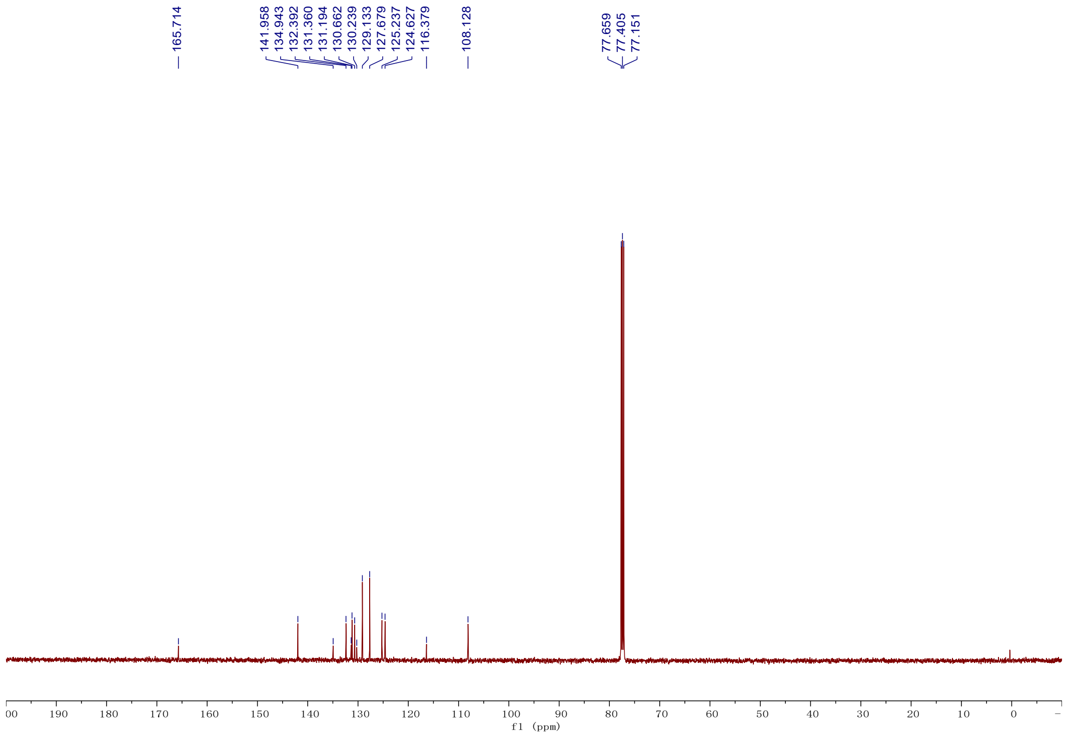

## $^{1}$H NMR and $^{13}$C NMR Spectra of Compound 3ia

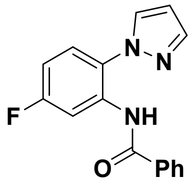


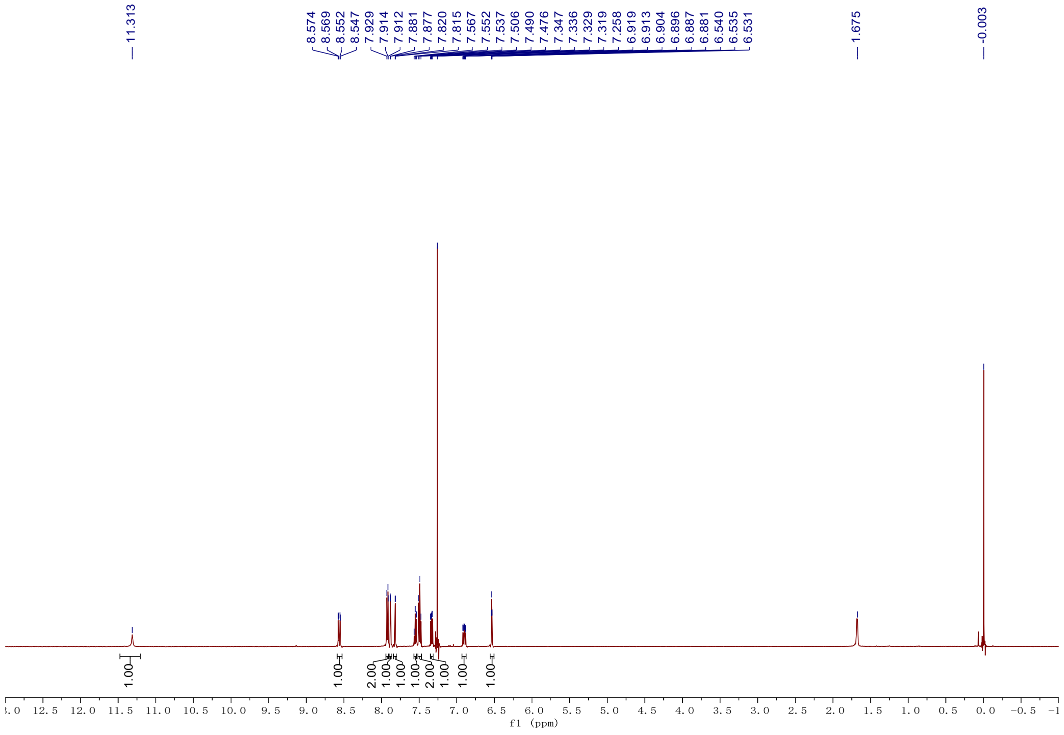


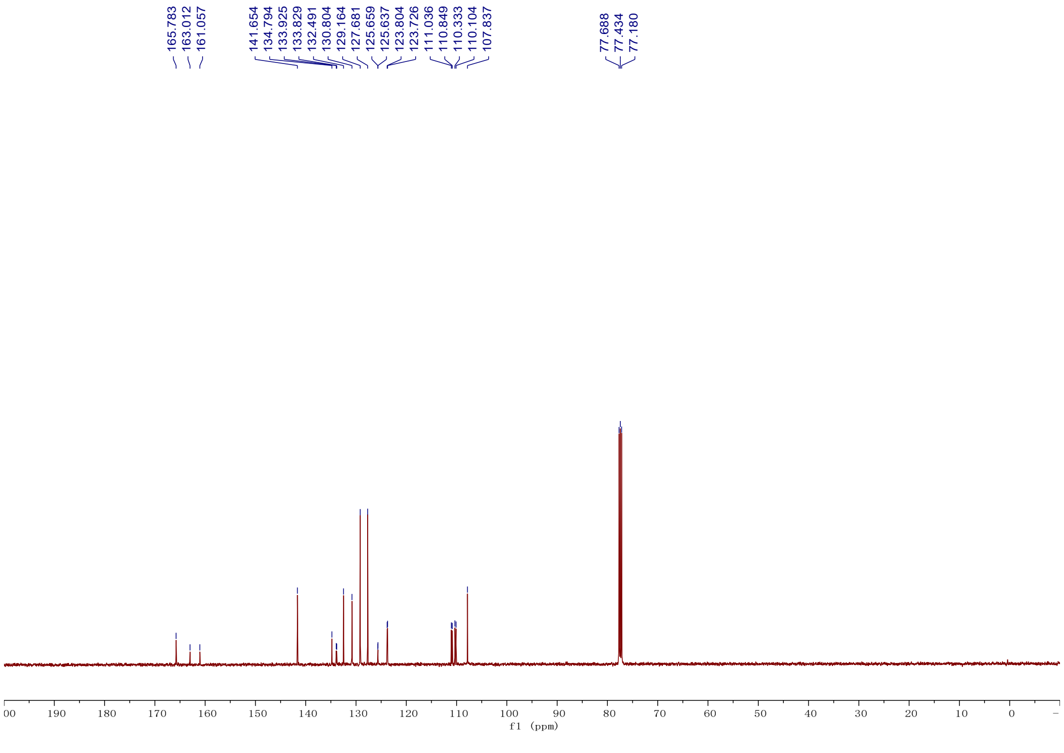

**$^1$H NMR and $^{13}$C NMR Spectra of Compound 3ja**

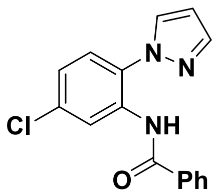

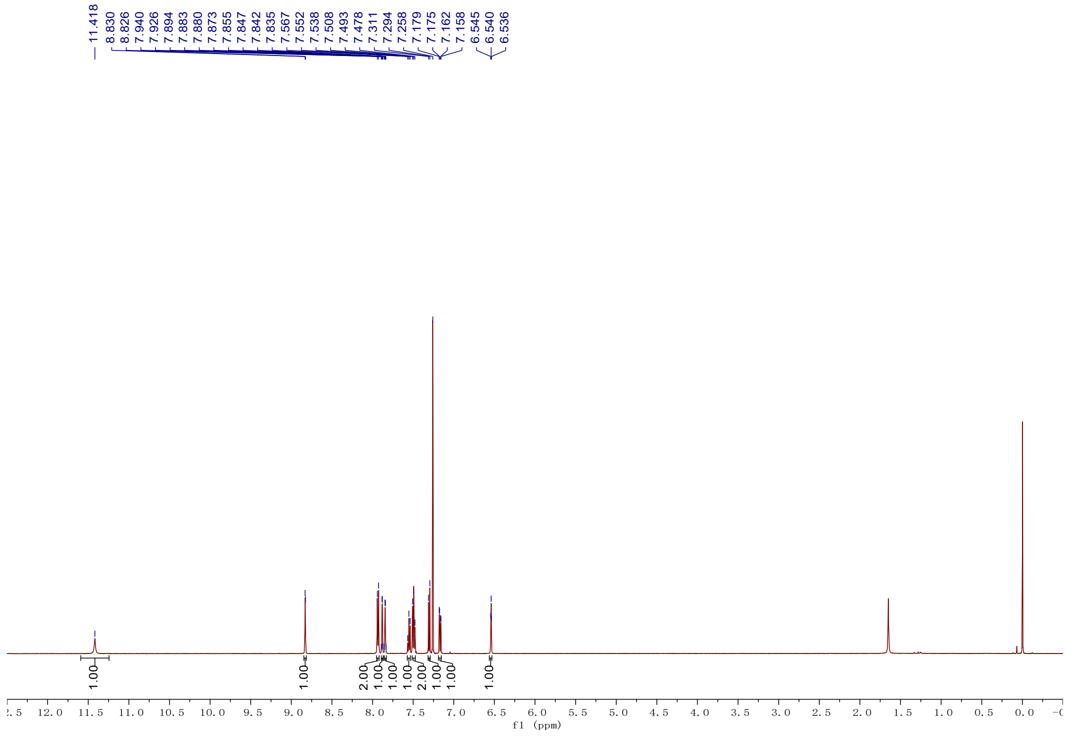

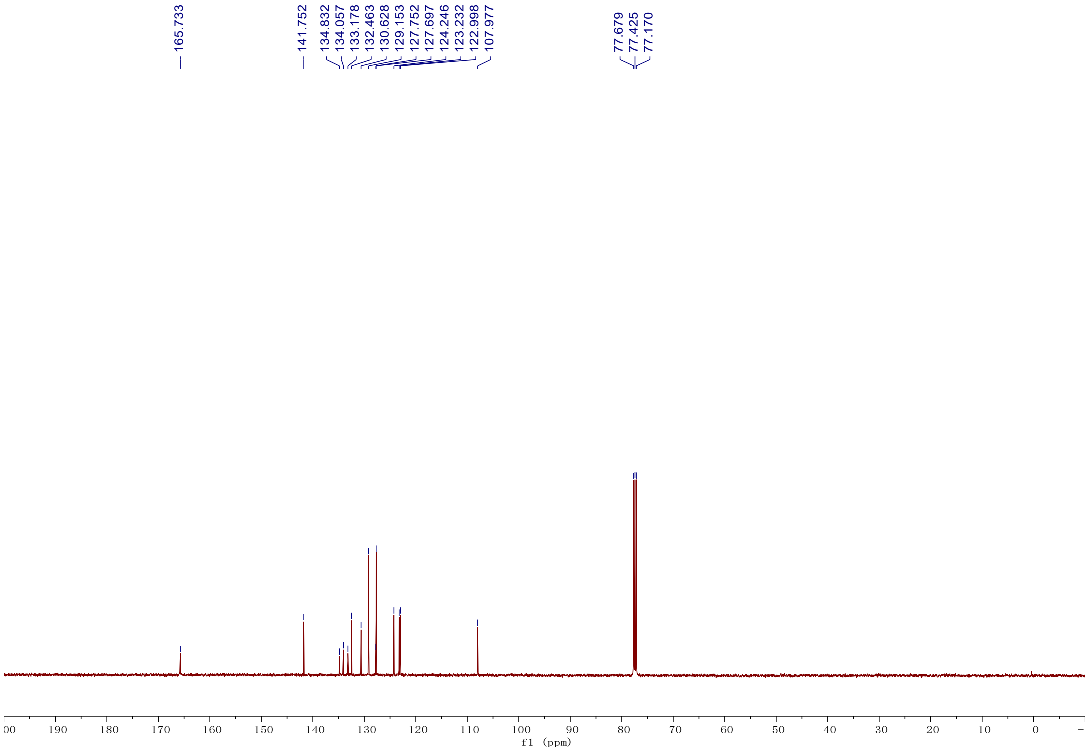

## $^{1}$H NMR and $^{13}$C NMR Spectra of Compound 3ka

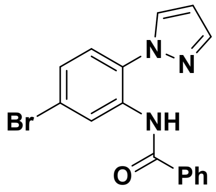


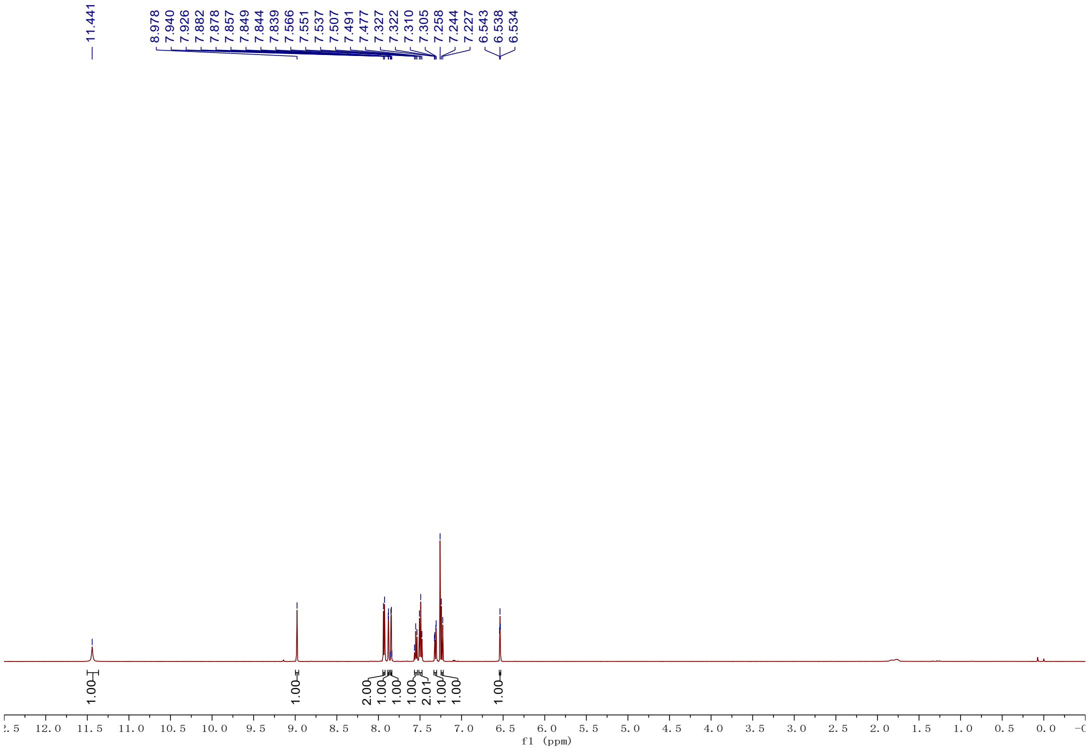


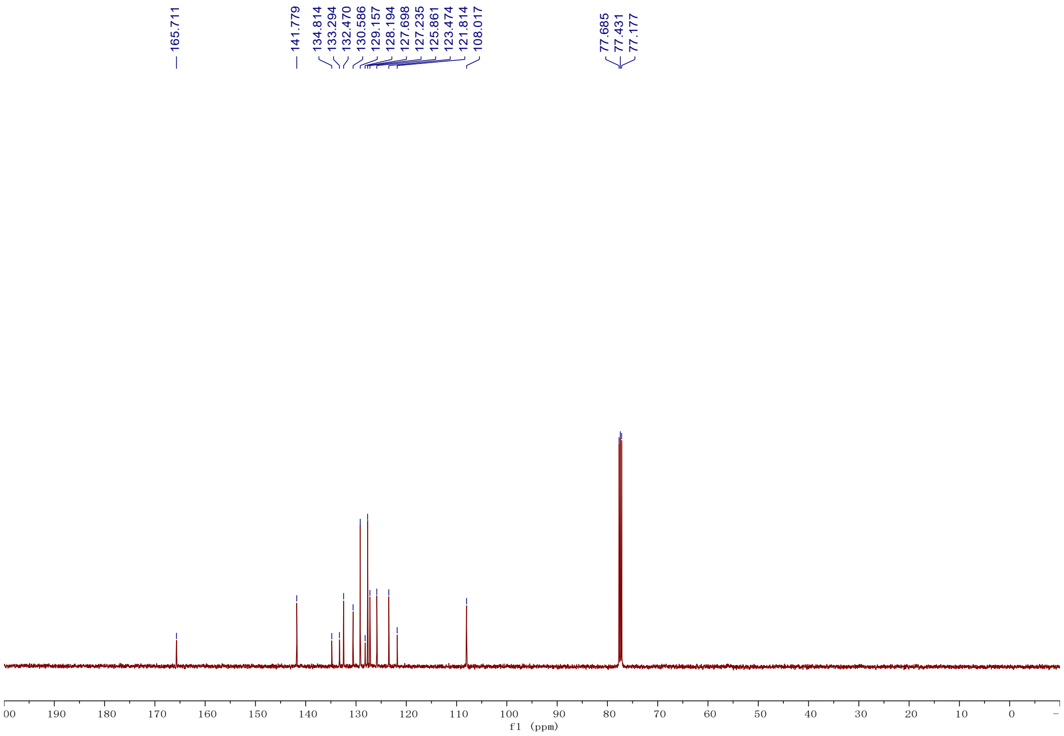

## $^1$H NMR and $^{13}$C NMR Spectra of Compound 3la

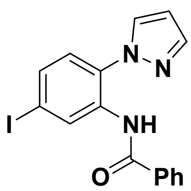


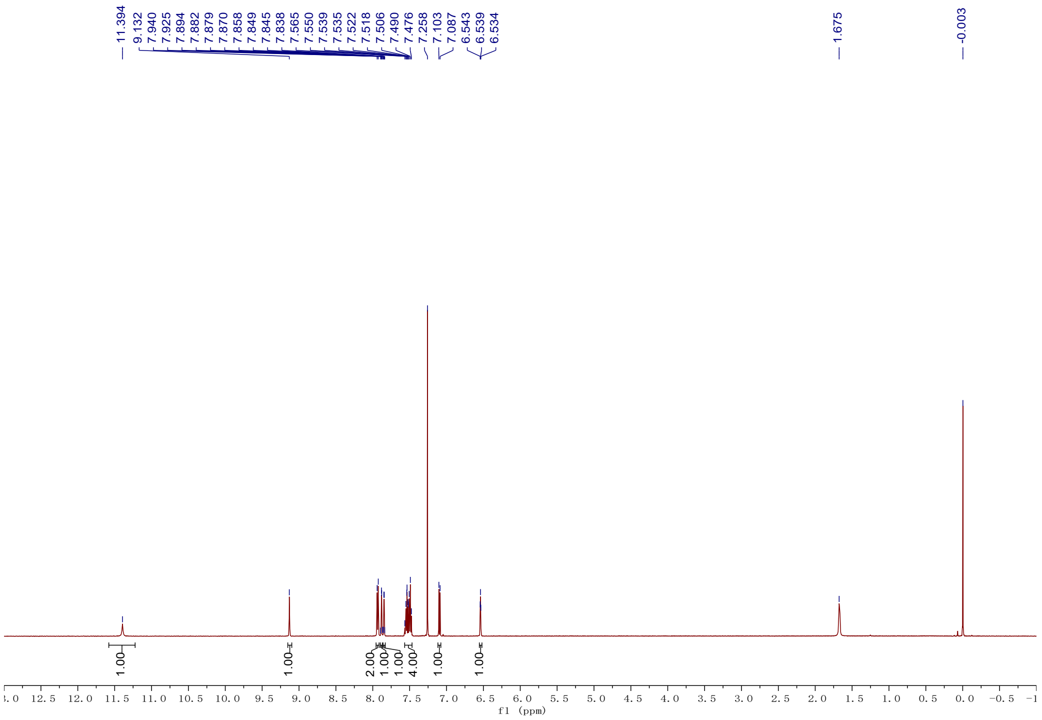


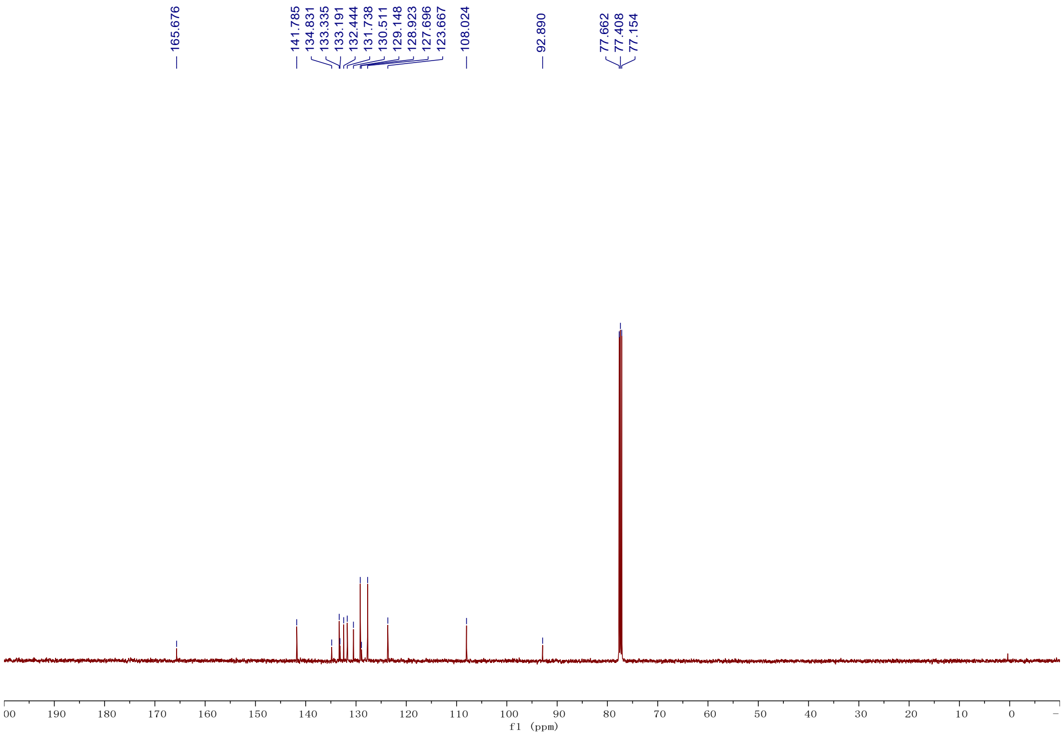

**$^{1}$H NMR and $^{13}$C NMR Spectra of Compound 3ma**

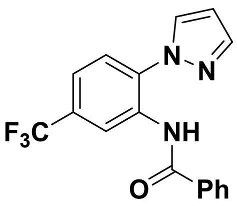


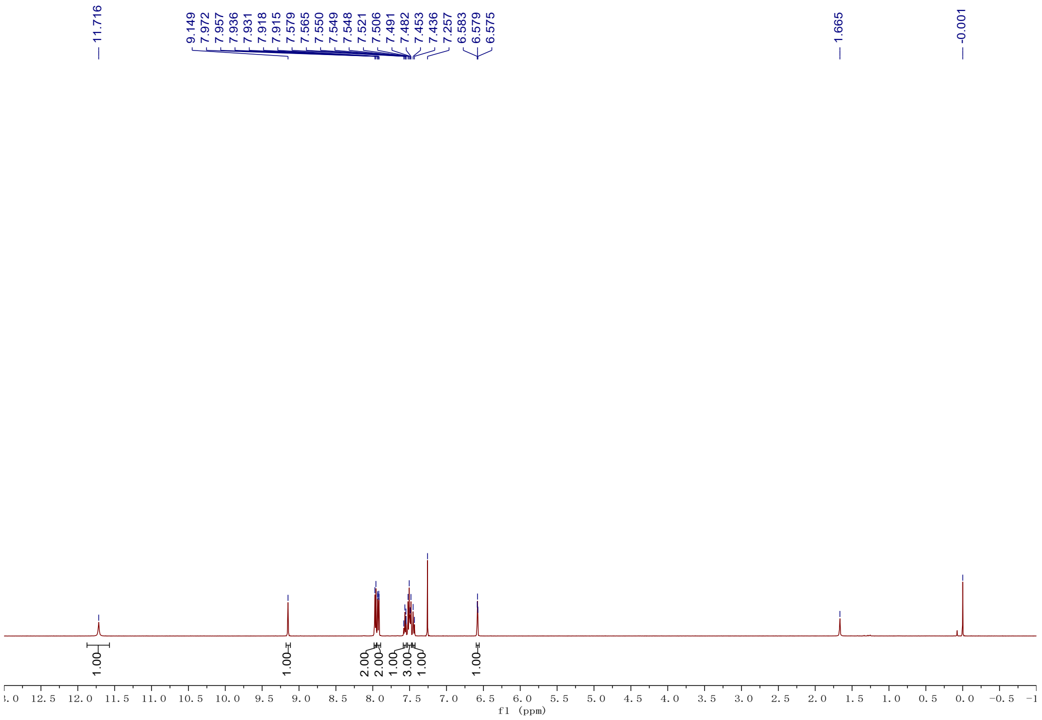

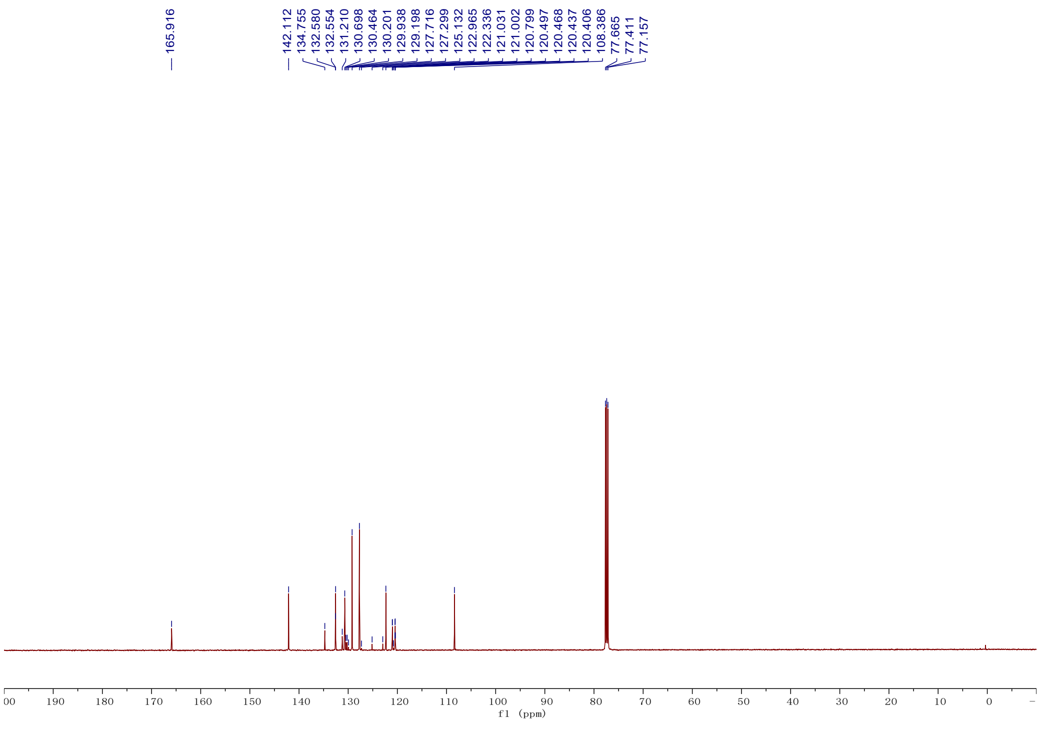

**$^{1}$H NMR and $^{13}$C NMR Spectra of Compound 3na**

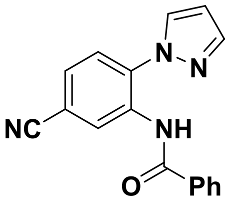


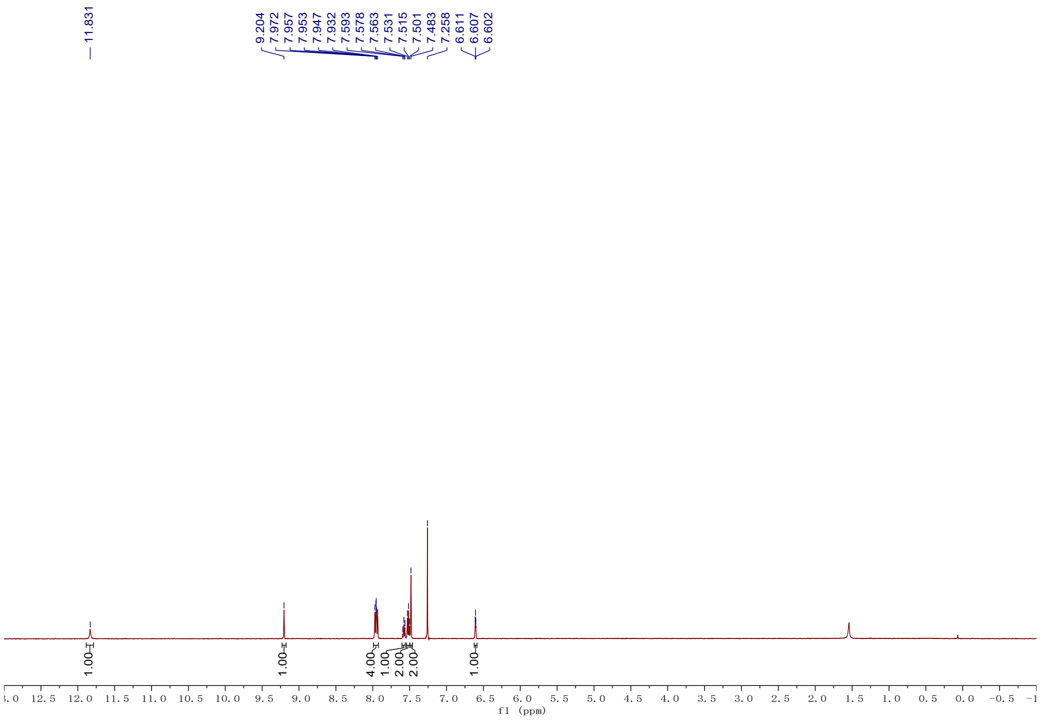

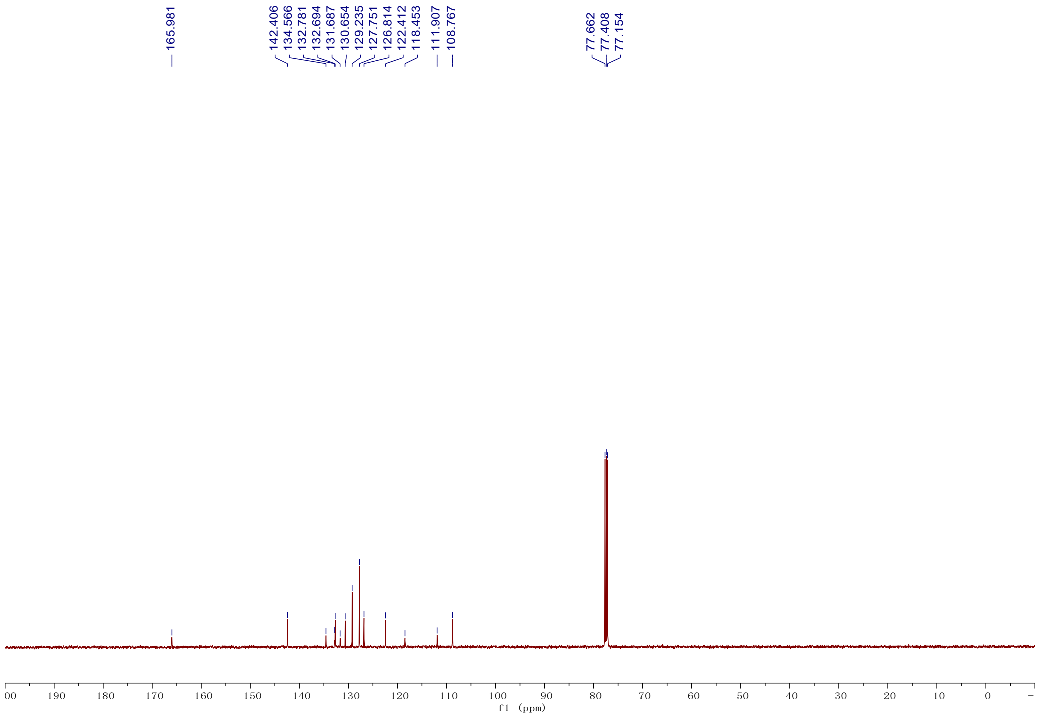

## $^1H$ NMR and $^{13}C$ NMR Spectra of Compound 3oa

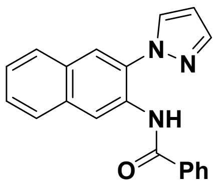


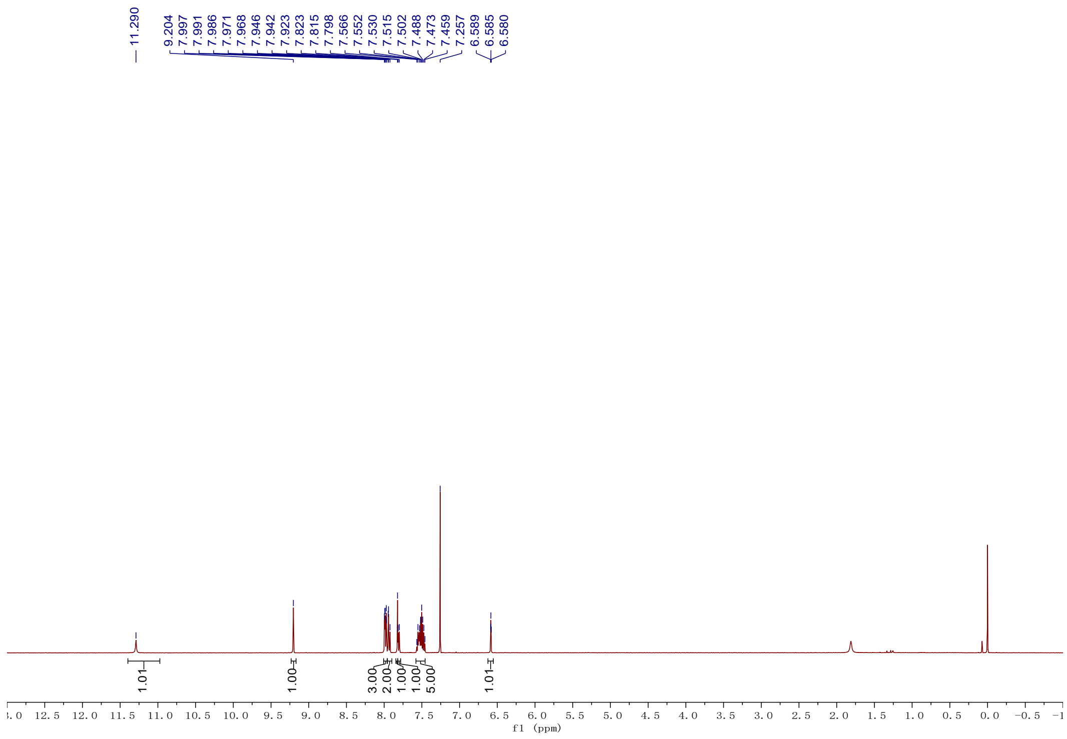


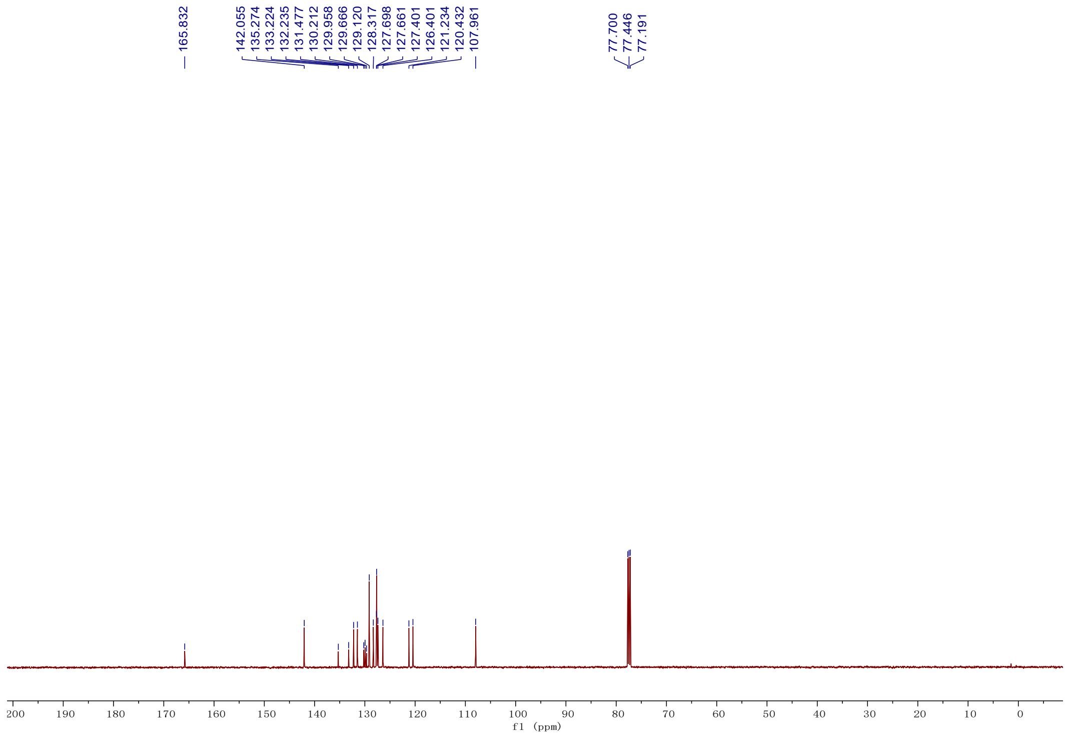

## $^{1}H$ NMR and $^{13}C$ NMR Spectra of Compound 3ab

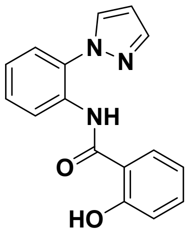


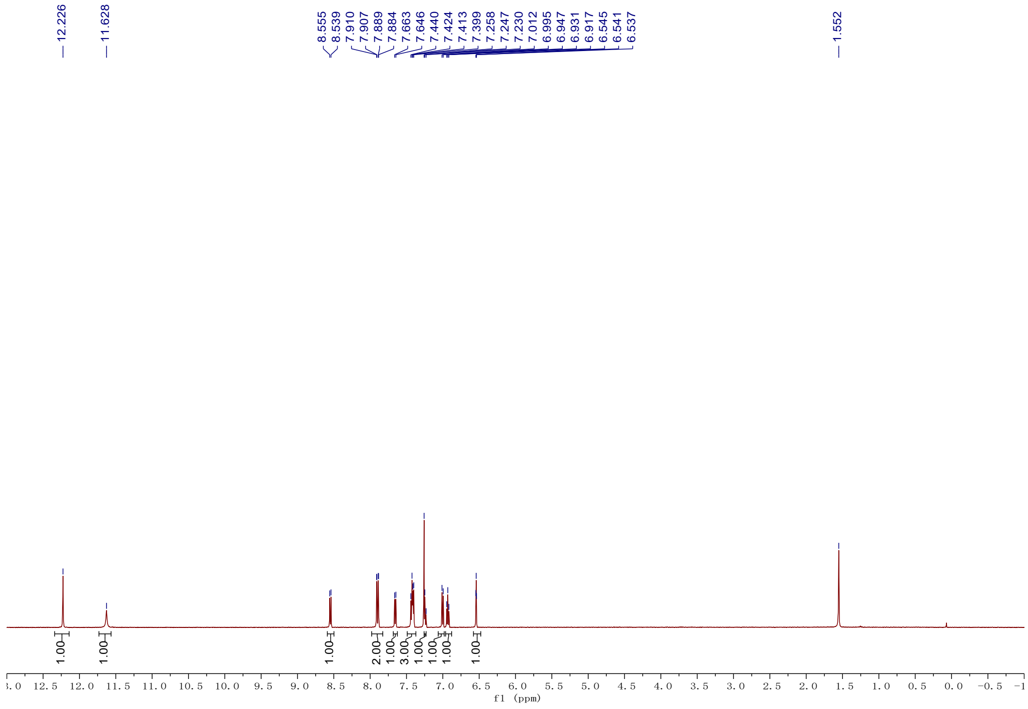


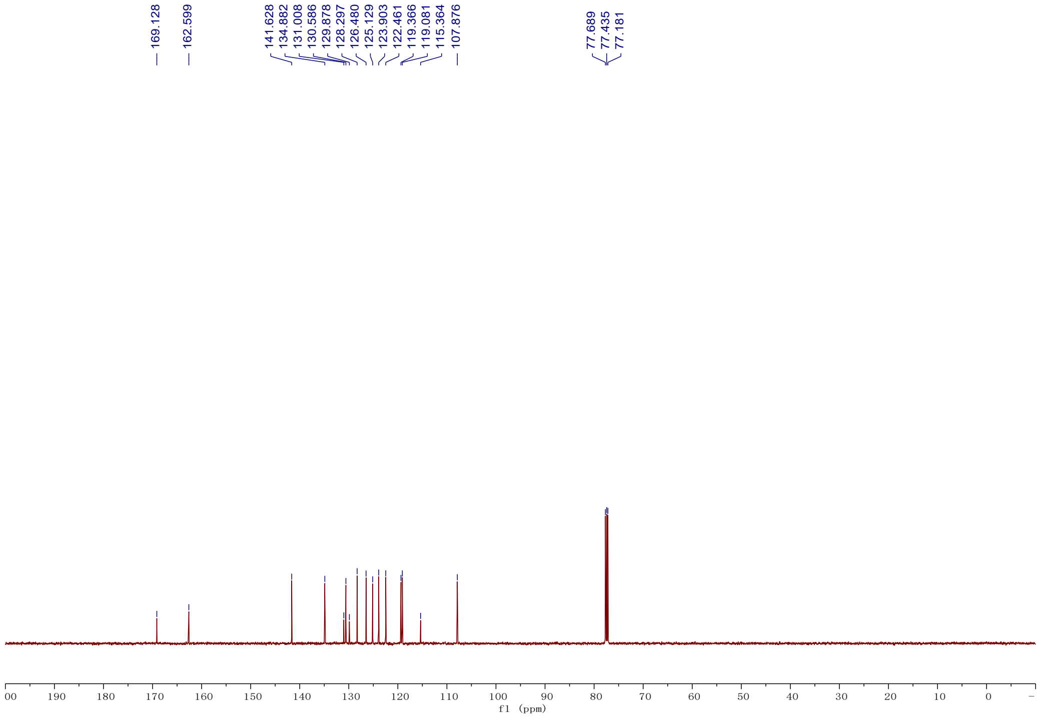

## $^{1}$H NMR and $^{13}$C NMR Spectra of Compound 3ac

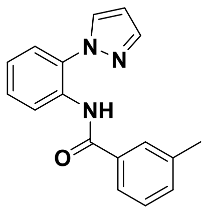


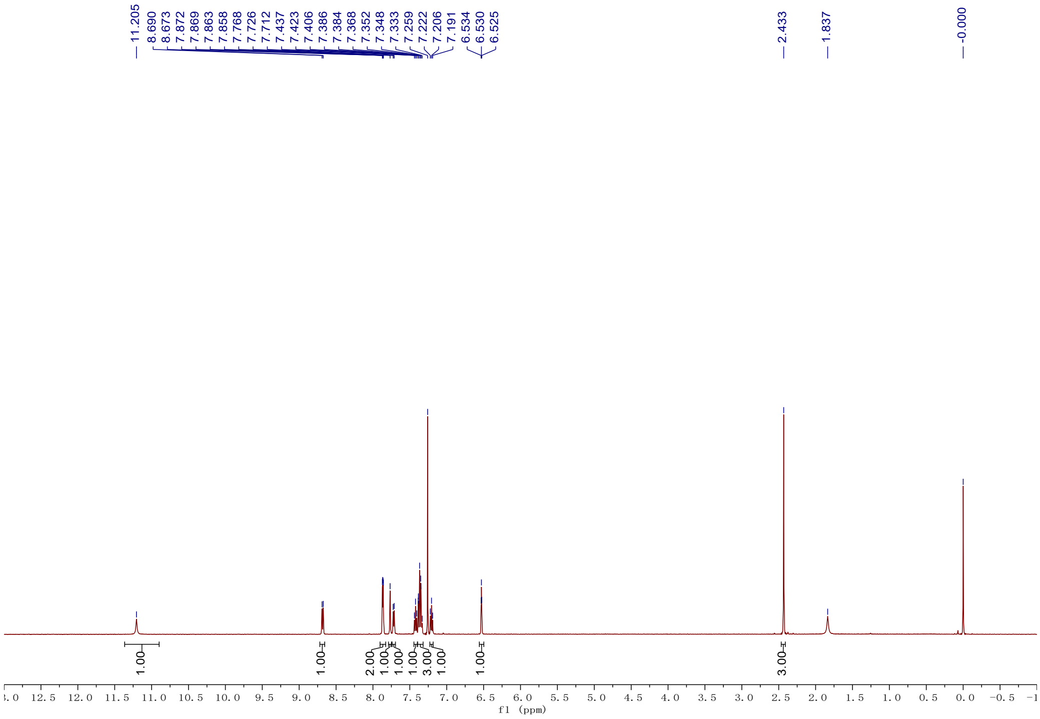


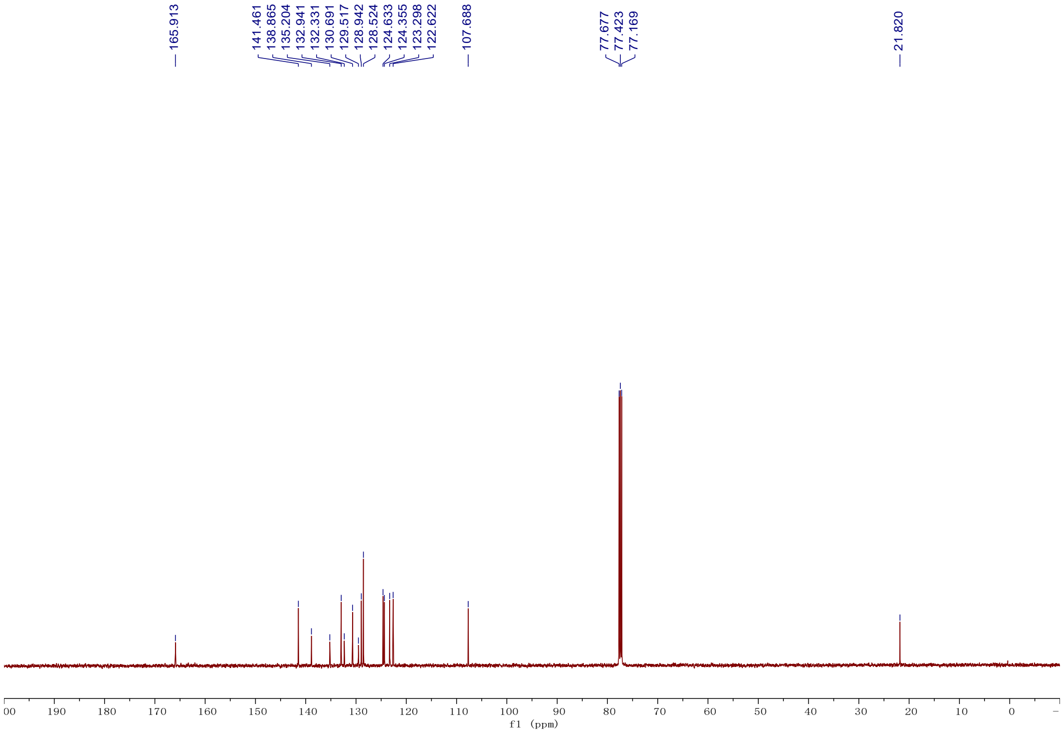

## $^{1}$H NMR and $^{13}$C NMR Spectra of Compound 3ad

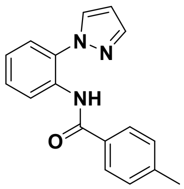


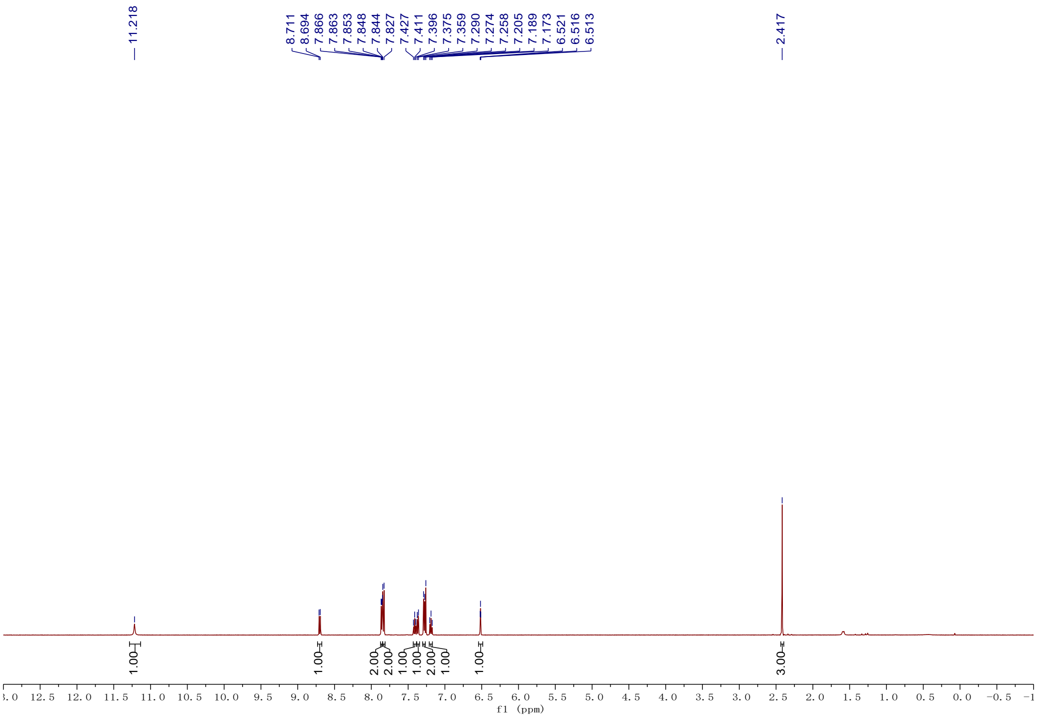


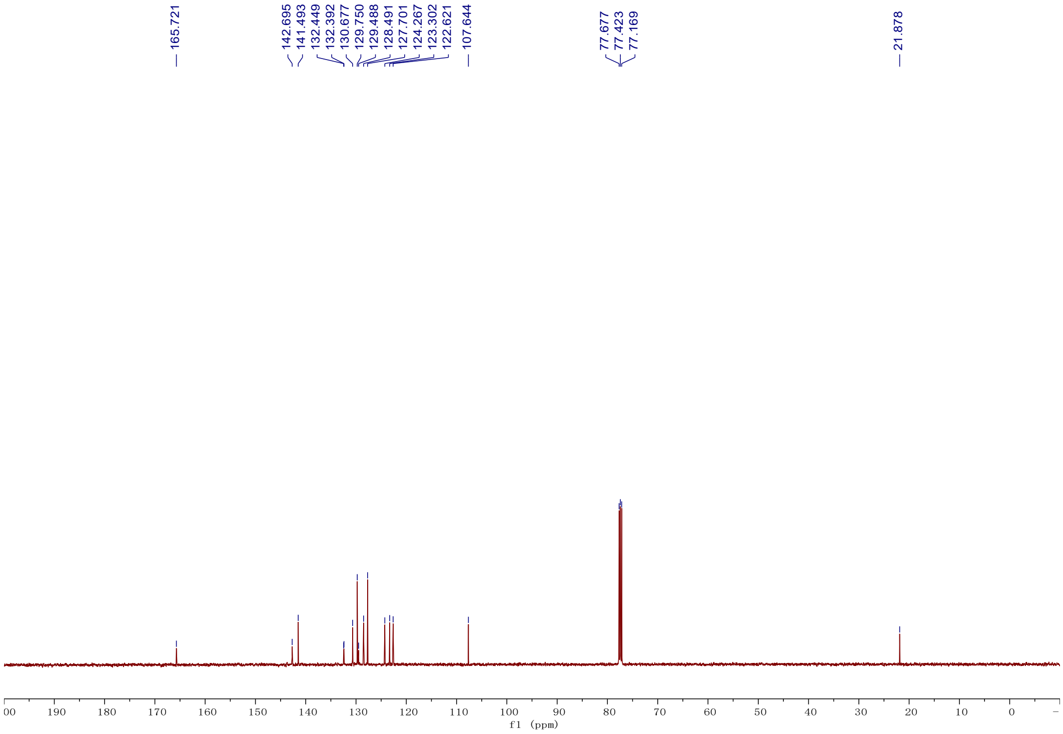

**$^{1}H$ NMR and $^{13}C$ NMR Spectra of Compound 3ae**

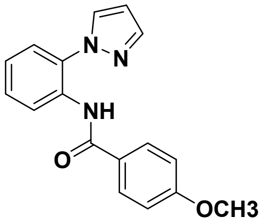


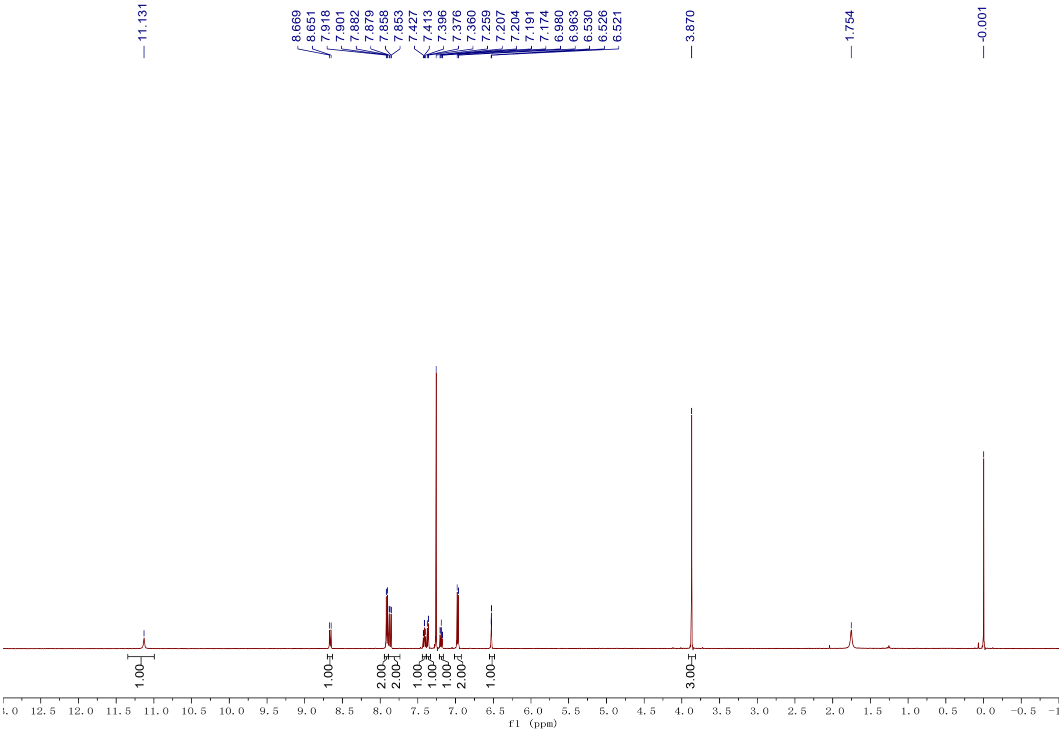


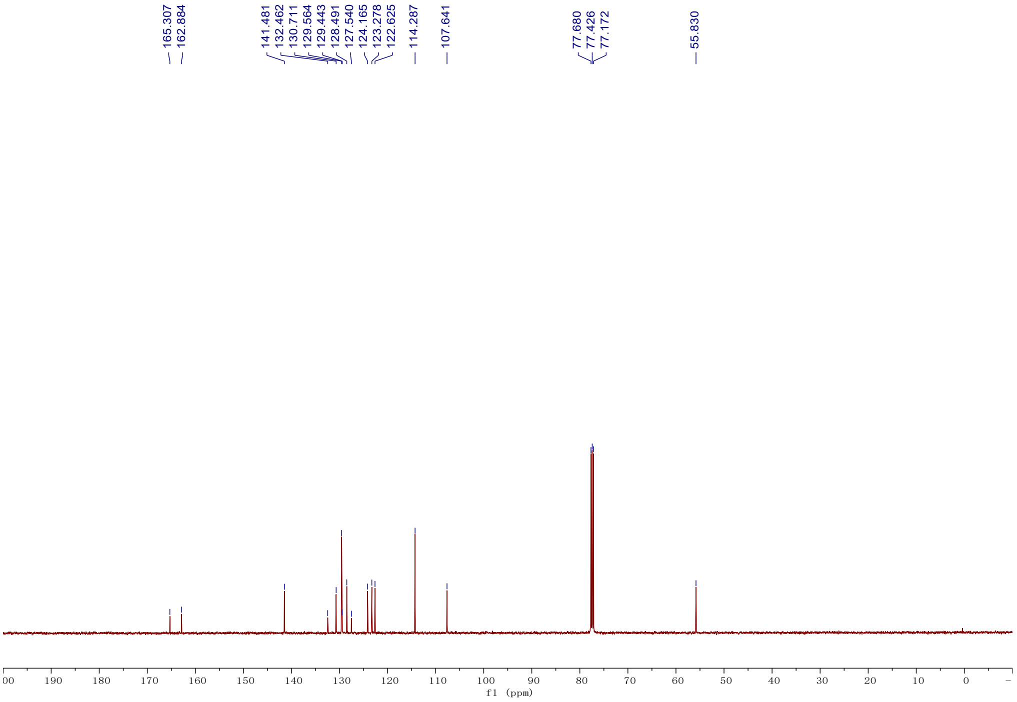

## $^{1}$H NMR and $^{13}$C NMR Spectra of Compound 3af

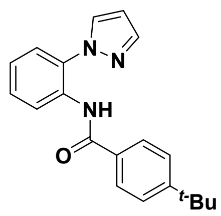


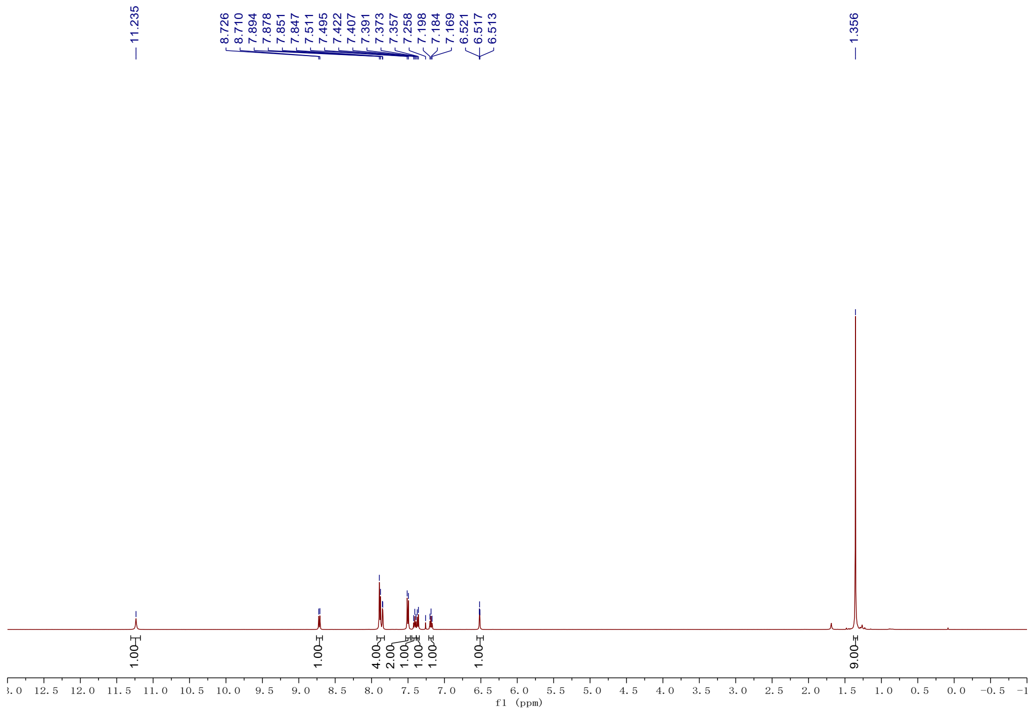


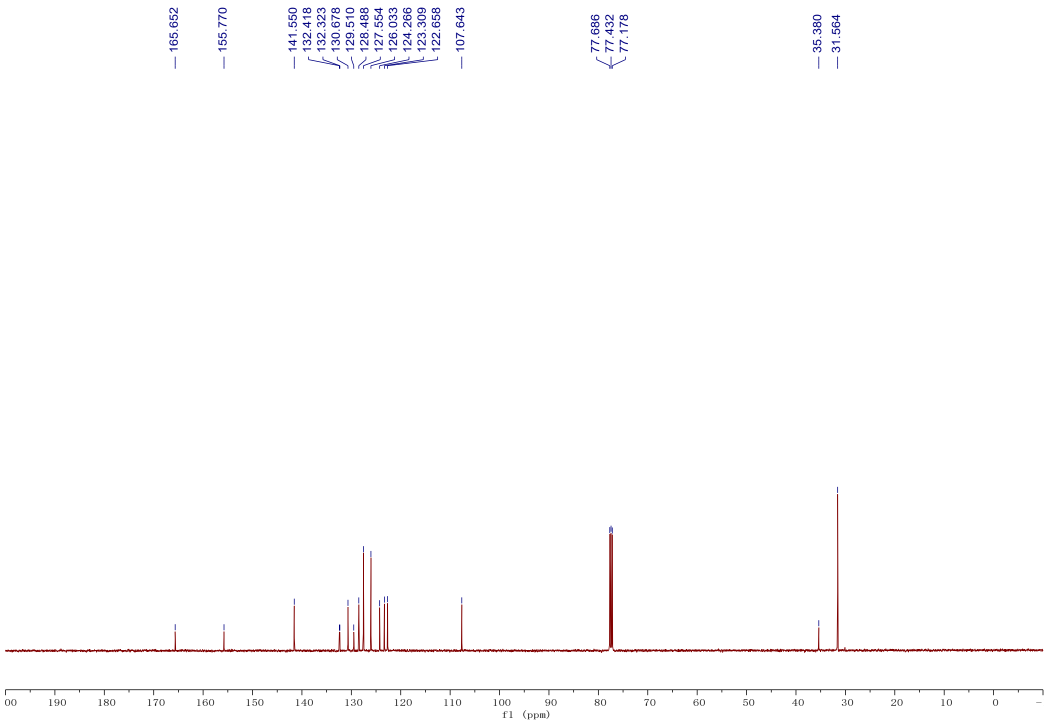

## $^{1}$H NMR and $^{13}$C NMR Spectra of Compound 3ag

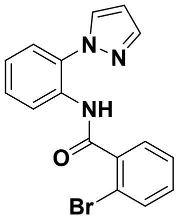


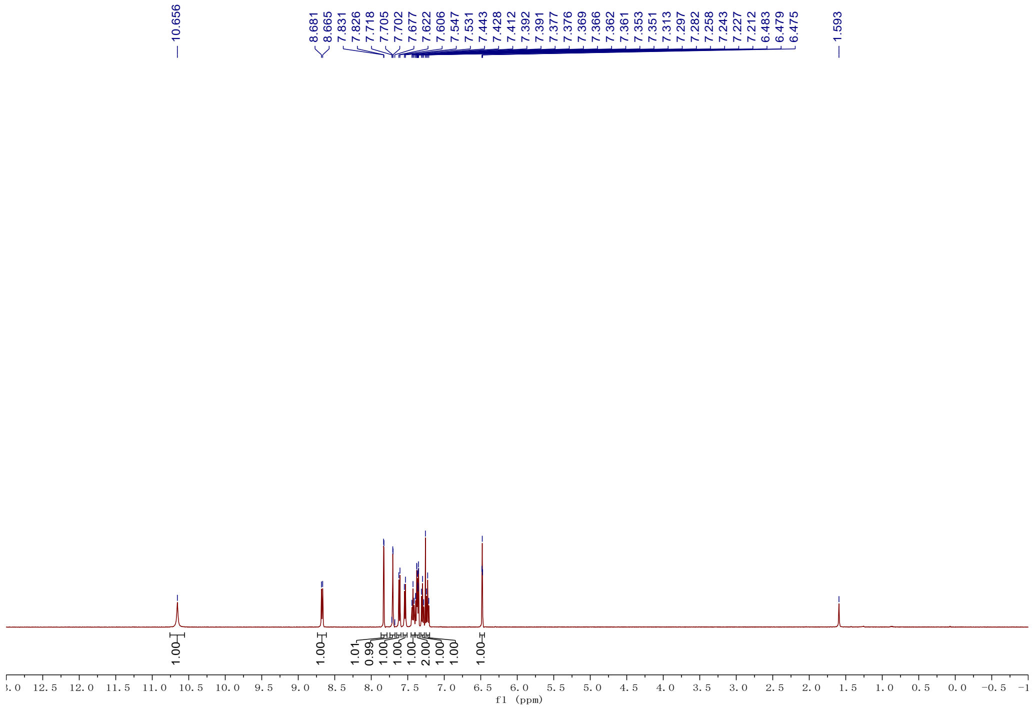


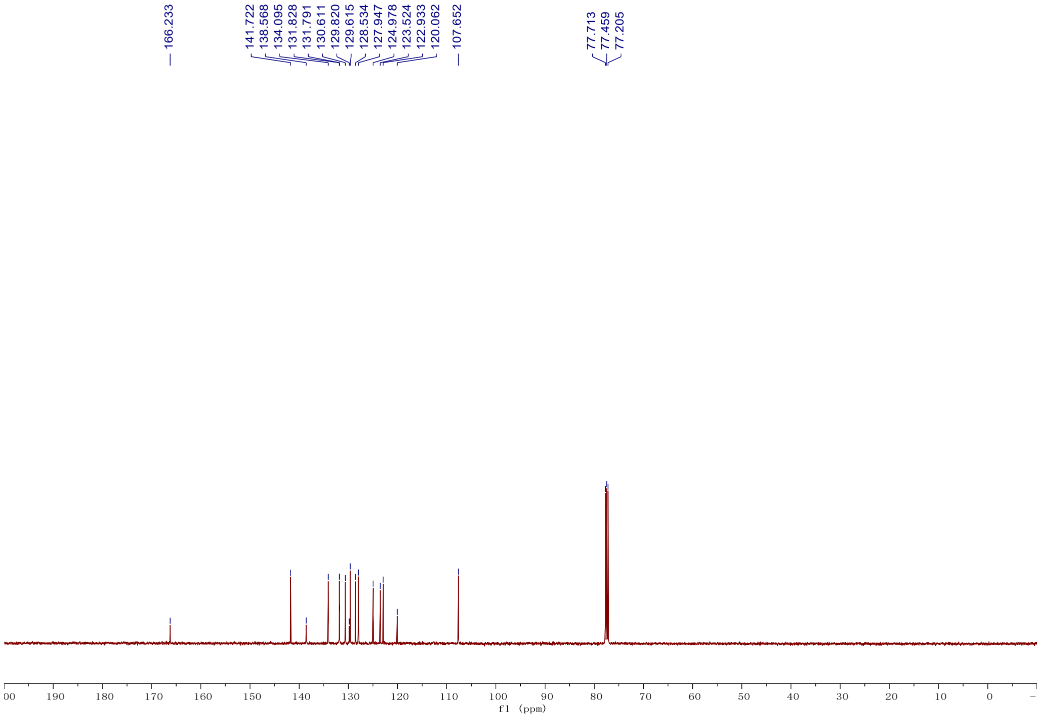

## $^{1}$H NMR and $^{13}$C NMR Spectra of Compound 3ah

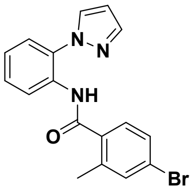


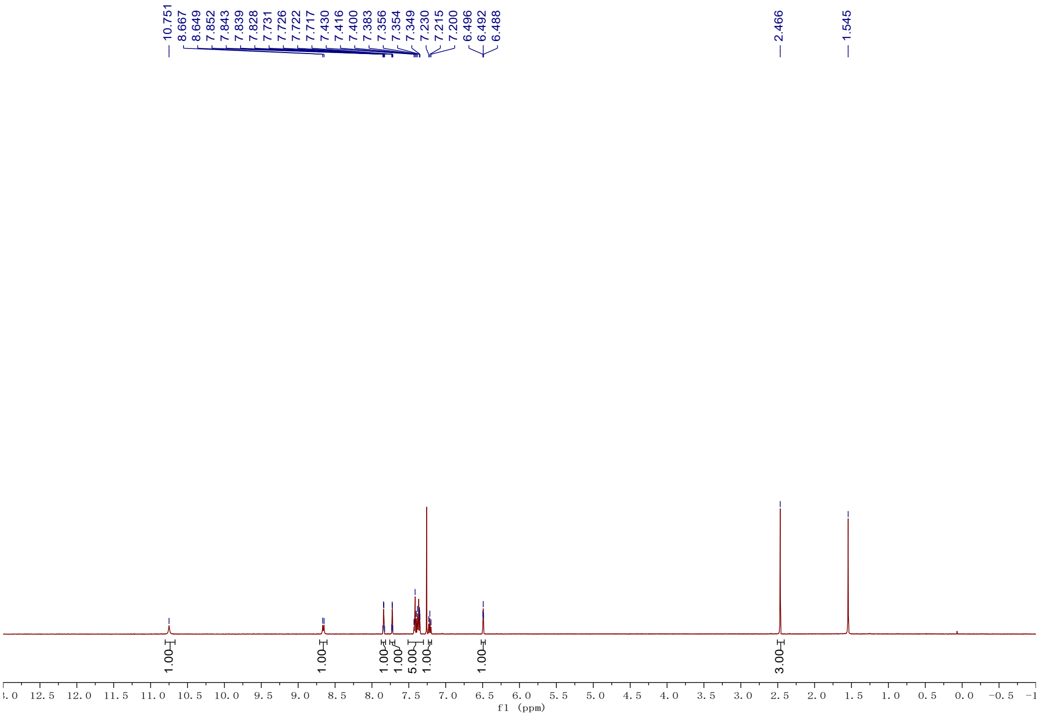


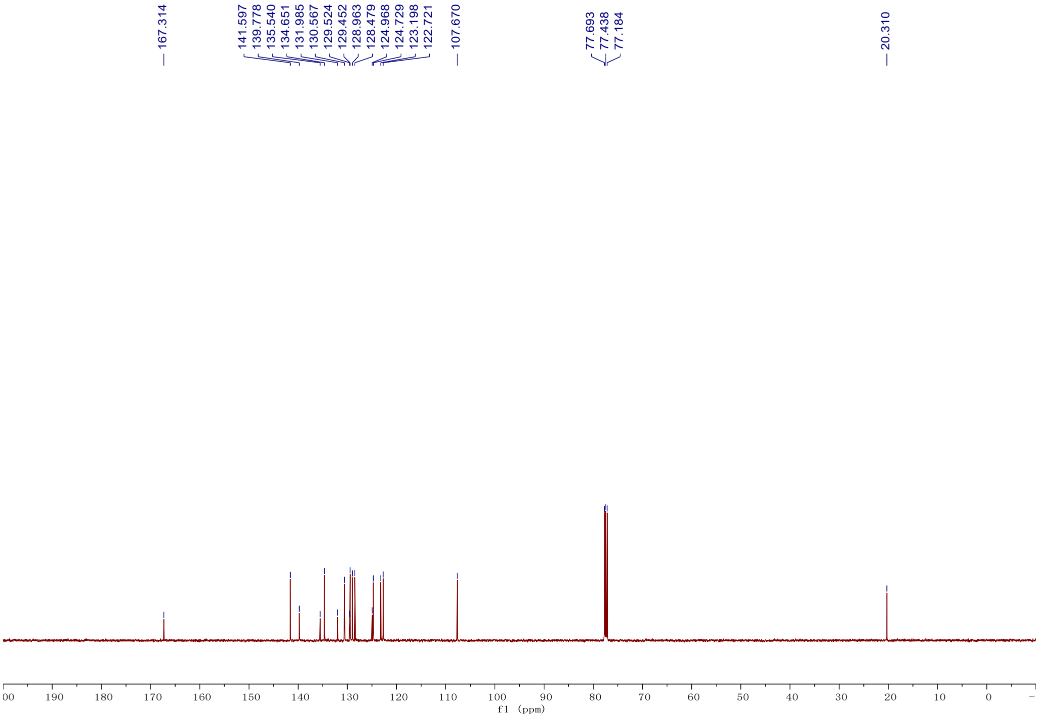

## $^{1}$H NMR and $^{13}$C NMR Spectra of Compound 3ai

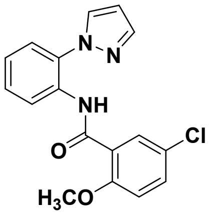


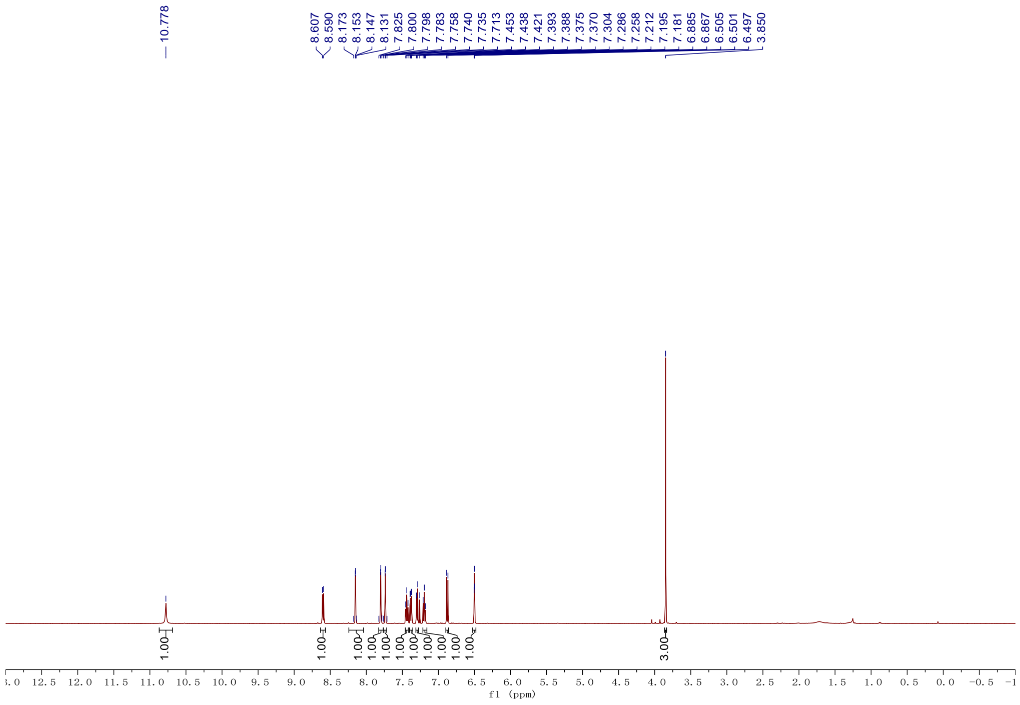


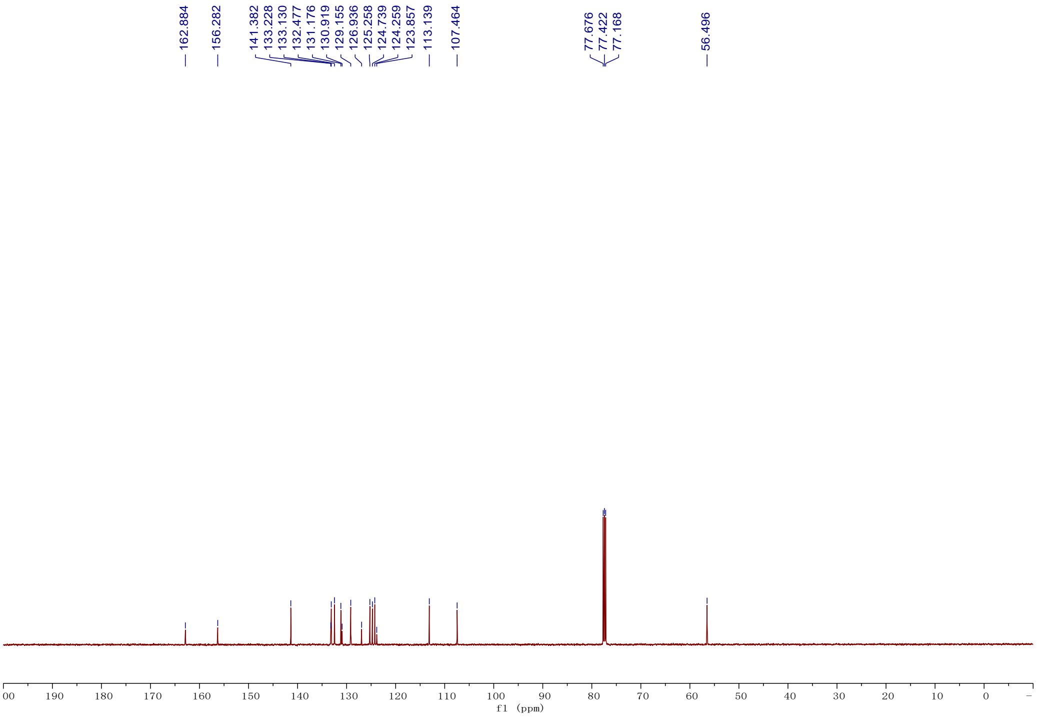

**$^{1}$H NMR and $^{13}$C NMR Spectra of Compound 3aj**

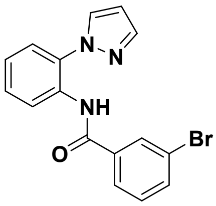

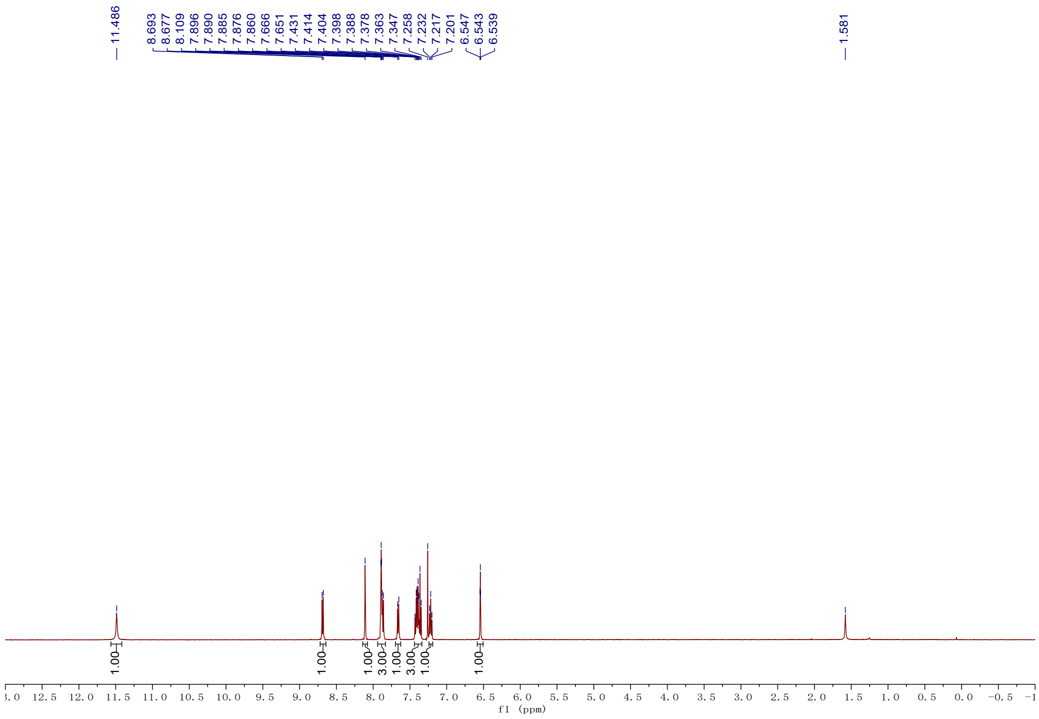


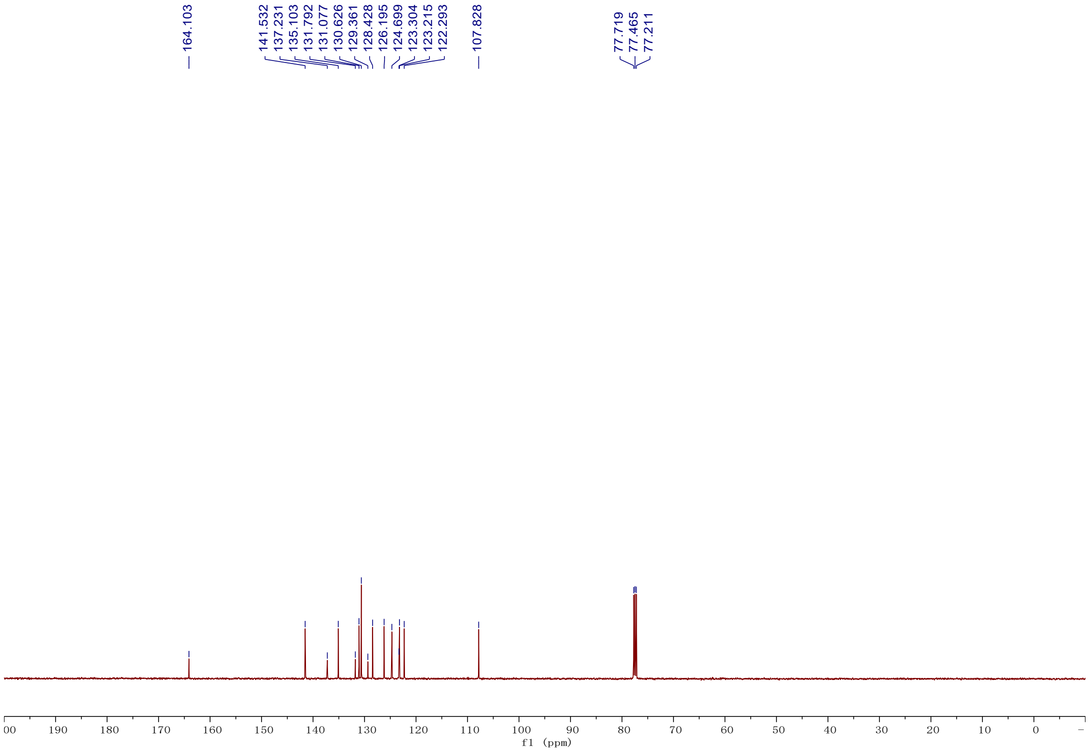

## $^{1}$H NMR and $^{13}$C NMR Spectra of Compound 3ak

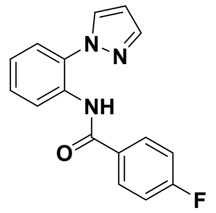

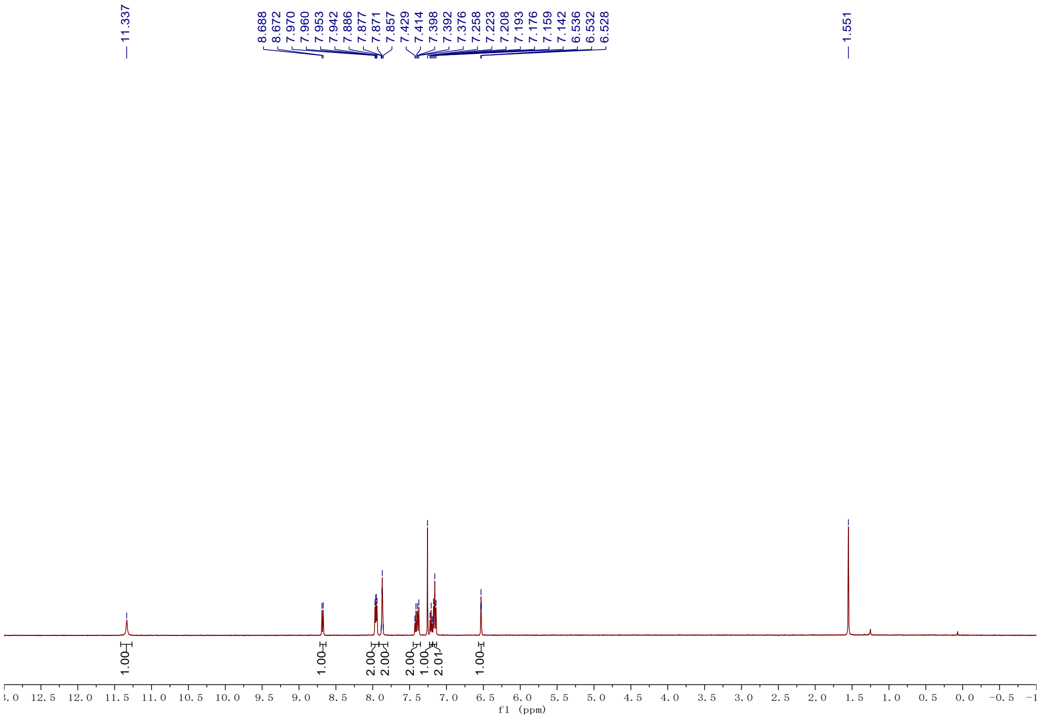

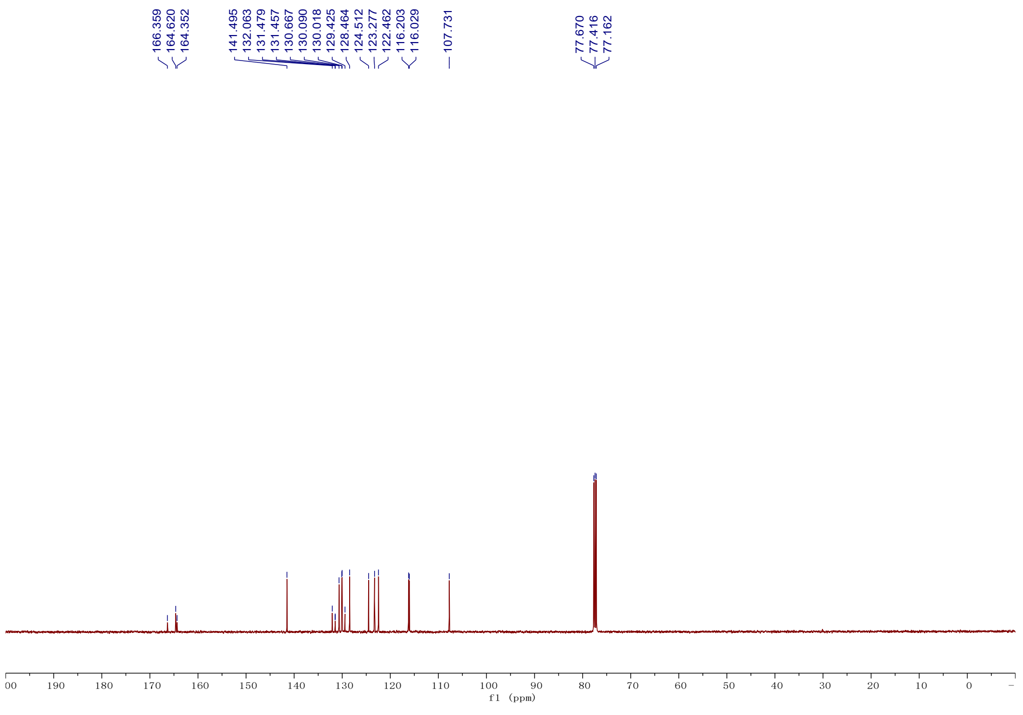

## $^{1}$H NMR and $^{13}$C NMR Spectra of Compound 3al

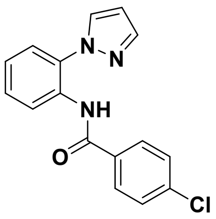

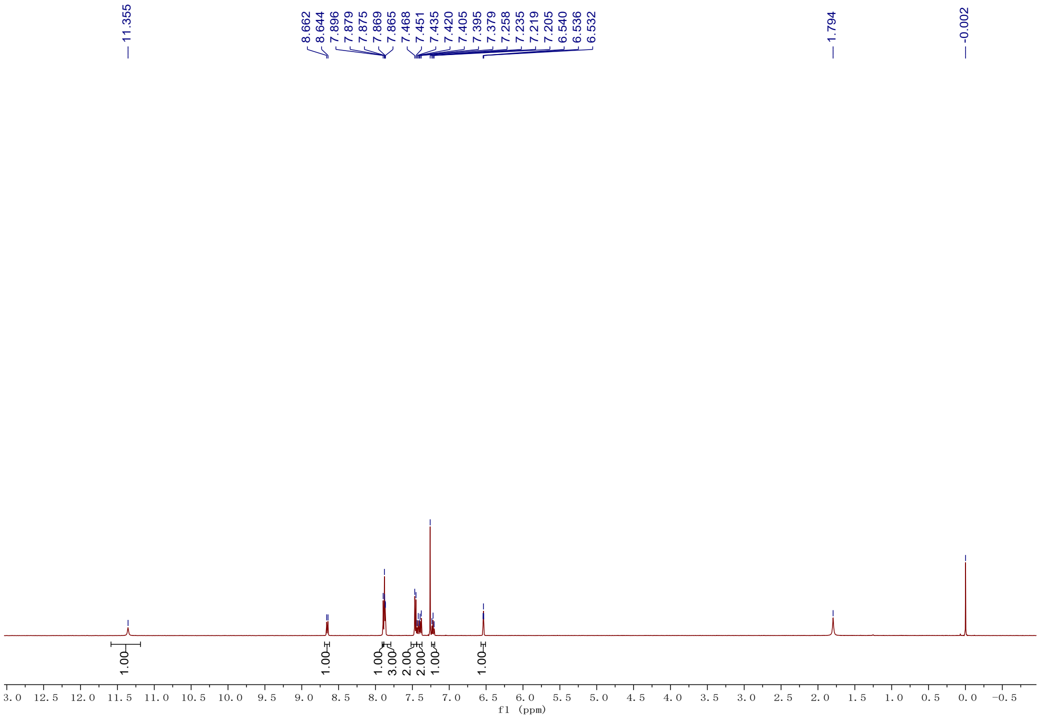


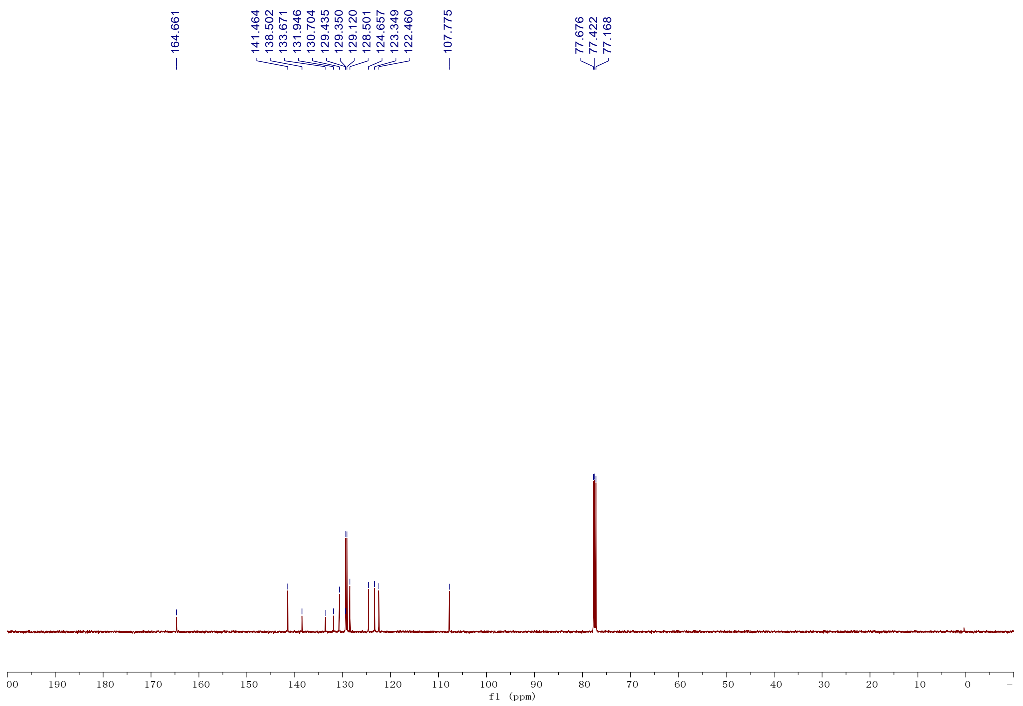

## $^{1}$H NMR and $^{13}$C NMR Spectra of Compound 3am

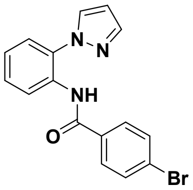


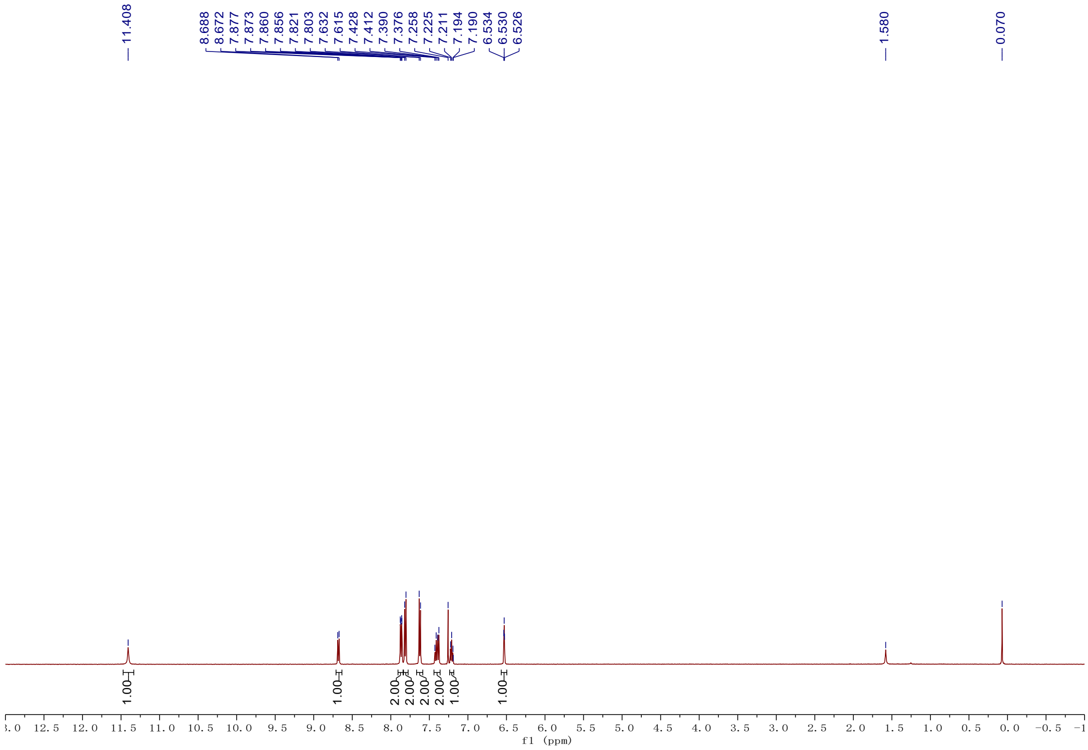


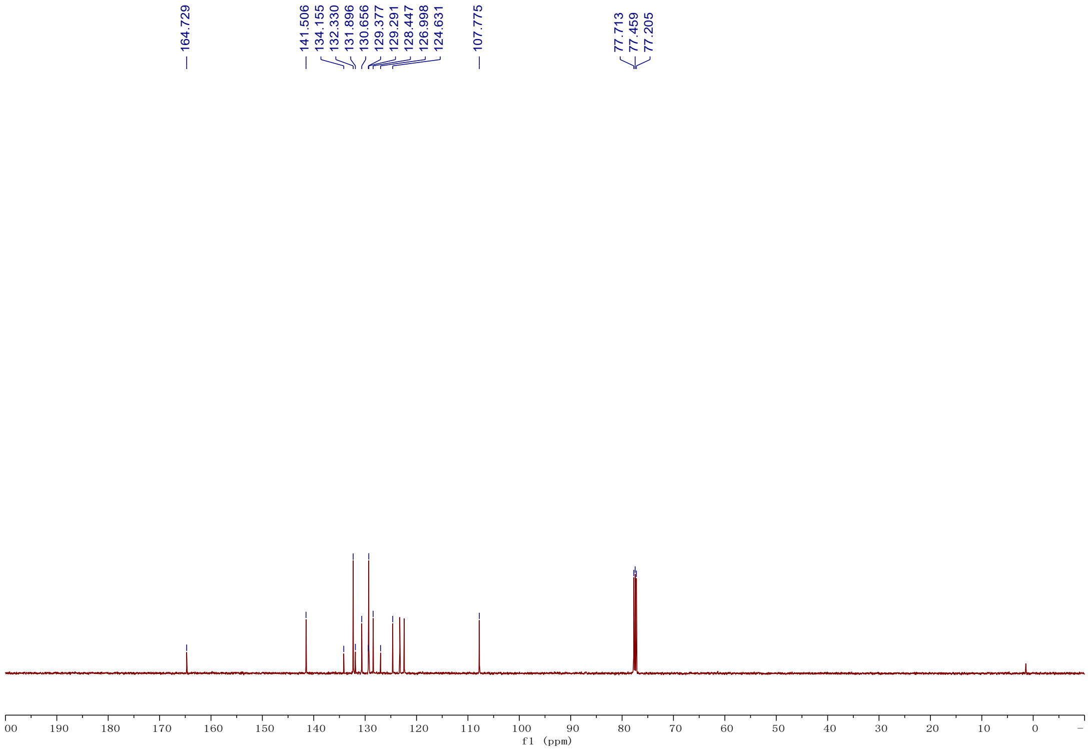

# $^{1}$H NMR and $^{13}$C NMR Spectra of Compound 3an

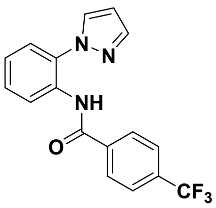

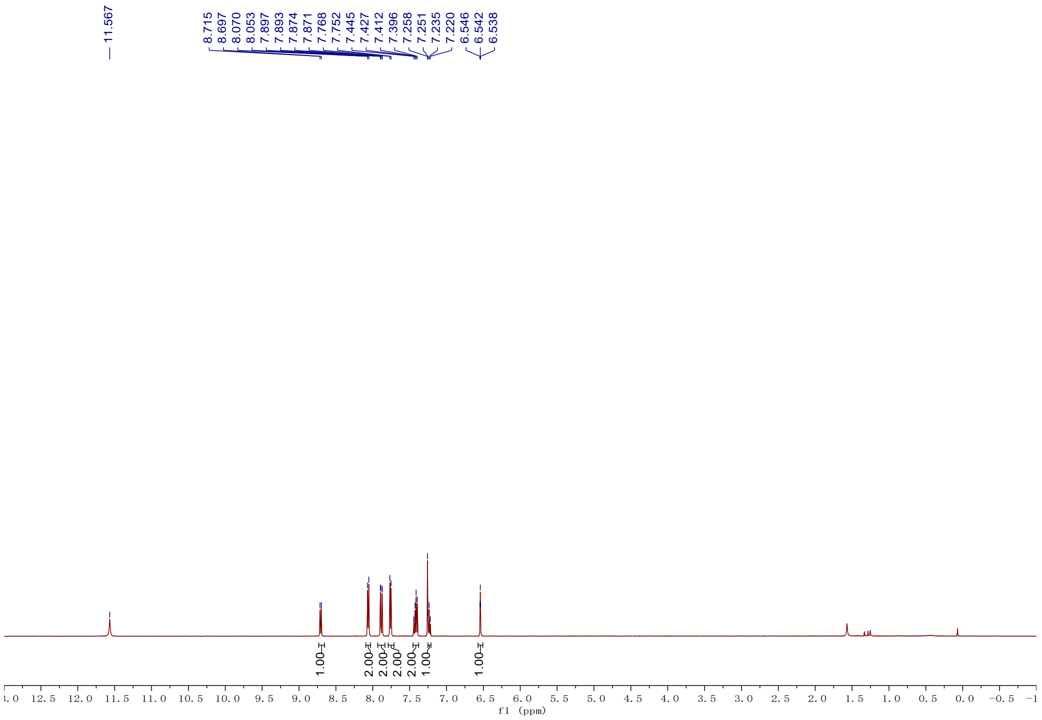

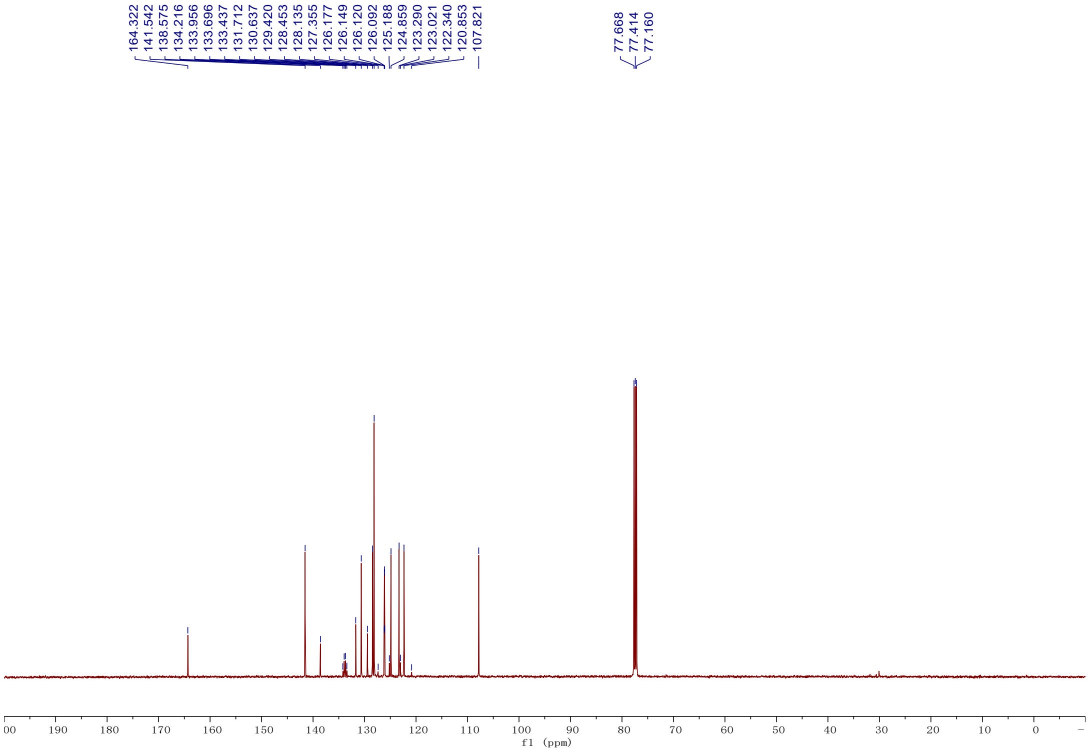

# $^{1}$H NMR and $^{13}$C NMR Spectra of Compound 3ao

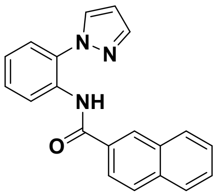


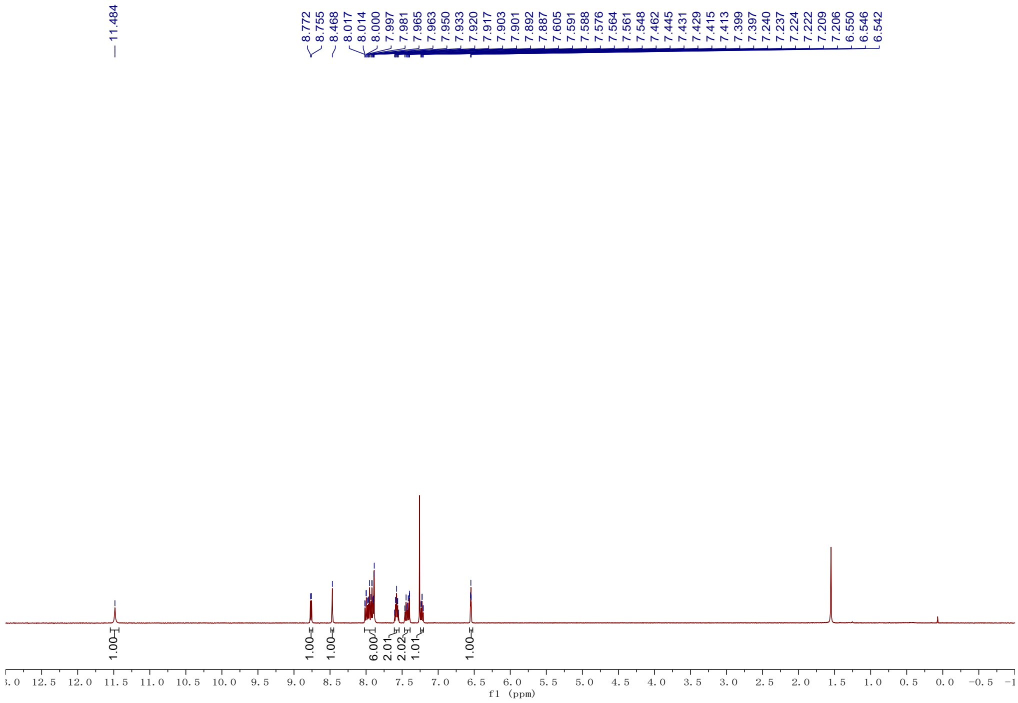


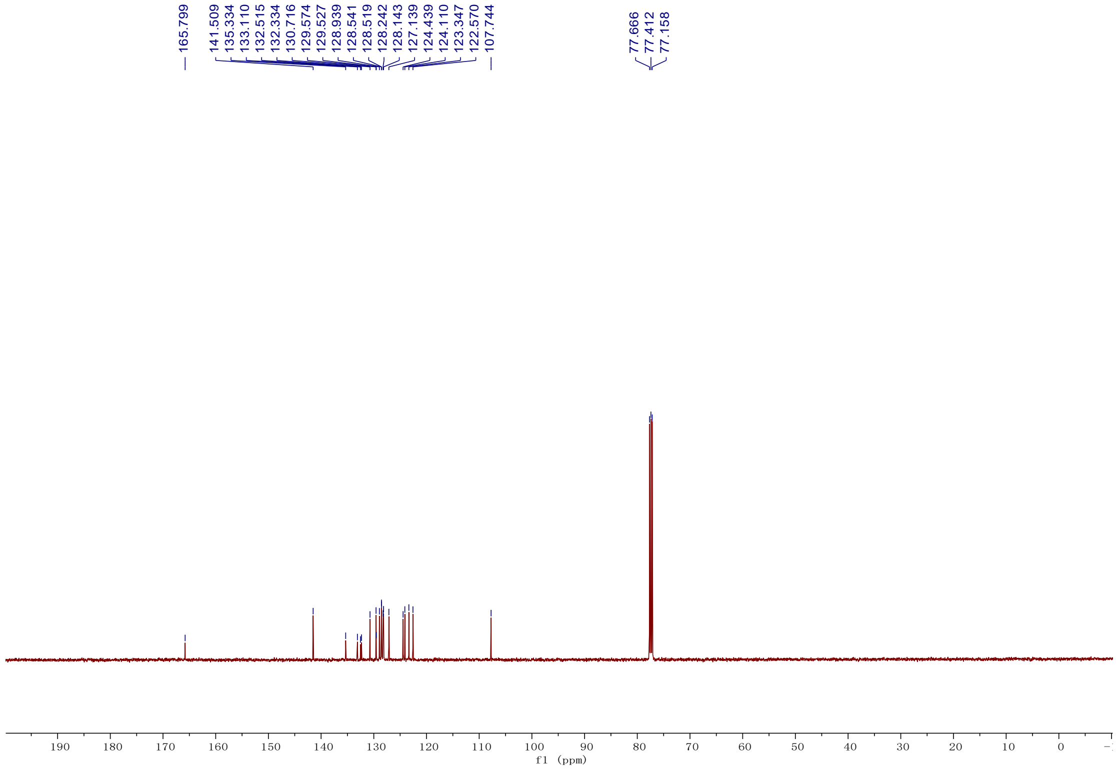

## $^{1}$H NMR and $^{13}$C NMR Spectra of Compound 3ap

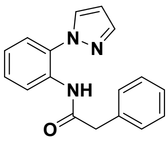


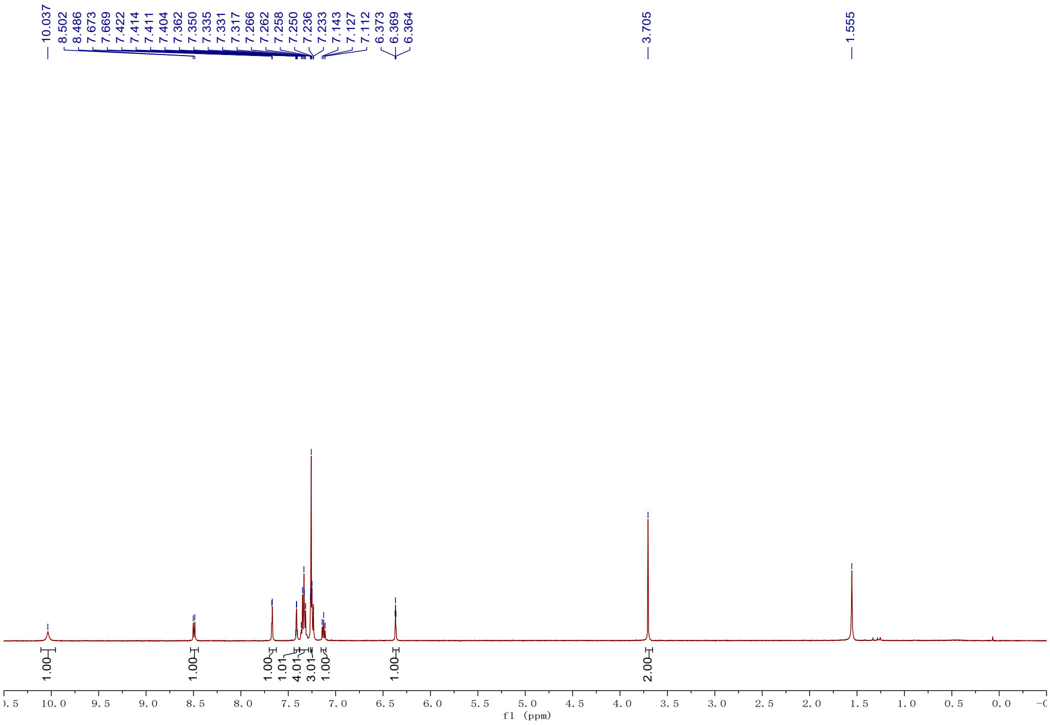


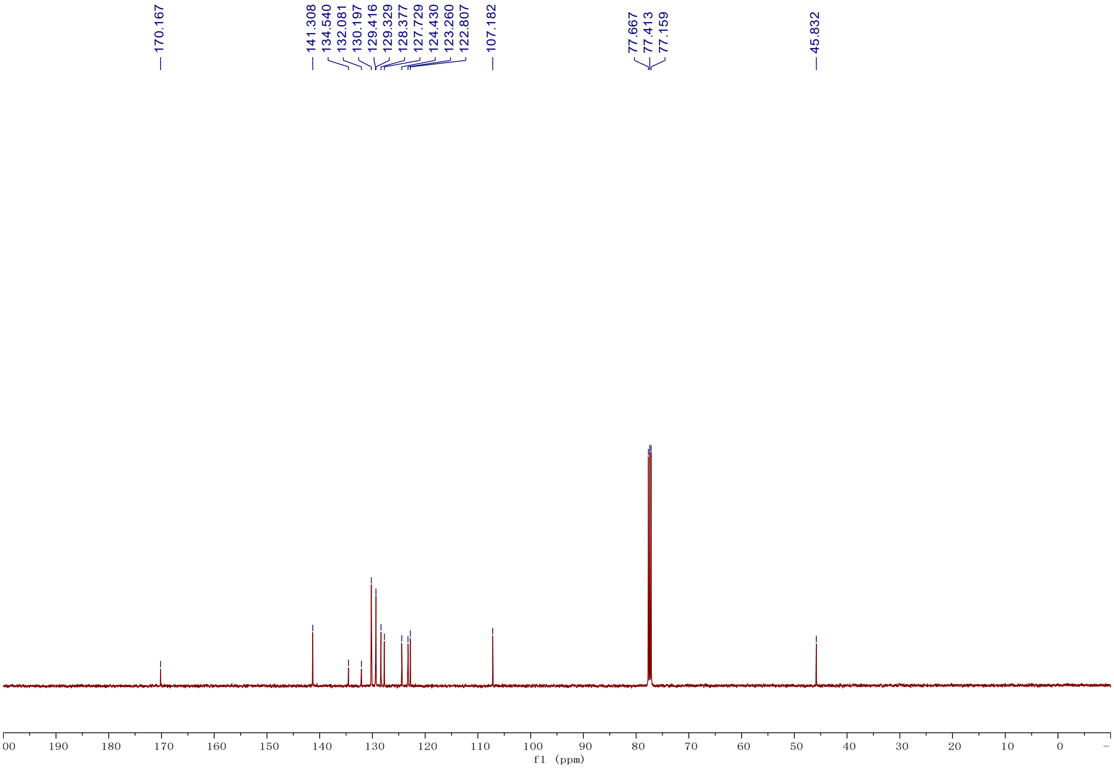

## $^{1}$H NMR and $^{13}$C NMR Spectra of Compound 9aa

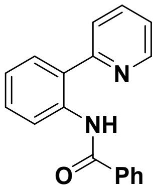

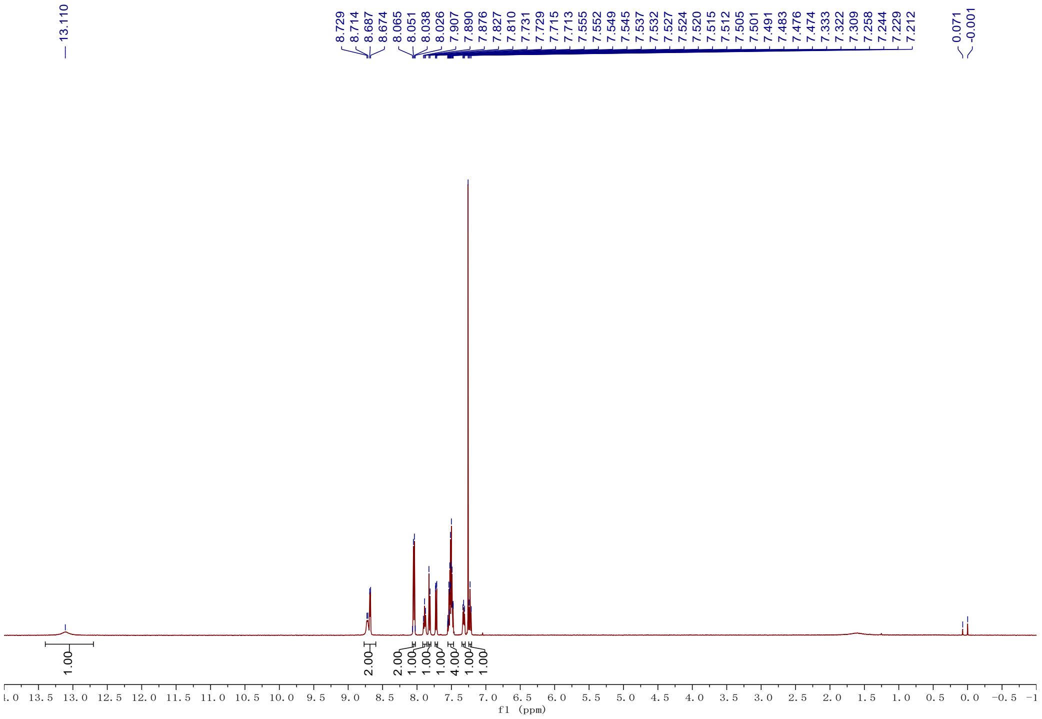

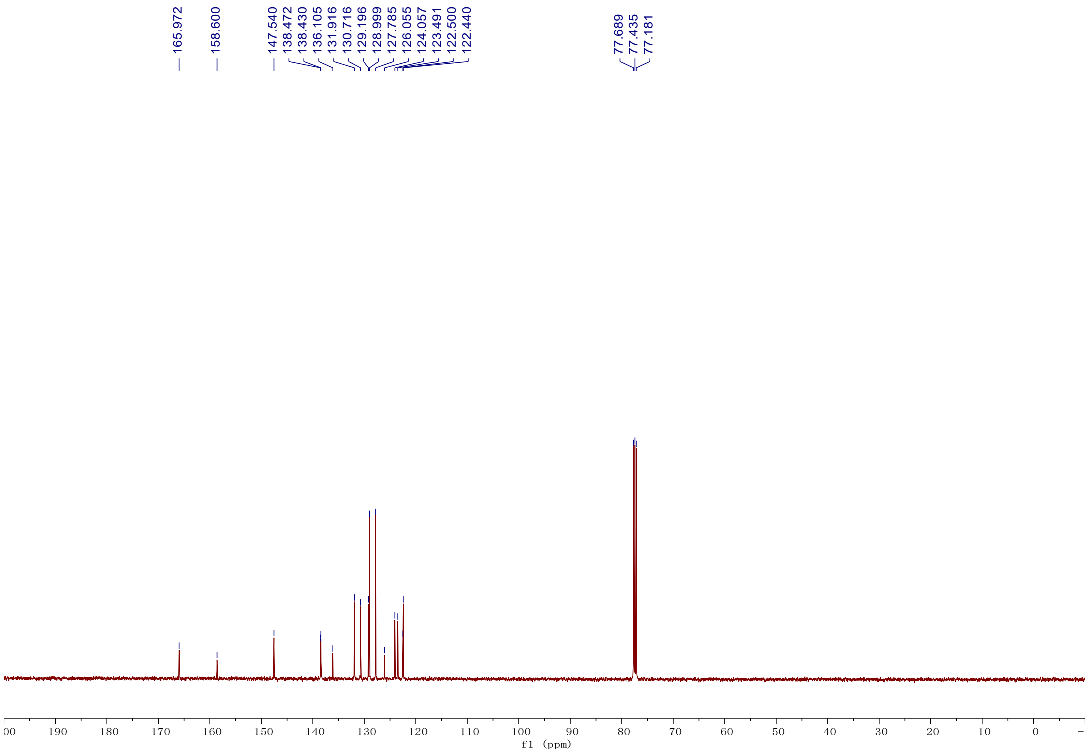

## $^{1}H$ NMR and $^{13}C$ NMR Spectra of Compound 10al

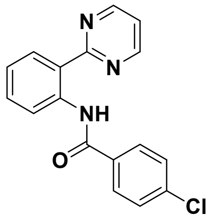


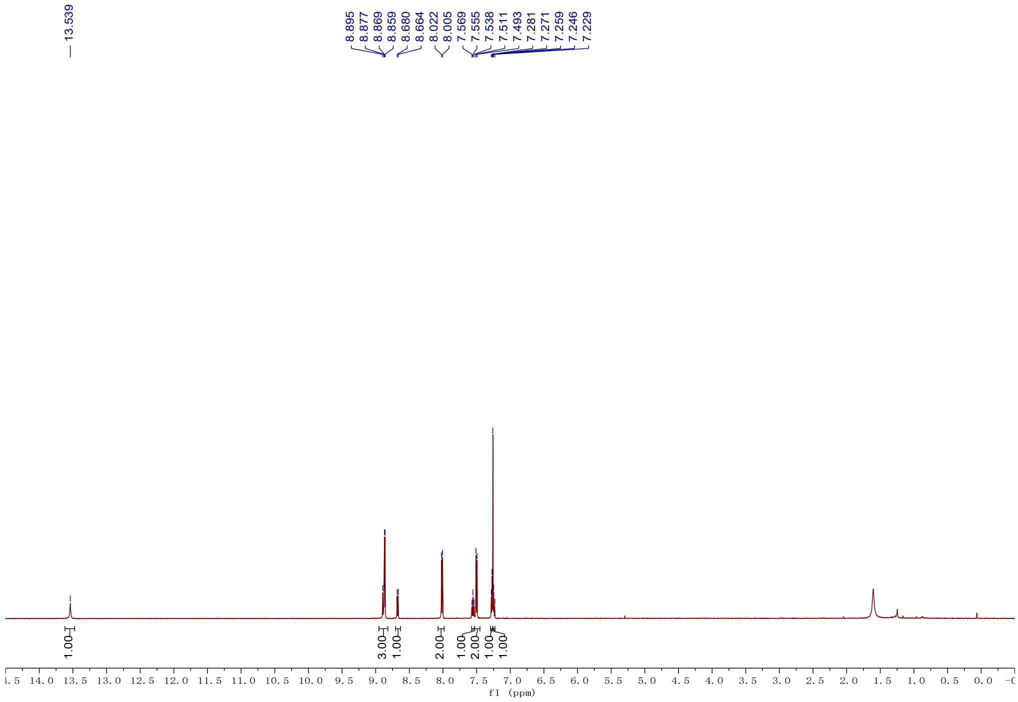


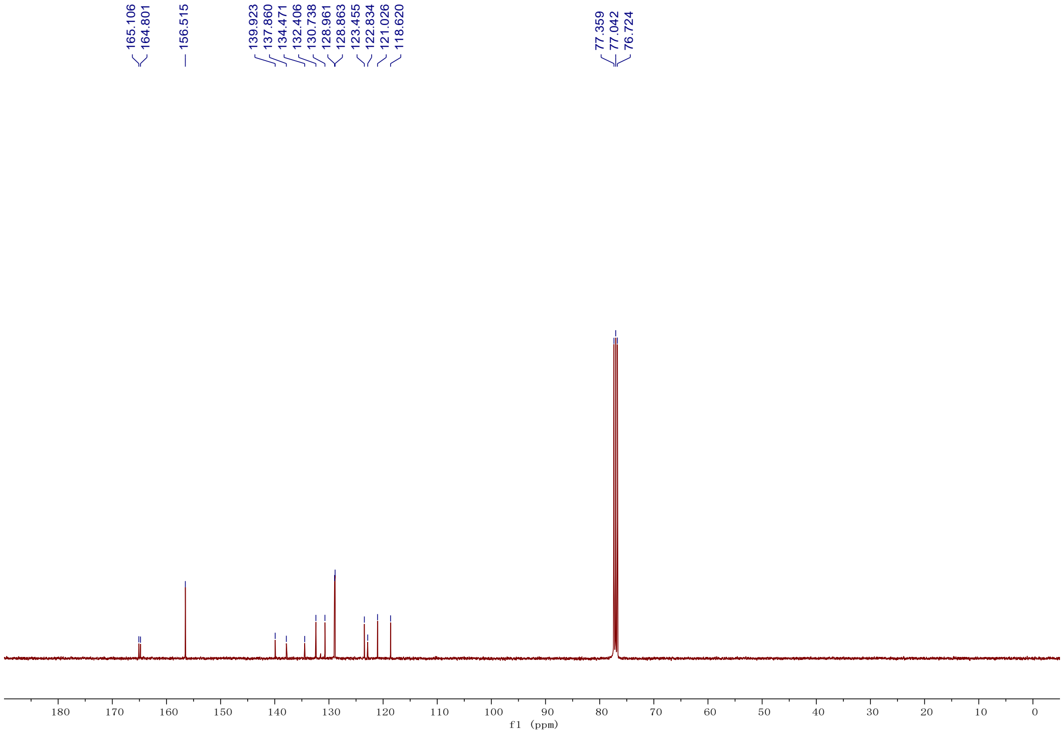

## $^{1}$H NMR and $^{13}$C NMR Spectra of Compound 11aa

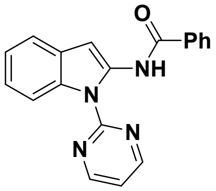


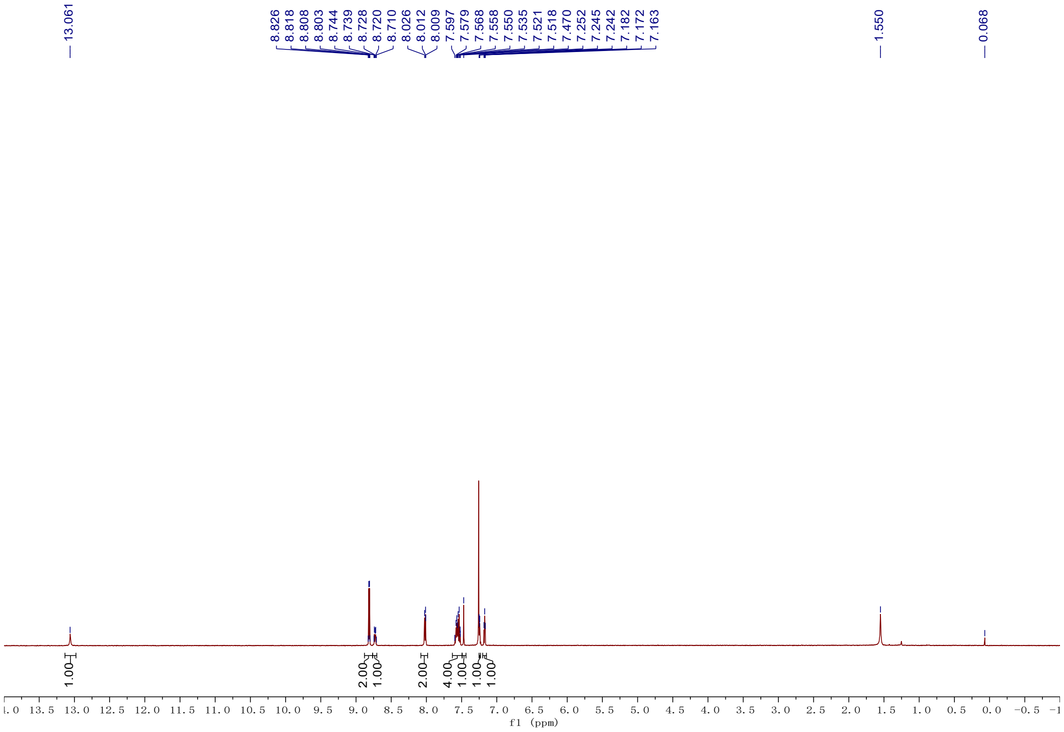


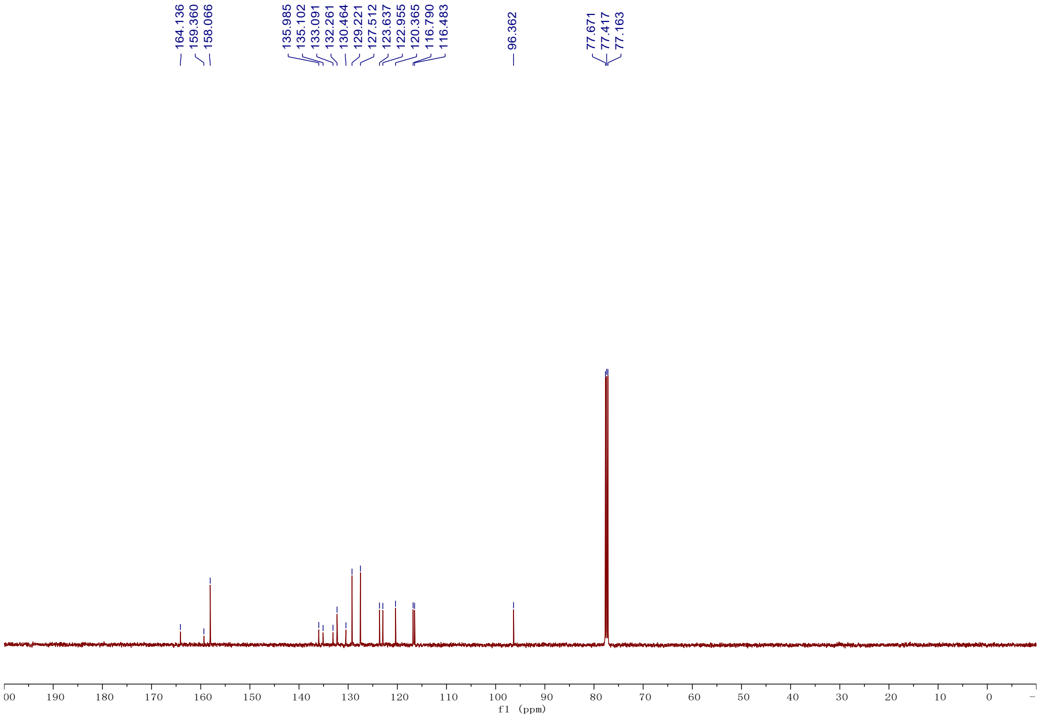

# $^{1}H$ NMR and $^{13}C$ NMR Spectra of Compound 11am

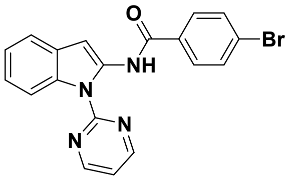


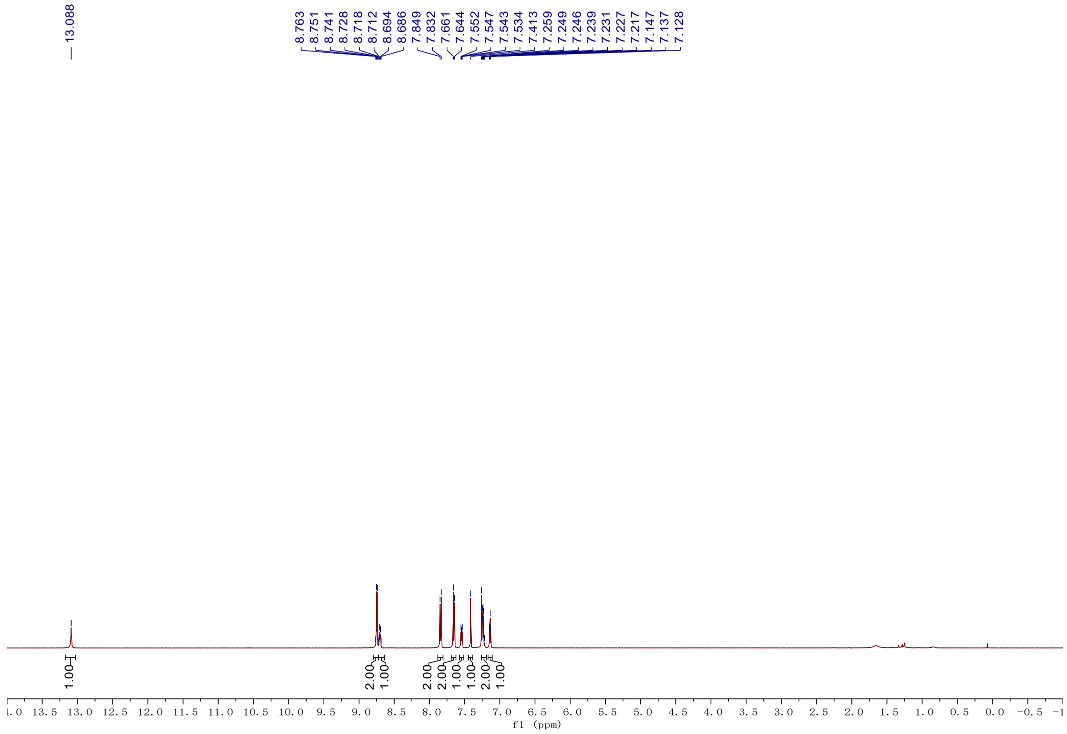


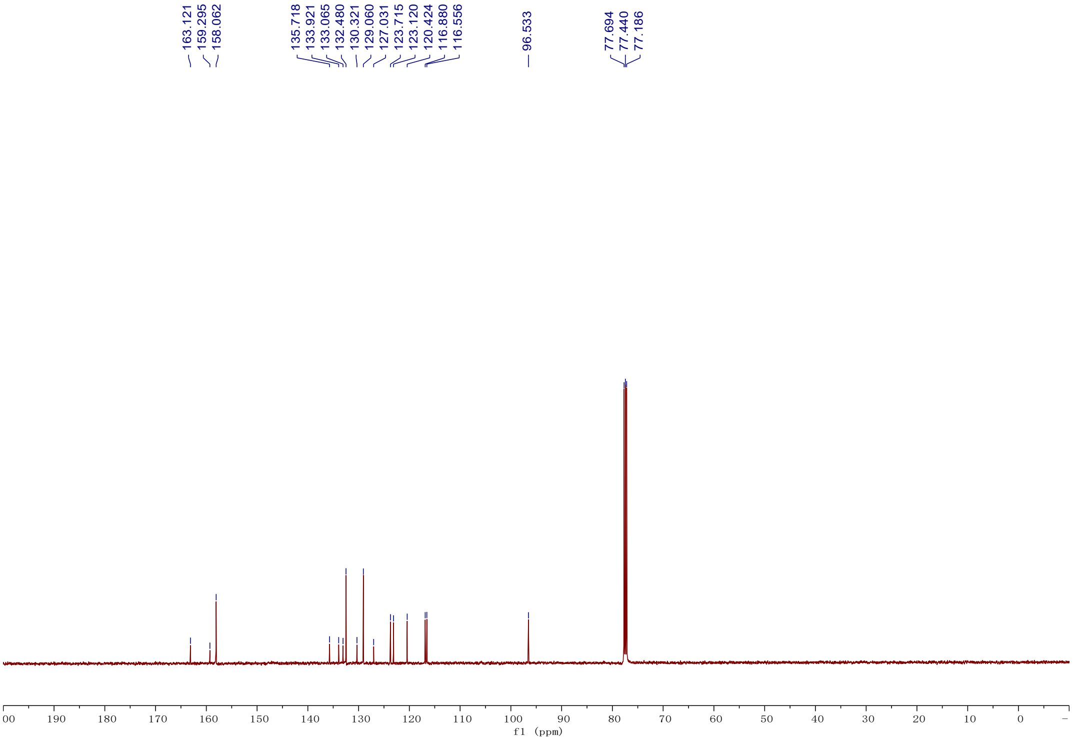

## $^{1}$H NMR and $^{13}$C NMR Spectra of Compound 3at

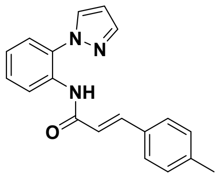

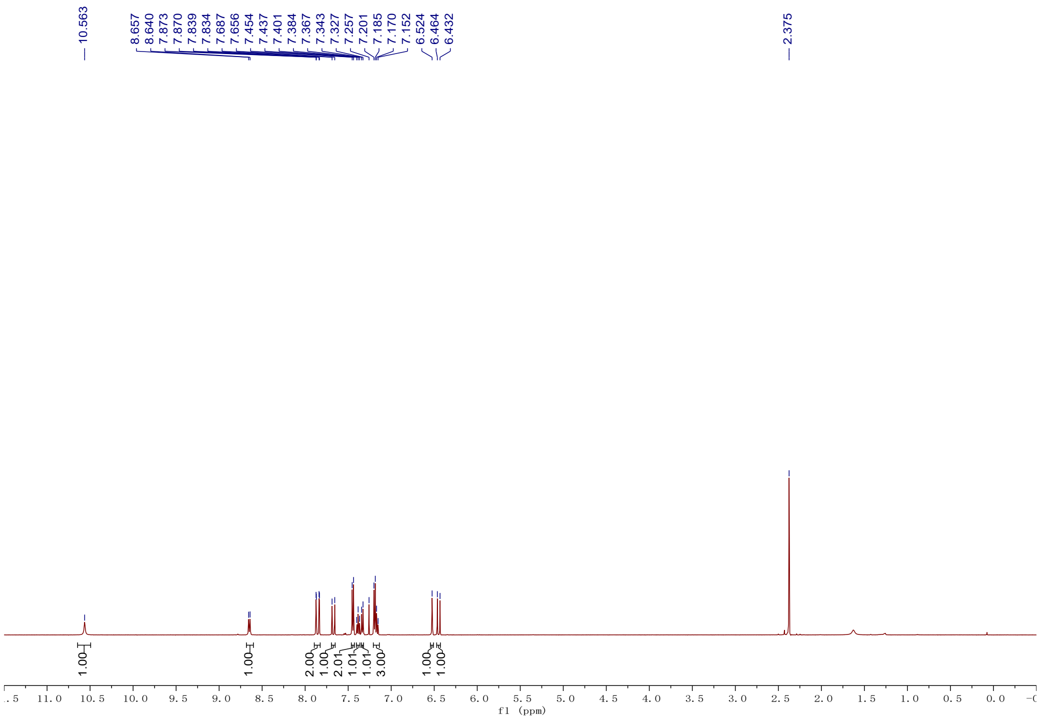

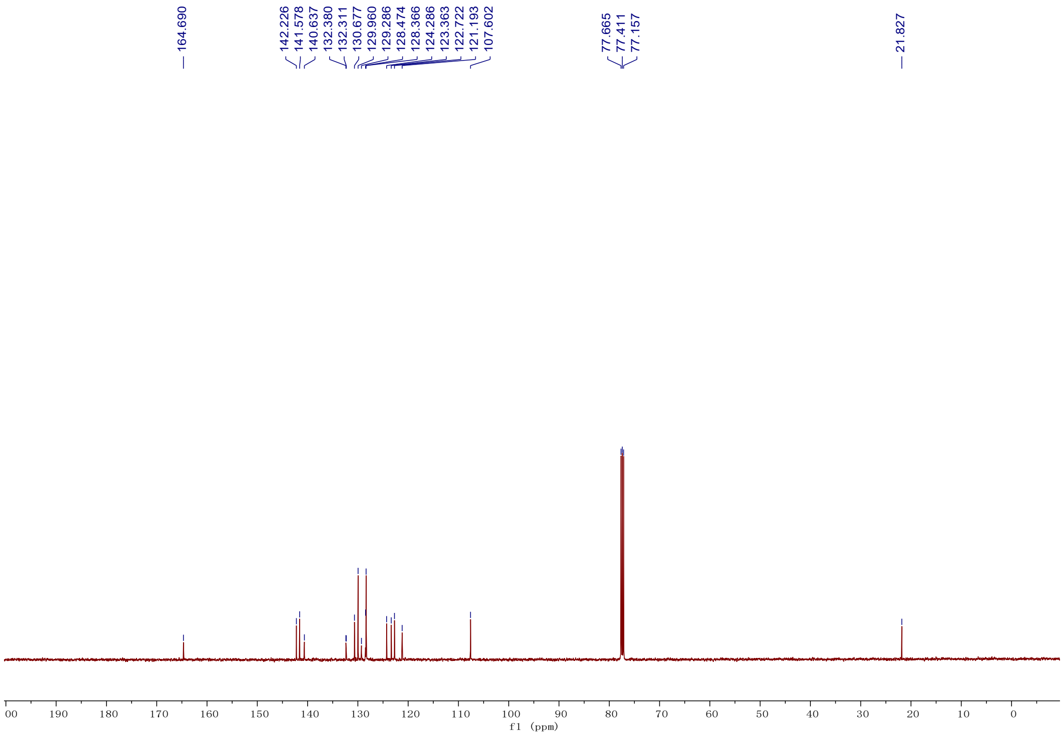

**$^{1}$H NMR and $^{13}$C NMR Spectra of Compound 3au**

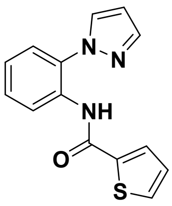


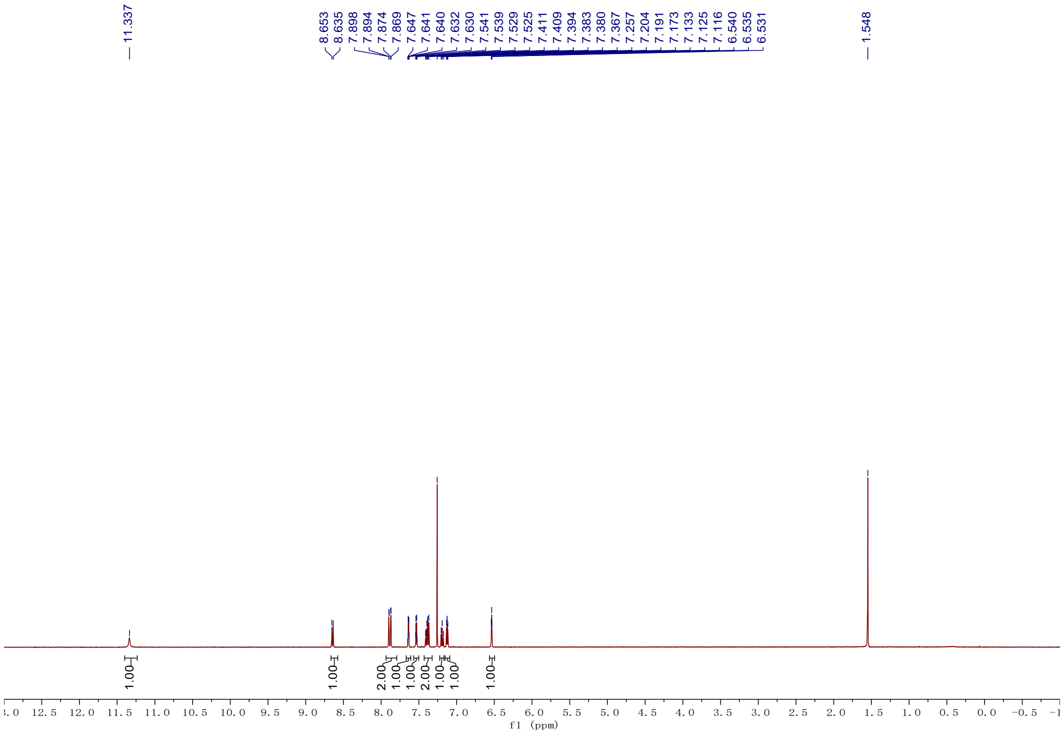


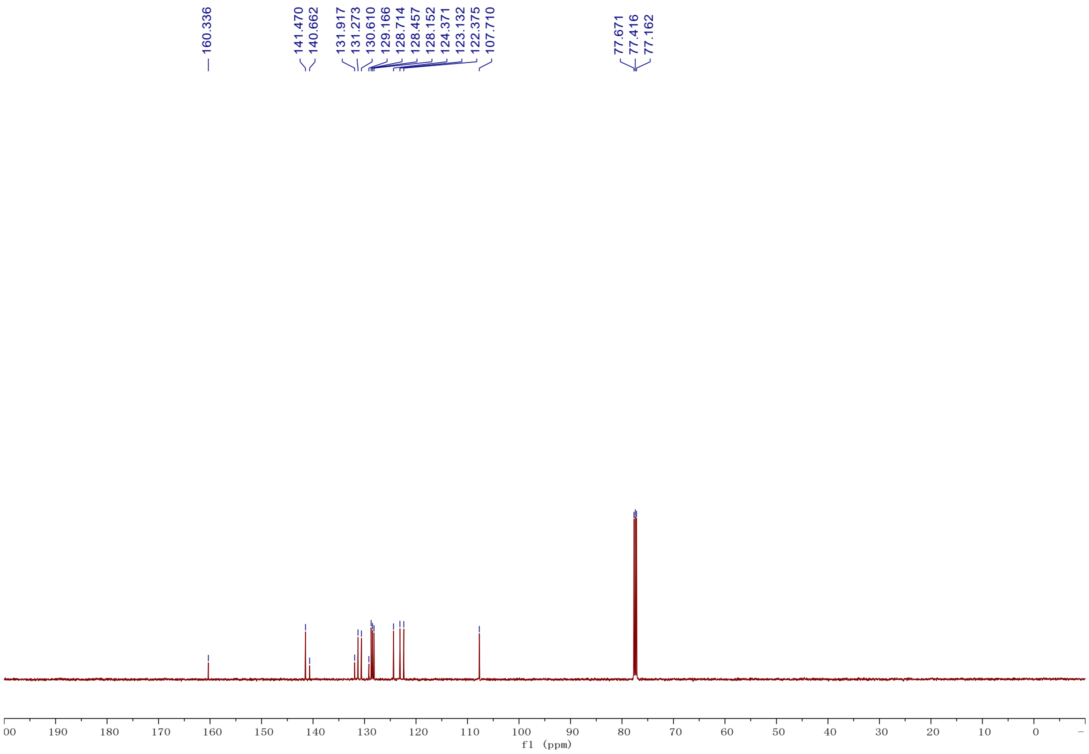

## $^{1}$H NMR and $^{13}$C NMR Spectra of Compound 3av

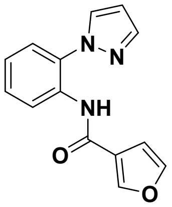

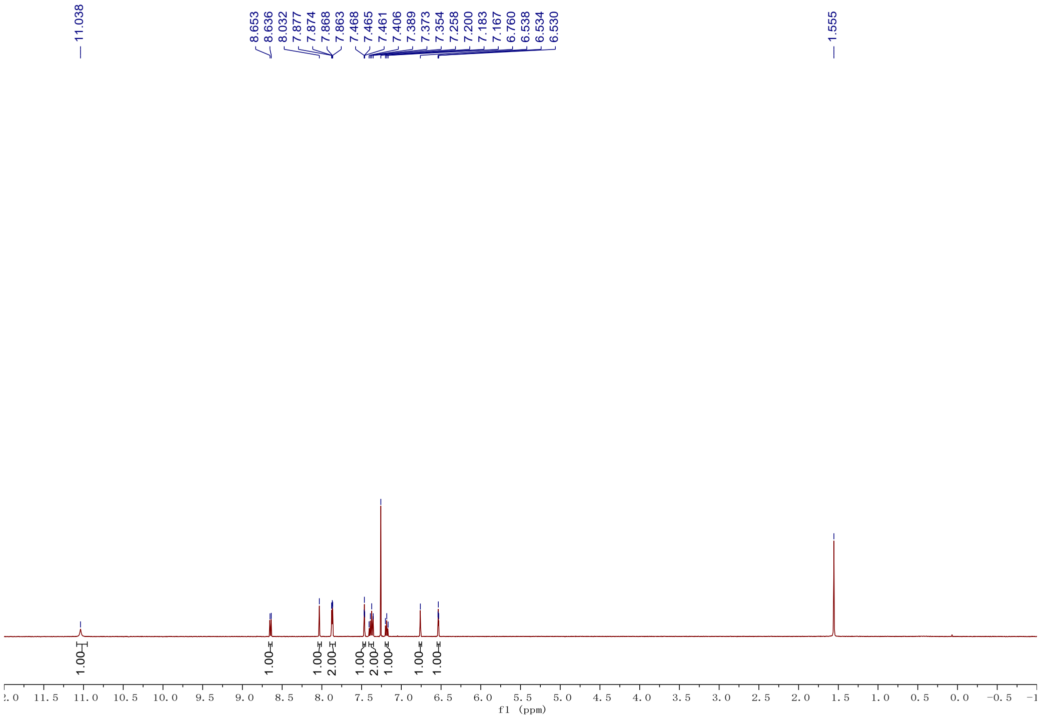


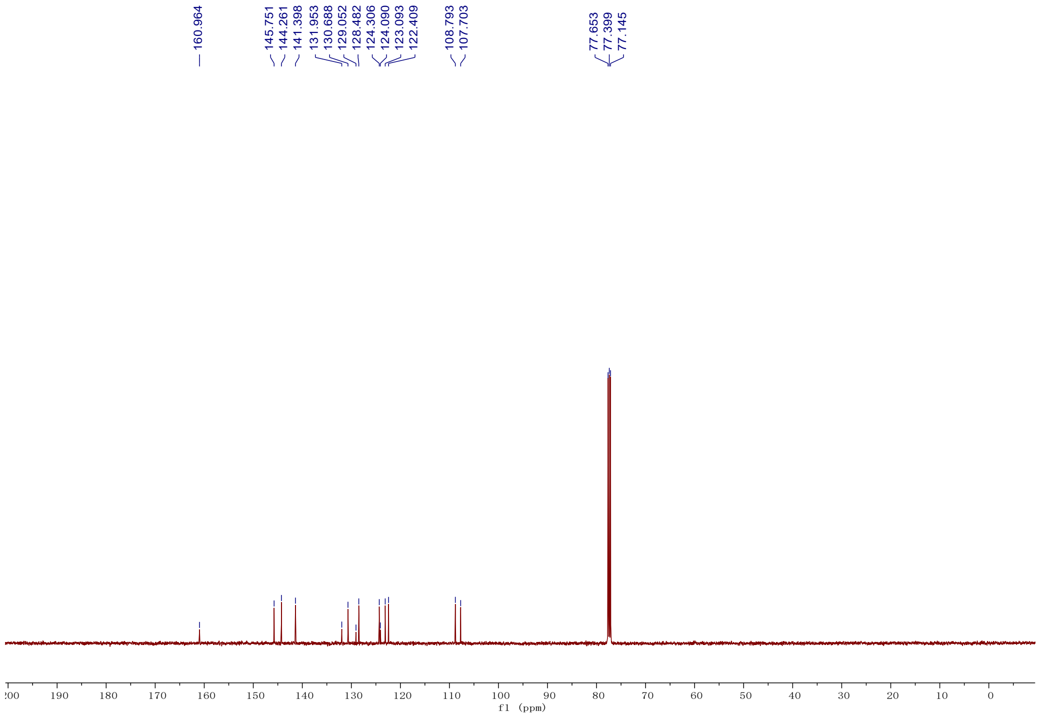

## $^{1}$H NMR and $^{13}$C NMR Spectra of Compound 3aw

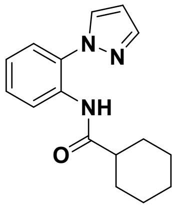

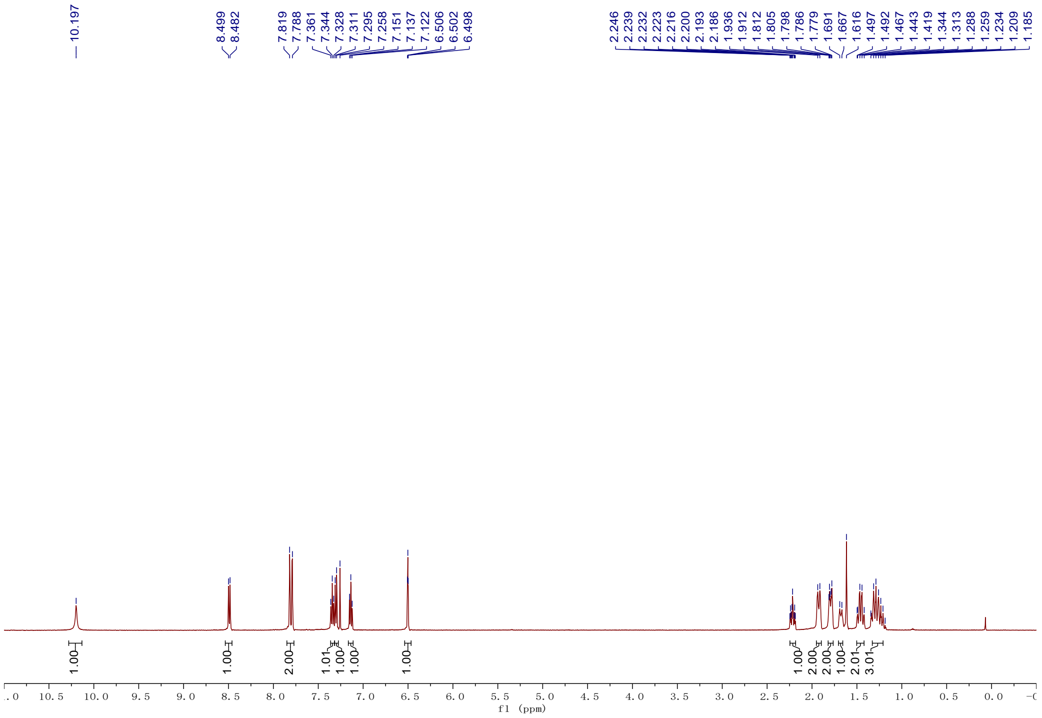


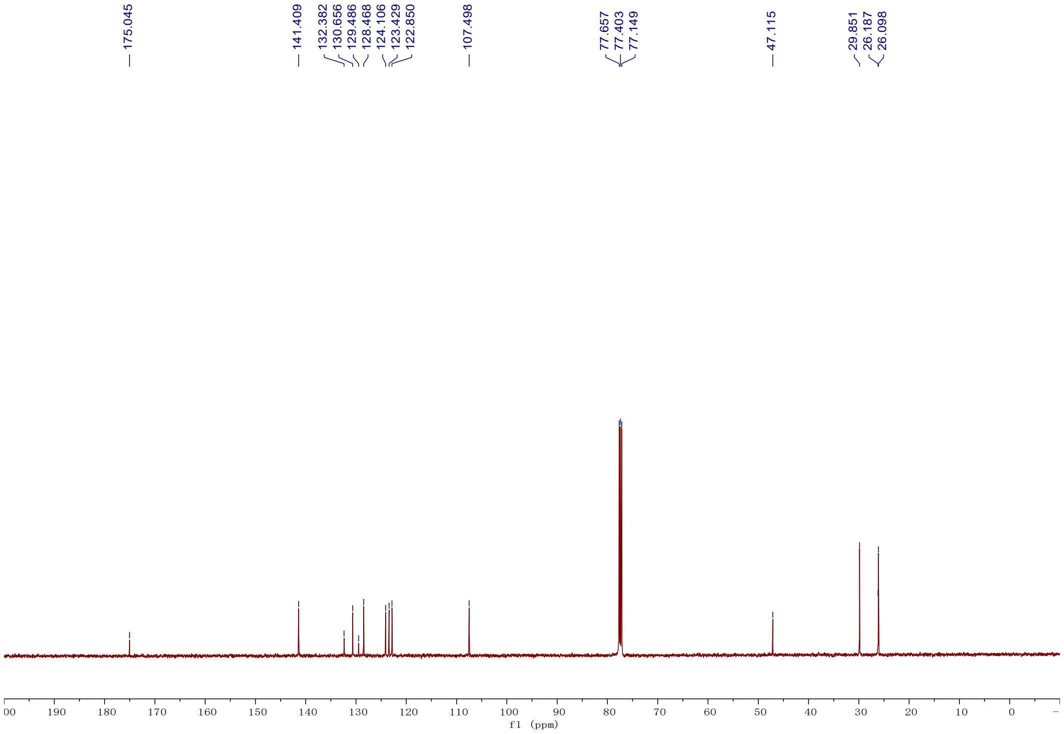

Supplement: RA-014-D4RA00517A-s001 [file RA-014-D4RA00517A-s001.pdf]
